# Proteomic dissection of LPS-inducible, PHF8-dependent secretome reveals novel roles of PHF8 in TLR4-induced acute inflammation and T cell proliferation

**DOI:** 10.1038/srep24833

**Published:** 2016-04-26

**Authors:** Özgün Erdoğan, Ling Xie, Li Wang, Bing Wu, Qing Kong, Yisong Wan, Xian Chen

**Affiliations:** 1 Department of Biochemistry and Biophysics, School of Medicine, University of North Carolina at Chapel Hill, Chapel Hill, North Carolina 27599, US; 2 Department of Chemistry, Fudan University, Shanghai, China; 3 Departement of Microbiology and Immunology, School of Medicine, University of North Carolina at Chapel Hill, Chapel Hill, North Carolina 27599, US; 4 Lineberger Comprehensive Cancer Center, University of North Carolina at Chapel Hill, Chapel Hill, North Carolina, 27599, US

## Abstract

Endotoxin (LPS)-induced changes in histone lysine methylation contribute to the gene-specific transcription for control of inflammation. Still unidentified are the chromatin regulators that drive the transition from a transcriptional-repressive to a transcriptional-active chromatin state of pro-inflammatory genes. Here, using combined approaches to analyze LPS-induced changes in both gene-specific transcription and protein secretion to the extracellular compartment, we characterize novel functions of the lysine demethylase PHF8 as a pro-inflammatory, gene-specific chromatin regulator. First, in the LPS-induced, acute-inflamed macrophages, PHF8 knockdown led to both a reduction of pro-inflammatory factors and an increase in a transcriptional-repressive code (H3K9me2) written by the methyltransferase G9a. Through unbiased quantitative secretome screening we discovered that LPS induces the secretion of a cluster of PHF8-dependent, ‘tolerizable’ proteins that are related to diverse extracellular pathways/processes including those for the activation of adaptive immunity. Specifically, we determined that PHF8 promotes T-cell activation and proliferation, thus providing the first link between the epigenetic regulation of inflammation and adaptive immunity. Further, we found that, in the acute-inflamed macrophages, the acute-active PHF8 opposes the H3K9me1/2-writing activity of G9a to activate specific protein secretions that are suppressed by G9a in the endotoxin-tolerant cells, revealing the inflammatory-phenotypic chromatin drivers that regulate the gene-specific chromatin plasticity.

Activation of inflammation, the key host innate immune response to microbial challenge^1^, is a double-edged sword; it protects the host from infection and cellular damage, yet, its deregulation contributes directly to various inflammation-associated pathologies^2^,^3^. Previous studies indicated that control of inflammation is achieved by endotoxin- or lipopolysaccharide (LPS)-induced gene-specific chromatin modifications. The landscape of promoter chromatin modifications is differentially programmed for a class of pro-inflammatory or “tolerizeable” (T-class) genes, in correlation with either *acutely* or *chronically* inflamed nature or “inflammatory-phenotype” of the stimulated cells^4^. However, how inflammation-phenotypic plasticity is regulated within the chromatin of pro-inflammatory genes is poorly understood.

The properties of histones, the core components of chromatin, can be altered by different post-translational modifications (PTMs) that specify whether the promoter of associated gene is in an open/active or closed/repressed chromatin state, thereby dictating specific biological outcomes such as an inflammatory response^5^. Histone lysine methylation (Kme) patterns indicate chromatin architecture/state of either activated or repressed transcription of associated genes. Particularly on histone H3, methylated H3K9 and H3K27 are associated mostly with *gene repression*^6^. Meanwhile, in a defined chromatin state, the level of each Kme is tightly regulated by specific lysine methyltransferases (KMTs) and lysine demethylases (KDMs). The KMT G9a plays a critical regulatory role in promoting ET by di-methylating H3K9 in the promoters of “T-class” genes^7^ and by changing the global Kme landscape, chromatin remodeling, and activities of select transcription factors^8^. Meanhwile, we discovered that protein phosphatase 2A (PP2Ac) is a broad chromatin-associated regulator of ET, which participates in the establishment of inflammatory-phenotypic chromatin modifications for specific classes of genes^9^: in ET macrophages plant homeodomain finger protein 8 (PHF8), a histone KDM, contains notable PP2Ac-target sites.

PHF8 is a Jumonji-C-domain-containing KDM that demethylates H3K9me1/2 or H3K27me2 or H4K20me1/2^10^, and functions as a transcriptional regulator mostly in brain development, cell cycle, cytoskeleton, and cell proliferation^11^,^12^,^13^. However, little is known about its inflammation-associated function. A recent study indicates a phosphorylation-dependent activation of PHF8 for erasing H3K9me2^12^. Our coincident finding that chronic-active PP2Ac targets the same serine residue whose phosphorylation is associated with activated PHF8 has generated a hypothesis that PP2Ac-mediated dephosphorylation of PHF8 would inhibit its KDM activity in ET macrophages. By performing various biochemical, biological, and immunological experiments, we discovered that the H3K9me2-erasing activity of PHF8 defines the inflammatory phenotype of the macrophages exposed to an acute LPS stimulation through reprogramming chromatin modifications that favor transcriptional activation of pro-inflammatory genes.

Because the end products of the host innate immune response are specific proteins secreted from inflammatory cells that play a direct messenger role in regulating overall immunity^14^, we designed an unbiased label-free-quantitative (LFQ) proteomic experiment^8^,^15^ to systematically investigate the extracellular functions of PHF8. By using LFQ to compare the time-resolved profiles of proteins that are secreted from paired wild-type (WT) *versus* PHF8 knock-down (PHF8-KD) RAW 264.7 cells following LPS stimulation, we identified a novel cluster of the ‘tolerizable’ (T-class) proteins that were secreted in an LPS-inducible, PHF8-dependent manner. We then systematically revealed that PHF8 is a pro-inflammatory chromatin regulator of a broad range of the genes and associated biological processes/pathways. This dataset of the LPS-inducible, PHF8-dependent, T-class secretome not only identifies a large number of PHF8-regulated pro-inflammatory cytokines, but also extends our knowledge of novel PHF8 functions that regulate acute inflammation and overall immunity, including the activation of adaptive immunity.

For the first time, we identified the epigenetic regulatory link between the innate immune response and the activation of adaptive immunity where the LPS-induced secretion of specific proteins involved in the associated T-cell activation/proliferation is PHF8-dependent. Our data also showed that, under an acute inflammatory condition, the gene-specific-repressive function of the Kme writer G9a is antagonized by the Kme eraser PHF8, elucidating the mechanism underlying the gene-specific chromatin plasticity that corresponds to changes in cellular immune responses to LPS stimulation(s). Our quantitative proteomic strategy to dissect LPS-inducible, inflammatory-phenotypic secretome has led us to discover novel PHF8 functions that control inflammation and overall immunity at the post-translational level. These findings are highly physiological-relevant and are not accessible by conventional transcriptome approaches. Thus, this secretome screening method generates the simultaneous multi-target quantitative datasets without the need of antibodies on mass spectrometry, which is otherwise equivalent to that of hundreds or thousands of ‘western blots’ or ELISA.

## Results

### The KDM activity of PHF8 is altered or suppressed by PP2Ac in the reprogramed chromatin under chronic inflammatory conditions

**Figure 1:**
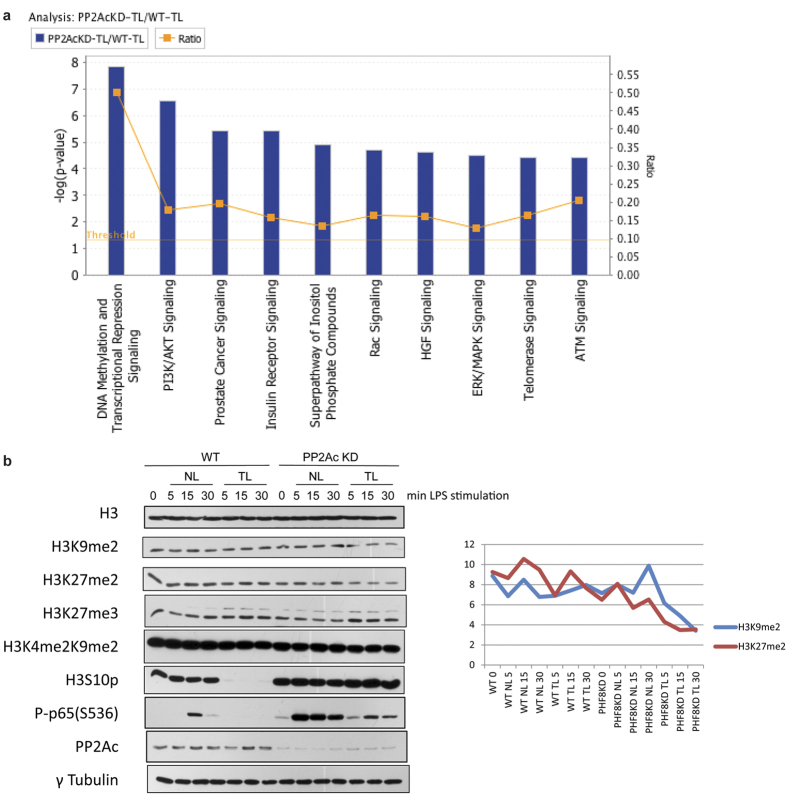
The KDM activity of PHF8 in the chromatin regulator complexes is altered or suppressed by PP2Ac under chronic inflammatory conditions (**a**) IPA network analysis revealed the canonical pathways involving the chromatin proteins that showed the highest increase in PP2Ac-dependent, site-specific phosphorylation in LPS-tolerant macrophages. (**b**) Immunoblot analysis of the effects of the inflammatory phenotype-specific levels of various Kme sites altered by PP2Ac knockdown (PP2Ac-KD). Paired WT and PP2Ac-KD RAW cells were respectively stimulated by LPS for indicated length (0, 5, 15, and 30 min) (Left). The level changes of PHF8-target Kme were quantitatively determined by a scanning densitometry (Right, H3K9me2 in blue and H3K27me2 in red). ‘NL’ refers to acutely stimulated, and ‘TL’ indicates the re-stimulation of the primed macrophages. This immunoblot is a representation of 3 biological replicates, and individual gels were separately run under the same experimental conditions.

To determine the pathways and biological processes (BPs) that are targeted/modulated by PP2Ac, we used an amino-acid-coded mass tagging (AACT)-based quantitative phosphoproteomic approach^16^ to comparatively analyze changes in site-specific phosphorylation levels in WT *versus* PP2Ac-KD RAW 264.7 cells, which led to identifications of the protein substrates directly or indirectly targeted by chronically activated PP2Ac (Supplementary Fig. 1a). Multiple protein components involved in multiple chromatin-associated BPs showed significantly enhanced phosphorylation in PP2AcKD-TL compared to WT-TL, indicating that each of these proteins contains phosphorylation site(s) that could be dephosphorylated by chronically active PP2Ac in ET macrophages (Fig. 1a and Supplementary Fig. 1b). Some of these proteins, including Histone deacetylases 1/2 (HDAC1/2), DNA methyltransferase 1 (DNMT1), and methyl-CpG binding protein 2 (MeCP2), were previously characterized as the major components of co-repressor complexes. Our quantitative phosphoproteomic data showed that PP2Ac dephosphorylates the transcription-regulating S421/423 pair of HDAC1^17^ to inhibit dimerization with HDAC2 in the transcriptionally repressive chromatin state (Supplementary Fig. 1c, **top**). Also, we found that the epigenetic regulator MeCP2 was dephosphorylated by PP2Ac at phosphoS80 that is associated with induction of apoptotic genes^18^ (Supplementary Fig. 1c, **bottom**). Strikingly, associating with these PP2Ac-mediated chromatin reprogramming, we identified three serine residues (S768, S820, and S843) within or near the serine-rich region of the PHF8 (Supplementary Fig. 1d) that showed increased phosphorylation in PP2AcKD-TL compared with phosphorylation in WT-TL (Supplementary Fig. 1e,f), indicating that chronically activated PP2Ac may dephosphorylate PHF8 in ET.

To determine if the KDM activity of PHF8 could be affected in ET macrophages by chronic-active PP2Ac, we examined how knockdown of PP2Ac correlates with the methylation level of these PHF8-target Kme sites, the transcriptionally repressive PTMs in particular, including H3K9me1/2 and H3K27me2^10^. As shown in Fig. 1b, PP2AcKD caused decreased levels of both H3K9me1/2 and H3K27me2 compared with WT cells, and the decrease in the site-specific methylation on H3 was more dramatic under ET (Note the lanes at 15 and 30 min comparing PP2Ac-KD *versus* WT). Notably, the level of H3K27me3, which is not a target of PHF8, remained unchanged as expected, indicating that the KDM activity of PHF8 is indeed suppressed in the PP2Ac-dependent way specifically under the ET chronic inflammatory condition.

### PHF8 erases the transcriptionally repressive H3K9me2 and up-regulates NFκB-dependent pro-inflammatory genes in the acutely inflamed macrophages

As the first step to characterize the inflammatory-associated function of PHF8, we first established a pair of stable cell lines expressing shRNA against GFP (shCON or WT) or against PHF8 (shPHF8 or PHF8-KD). We used immunoblotting to confirm the inflammatory-phenotype of PHF8-KD (Supplementary Fig. 2, **left**), as PHF8-KD efficiency was found at 70% by quantitative PCR (qPCR) (Supplementary Fig. 2, **right**). Also, immunoblotting revealed that in WT cells PHF8 mRNA and protein levels both decreased with 24 hours of LPS stimulation, supporting our data-derived hypothesis that PHF8 is suppressed with prolonged LPS stimulation or in ET.

**Figure 2:**
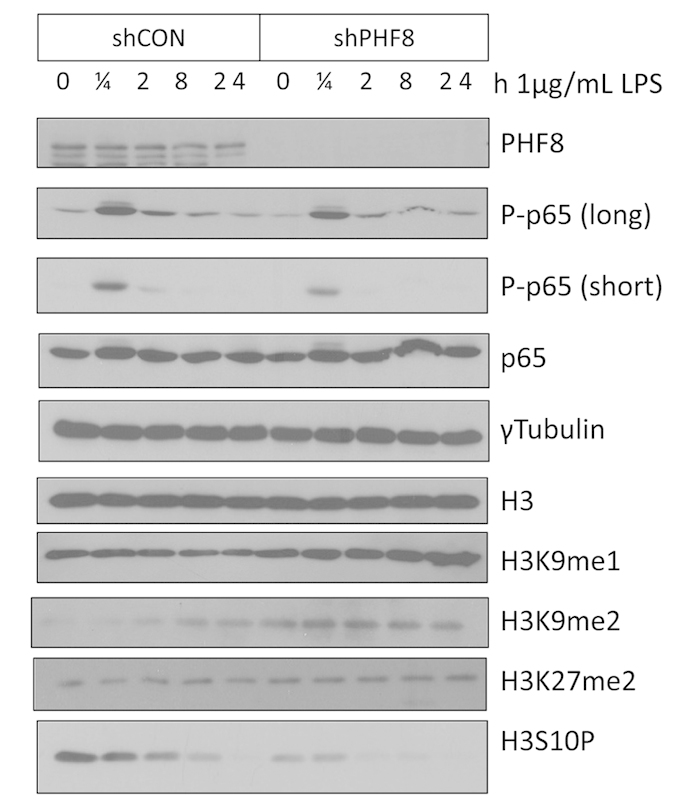
PHF8 plays a pro-inflammatory role in LPS-induced, acutely inflamed macrophages by reducing the levels of H3 K9me1/2. Site-specific lysine methylations on H3 were comparably analyzed in paired WT (shCON) versus PHF8-KD (shPHF8) RAW 264.7 cells using immunoblotting. The macrophages were collected at the indicated time points of LPS stimulation (1/4, 2, 8, and 24 h). This immunoblot is a representation of 3 biological replicates, and individual gels were separately run under the same experimental conditions.

Because both the levels of phosphoserine 10 (H3pS10) and K9me1/2 of histone H3 specifically mark the transcriptional activity status of select genes^4^, we examined the LPS-induced time-dependent changes in the H3pS10 and PHF8-target Kme sites by performing immunoblotting experiments. First, we observed that at each time point following an acute LPS stimulation the LPS-induced level of H3pS10 was significantly lower in PHF8-KD cells vs. WT cells (Fig. 2), indicating *knocking down PHF8 reduces the overall inflammatory response of LPS-stimulated macrophages*. Further, in the WT macrophages, among the PHF8-targeted Kme sites on histone H3, both H3K9me2 and H3K9me1 showed significant decreases at the early time points following an acute LPS stimulation (Fig. 2), in contrast to the increased levels *at the same time points* in PHF8-KD cells. Meanwhile, the level of H3K27me2 slightly decreased.

**Figure 3:**
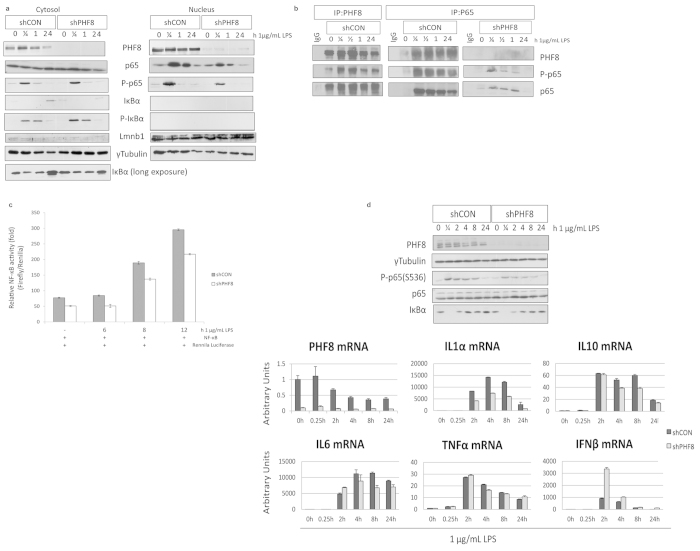
PHF8 is a positive chromatin regulator of NFκB transactivation. **(a)** Immunoblot analysis of the p65 and P-p65 abundance in the cytosol and nucleus of paired WT (shCON) and PHF8-KD (shPHF8) cells with LPS stimulation for indicated duration (0, 1/4, 1, and 24 h). The gels were run under same experimental conditions and sample loading. **(b)** PHF8 forms a nucleus-specific complex with p65 in the acutely LPS-stimulated WT cells, and the complex formation is enhanced with an acute LPS stimulation. Immunoprecipitations using the antibody against p65 or PHF8 were performed respectively for the nuclear fraction (Fig. 3a, **left**) of WT (shCON) versus PHF8-KD (shPHF8) RAW cells were collected at the indicated time points following an acute LPS stimulation (1/4, 1/2, 1, 24 h). Immunoblotting experiments were conducted with indicated antibody. **(c**) PHF8-KD leads to reduced NFκB activity. 293-TLR4-MD2-CD14 cells stably expressing WT (shCON) or PHF8-KD (shPHF8) were transfected with Firefly luciferase. 24 hours after the transfection, the cells were stimulated with 1μg/ml LPS and were collected at 0, 6, 8, or 12 h with three biological replicates. The activity of each sample was normalized to Renilla luciferase. Each column shows mean ± s.e. of at least three independent experiments. *P < 0.05 compared with mock-transfected cells (Student’s *t*-test). **(d)** PHF8 regulates the expression of select pro-inflammatory cytokines. Immunoblot analysis was conducted to monitor indicated inflammatory markers (top). qPCR analysis (bottom) shows the mRNA expression of select proinflammatory cytokines in paired WT (shCON, dark grey bars) and PHF8-KD (shPHF8, light grey bars) with LPS stimulation while error bars show the mean ± s.e. of three independent experiments. Time points are indicated for the non-stimulated (0 h) and stimulated RAW cells (0.25, 2, 4, 8, and 24 h).

Phosphorylation of p65 at Ser536 (P-p65) marks the pro-inflammatory transactivation of the transcription factor NFκB as well as the accessibility of NFκB to select gene promoters^19^. Our previous study showed that chronically active PP2Ac dephosphorylates Ser536, leading to reduced transactivation and promoter accessibility by NFκB^9^. Here, while the total p65 amount was similar in both WT and PHF8-KD, the LPS-inducible time-dependent level of P-p65 was lower in PHF8-KD than in WT (Fig. 2, Supplementary Fig. 2). Further, we found that this decrease in p65 phosphorylation upon PHF8 knockdown occurred primarily on the p65 that was translocated into the nucleus (Fig. 3a). To clarify whether PHF8 affects p65 phosphorylation, either directly or indirectly, we examined p65 or PHF8 immunoprecipitates (Fig. 3b): P-p65 was maximally phosphorylated in the PHF8 immunoprecipitate from the macrophages following acute LPS stimulation (30 min), but reduced with prolonged stimulation (Fig. 3b). Since NFκB activity is regulated by lysine methylation^20^, our results suggested that PHF8 may promote the transactivation of NFκB during acute inflammation through PHF8-mediated lysine demethylation of NFκB. This is also supported by a recent report that another KDM PHF20 maintains the active state of NFκB^21^.

We then evaluated the contribution of PHF8, or the NL-specific interaction between PHF8 and p65, on LPS-induced transcriptional activity of p65 by performing dual-luciferase reporter assays. Clearly, the LPS-induced NFκB activity was reduced in the PHF8-KD cells compared to WT cells, indicating that PHF8 positively regulates LPS-induced transcriptional activity of NFκB in acutely inflamed cells (Fig. 3c).

Since PHF8 directly regulates LPS-inducible, phosphorylation-dependent transactivation of NFκB in acutely inflamed cells, we next investigated the impact of PHF8 activity on the transcription of select pro-inflammatory genes. Thus, we compared the cytokine expression of paired WT and PHF8-KD cells with either no stimulation or 1 mg/mL LPS stimulation for 1/4, 2, 4, 8, and 24 h (Fig. 3d). First, we confirmed the inflammatory phenotype of each time point by immunoblotting the indicated markers (Fig. 3d**, top**), followed by measuring mRNA levels of the indicated genes. In WT macrophages, LPS stimulation increased the mRNA expression of pro-inflammatory cytokines whereas in PHF8-KD cells we observed a decrease in most of these cytokines (Fig. 3d**, bottom**). PHF8 mRNA expression paralleled cytokines expression; in WT cells, PHF8 mRNA expression following LPS stimulation was high until 2 h, then decreased below basal levels after 2 h, and was lowest after 4 h. This time course supports the notion that PHF8 functions as a transcriptional activator of select cytokines upon acute LPS stimulation; its expression is decreased in ET to increase repressive H3K9me2 in conjunction with immunosuppression. Importantly, Interfeuron β (IFNβ), which was the only cytokine to show significantly higher LPS-induced mRNA expression in the PHF8-KD cells, can act as an inhibitor of inflammation^22^. Thus, in PHF8-KD cells, the increased mRNA expression of IFNβ in combination with decreased mRNA expression of pro-inflammatory cytokines implies that PHF8-dependent activation of cytokines is both gene-specific and pro-inflammatory phenotype-specific. Because these cytokines are regulated by NFκB, our data demonstrate that PHF8 positively regulates LPS-induced, NFκB-dependent transcription of select pro-inflammatory genes. It should be noted that different cytokines/chemokines exhibited activated or PHF8-dependent mRNA expression at different time points after LPS stimulation.

### PHF8 selectively promotes the secretion of a specific group of ‘tolerizable’ proteins including cytokines and chemokines

**Figure 4:**
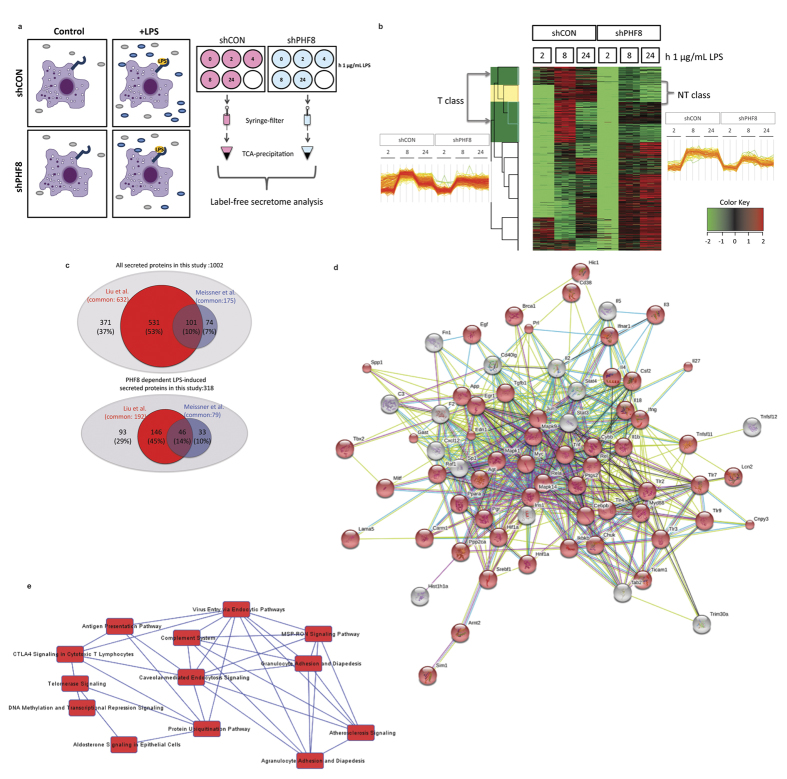
PHF8 regulates a broad range of the extracellular functions of acute LPS-stimulated macrophages through modulating the LPS-induced secretion of specific class of proteins. (**a**) Workflow and experimental design of LFQ secretome analysis to identify/profile the proteins that are differentially secreted into the media from paired WT (shCON) versus PHF8-KD (shPHF8) cells with LPS stimulation. WT and PHF8-KD RAW cells were cultured in serum-free media and stimulated with LPS for 0, 2, 4, 8, and 24 h. The extracellular media was used for MS/MS while cell pellets were used to confirm inflammatory phenotype as shown in Supplementary Fig. 3a. (**b**) The LFQ secretome heatmap of the differentially secreted proteins from paired WT (shCON) and PHF8-KD (shPHF8) RAW 264.7 macrophages under different inflammatory conditions. The color key (right bottom) indicates LPS-induced changes (increase in red, decrease in green) in secretion of proteins in logarithmic scale. Time points are indicated for the non-stimulated (0 h) and stimulated cells (8 and 24 h). (**c**) The comparison of the secretome analysis with previous BMDM secretome studies^8^,^15^. Top panel shows the comparison of all secreted proteins while the bottom panel shows the comparison of LPS-induced secreted proteins. The present study of the secretome is represented by a light grey circle while the Liu *et al*.^8^ and Meissner *et al*.^15^ secretome are represented by red and blue circles, respectively. (**c**) The overall protein-protein interaction network of the T-class secretome that belong to the highly enriched GOBP/GOCC/GOMF is composed mostly of response signaling BP (red nodes). (**d**) IPA network analysis showing the interplaying pathways that cross-talk with high confidence (p < 0.05).

To understand how PHF8 directly regulates LPS-induced inflammation and overall immune response and to identify the associated BPs and pathways, we employed an unbiased LFQ proteomic method^8^,^15^ to profile the LPS-induced, time-resolved proteins that are differentially secreted from paired WT versus PHF8-KD RAW cells (Fig. 4a). This non-biased, discovery-driven secretome screening can identify not only PHF8-regulated secretory proteins but also unknown extracellular functions of PHF8 that control inflammation. We therefore stimulated WT and PHF8-KD RAW cells with LPS for 0, 2, 4, 8, and 24 h; representing non-stimulated (N), acutely stimulated (NL), and prolonged stimulated (T) inflammatory states (Fig. 4a). Following our protocol^8^, we collected the culture supernatants containing proteins secreted from WT and PHF8-KD cells with either N, or NL, or T phenotype, while the cell pellets were assayed by immunoblotting for the levels of phospho-p65, IκBα, and phospho-IκBα (Supplementary Fig. 3a). An LFQ analysis was then performed on two biological replicates, in which each biological set was further measured with three technical replicates. The time-resolved or inflammatory-phenotypic changes in the amounts of secreted proteins were filtered to proteins with a 5% permutation-based false-discovery-rate (FDR) and normalized for all inflammatory states according to the Z-score. The correlation between different replicates was confirmed with a Pearson correlation analysis (score >0.7) (Supplementary Fig. 3b).

We then performed hierarchical clustering analysis for the 1002 secreted proteins identified and quantified by LFQ (Supplementary Data 1). As shown in Fig. 4b, we found that 368 proteins in WT cells showed LPS-inducible, time-dependent increases, whereas in PHF8-KD cells 318 of these proteins did not increase, indicating that their LPS-inducible secretion is PHF8-dependent.

Further, approximately 254 proteins (80% of LPS-inducible secretome) showed a secretion pattern similar to the inflammatory phenotype-specific mRNA expression of T-class genes^4^; prolonged LPS stimulation caused a reduction in secretion of proteins that has been increased by an acute LPS stimulation (Fig. 4b**, left**). Therefore, we defined this cluster of LPS-inducible, secretory proteins as the ‘tolerizable’- or ‘T-class secretome’ (highlighted in green in the cluster column) (Supplementary Table 1). The remaining 64 LPS-inducible proteins (Fig. 4b**, right**) were secreted in a trend similar to the mRNA expression of the non-tolerizable (NT)-class genes (highlighted in yellow in the cluster column), which are clustered as the ‘NT-class secretome’ (Supplementary Table 2).

To benchmark the LFQ secretome profiling for validation, we compared our data of the Raw cell secretome with that of the LPS-inducible secretome from primary bone marrow-derived macrophages (BMDM)^15^. This comparison revealed 175 secretome components in common (Fig. 4c, Supplementary Table 3), 40% of which are cytokines and chemokines (Table 1). Further, a comparison with a more recent BMDM secretome^8^ revealed 632 common components (Fig. 4c, Supplementary Table 4), 192 of which (30%) were found in the LPS-inducible portion including most of the cytokines and chemokines (Table 1). Some defense or wounding response-related proteins were found secreted upon LPS stimulation, including multiple cytokines/chemokines, complement factors, Integrin beta 2 (Itgb2), Interleukin 27 (IL27), and lymphocyte antigen 86 (Ly86), in line with a recent report indicating the regulatory role of PHF8 in wound healing by bone-marrow stromal cells^23^.

These overlapping results not only validated the accuracy of our LFQ secretome screening, but also, more importantly, indicated that the acute LPS-induced secretion of select cytokines/chemokines, and many other immune response-related proteins, is PHF8-dependent, supporting the conclusion that PHF8 is the primary *re-programmer* of chromatin modifications associated with various inflammatory genes specifically in acutely inflamed macrophages.

### The PHF8-regulated, T-class secretome contains proteins involved in diverse extracellular processes and pathways

Our secretome data suggested that acutely activated PHF8 regulates the secretion of a broad range of proteins that are involved in diverse BPs/pathways. In addition to our findings, we systematically explored novel pathways through which PHF8 may regulate overall immunity. By using the David bioinformatics database^24^ in the context of Gene Ontology (GO) BPs (GOBP), GO Cellular Components (GOCC), GO Molecular Functions (GOMF), and Kyoto Encyclopedia of Genes and Genomes (KEGG) pathways, we comprehensively analyzed the functional categories of the LPS-inducible, PHF8-dependent, T-class secretome, which constitutes more than 80% of all the PHF8-dependent secretome.

The GOBP enrichment of the T-class PHF8-dependent secretome revealed defense response, response to wounding, immune/inflammatory response, antigen processing and presentation, regulation of complement factors, cell metabolism, glycolysis-related cell adhesion, cell migration, cell-to-cell communication, and T-cell migration (Supplementary Fig. 3c). The enrichment analyses of GOCC and KEGG categories from the same dataset revealed similar categories of the extracellular functions that involve these secreted proteins (Supplementary Fig. 3d,e) Most of these proteins also showed LPS-inducible secretion from BMDMs^8^,^15^, indicating the high purity of our secretome sample preparation and the accuracy of our LFQ approach. Some components identified in the T-class PHF8-dependent secretome that represent each of the major GOBP/GOCC/GOMF/KEGG categories are described below.

### PHF8 is a broad chromatin regulator of multiple BPs and pathways primarily associated with the activation of adaptive immunity

#### PHF8 regulates processing to antigens from the products of apoptotic-inflamed cells

Multiple members of one of the major antigen processing/presentation complexes, the minichromosome maintenance (MCM) protein complex, were found as a highly enriched GOBP in the T-class PHF8-dependent secretome (Supplementary Fig. 3c). MCMs were expressed on the surface of different types of malignant/proliferative cells^25^ but there is little knowledge of how they are regulated during a pro-inflammatory response. Here, the MCM complex constituted the pathways associated with DNA replication in enriched KEGG pathways (Supplementary Fig. 3e). Because DNA replication is an inflammatory process^26^, PHF8 may regulate DNA metabolism of pathogens in the extracellular matrix via regulating the secretion of MCM complexes. Coincidently, identification of multiple nucleosome and chromosome organization-related proteins including Poly [ADP-ribose] polymerase-1 (PARP-1)^27^ in the secretome indicated that acute LPS stimulation increased cell death.

#### PHF8 regulates lysosome activation

KEGG pathway enrichment of the T-class PHF8-dependent secretome revealed metabolic pathways, antigen processing and presentation, and lysosomal pathways as exclusively T-class-specific (Supplementary Fig. 3e). Lysosome-related secreted proteins were mostly peptidase family members, which were previously identified in LPS-induced macrophage secretome^8^,^15^,^28^, are part of the antigen processing and presentation pathways and are involved in multiple immunity-related processes^29^. The existence of these lysosome-related proteins only in the T-class PHF8-dependent secretome indicates that PHF8 regulates lysosome formation and activity upon LPS stimulation.

#### PHF8 regulates antigen presentation mediated by MHC expression

In GOCC analysis (Supplementary Fig. 3d), we also identified major MHC components in the PHF8-dependent, T-class secretome, in agreement with MHC enrichment in GOBP (Supplementary Fig. 3c), implying that the activity of PHF8 is crucial for MHC expression on the cell surface. Expression and presentation of bacterial antigens on the cell surface by MHC1 complex in response to LPS is crucial for the interaction between innate immunity and adaptive immunity. MHC components and pathogenic antigens are recognized by T cells, which leads to a cascade of inflammation-related responses that include death of the infected cells, death of the bacteria inside macrophage vesicles, and B cell activation to eliminate extracellular pathogens^30^.

#### PHF8 promotes adhesion, communication, and migration of the immune cells involved in adaptive immunity by regulating the secretion of the corresponding factors

We also found that PHF8 affected the LPS-induced secretion of select adaptive immunity regulators, including Complement factor b (Cfb), Complement factor h (Cfh), Interleukin enhancer binding factor 3 (Ilf3)^31^, Ly86^32^, Amyloid beta precursor protein (APP)^33^, integral membrane protein 2B (Itmb2), and metalloproteinase domain-containing protein 8 (ADAM8), and ADAM17. Specifically LPS-induced secretion of complement factors from macrophages is important for both recognition of pathogens and activation of adaptive immunity^34^, indicating the regulatory role of PHF8 in these BPs. Like Meissner *et al*.^15^, we identified ADAM17 as an LPS-induced macrophage secretome component; ADAM17 is an adaptive immunity regulator responsible for shedding of membrane-bound proteins, one such shed protein being APP^35^. The presence of ADAM17 with APP in the LPS-induced secretome confirms APP shedding from the membrane.

GOBP analysis (Supplementary Fig. 3c) also revealed some components of the PHF8-dependent T-class secretome are involved in cell adhesion and cell communication, including MHC complexes, serglycin (SRGN), Lgals3BP, intercellular adhesion molecule 1 (Icam1), Itga4, Itgb2, Cathepsin B (Ctsb), urokinase plasminogen-activator surface receptor (PLAUR), syndecan 4 (Sdc4), and poliovirus receptor-related 1 (Pvrl1). SRGN and Lgals3BP are cell-cell communication regulators activated by TLR4^36^,^37^. Icam1 is a cell-surface glycoprotein expressed on immune cells that binds to integrins^38^; integrins Itga4 and Itgb2 in turn regulate cell migration and adaptive immunity^39^ and are in common with LPS-induced BMDM secretome^15^. Similarly, the released adhesion molecules Ctsb^29^, PLAUR^40^, SDC4^41^, and Pvrl1^42^ are involved in regulating cell migration in inflammation.

From a system perspective, we next used STRING^43^ to explore the pathway links in the PHF8-dependent, T-class secretome. A protein-protein interaction (PPI) network was mapped among the secreted proteins categorized in different GOBP, GOCC, and GOMF (Fig. 4d); this network was dominated by response to signaling BP as highlighted with red nodes. Based on these protein ‘nodes’ in the PPI network, we used IPA to identify the canonical pathways that share common nodes, which revealed the interplay between pathways involving antigen processing and presentation, complement regulation, cell adhesion, and endocytosis as the coordinator of inflammation response (Fig. 4e).

### PHF8 positively regulates the activation and proliferation of T cells via secretion of specific proteins involved in antigen presentation and activation of adaptive immunity

**Figure 5:**
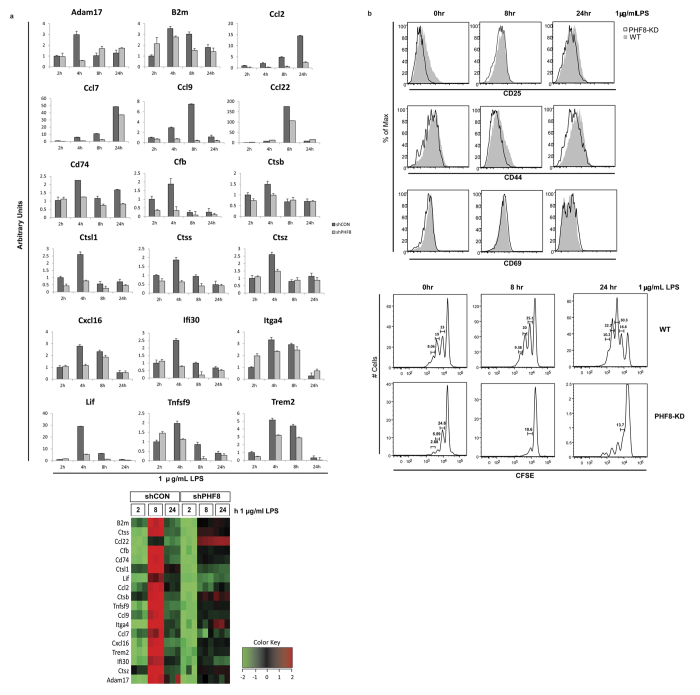
PHF8 promotes the LPS-induced of T cell activation and proliferation by positively regulating the secretion of specific classes of proteins. (**a**) mRNA expression of the selected genes that are related to antigen processing and presentation, and T cell activation in paired WT (shCON) and PHF8-KD (shPHF8) RAW cells. The heatmap showing the quantitative secretion pattern of these proteins found in the LFQ secretome analysis is given at the bottom. (**b**) The histograms obtained from the T-cell proliferation (top) and activation (bottom) assays demonstrate the PHF8 dependence of T-cell proliferation and activation. WT or PHF8-KD RAW cells were stimulated with 1 μg/ml LPS for 0, 8, and 24 h respectively. Then RAW cells are treated with mitomycin C for 30 min and fed with 30 μg/ml GP33–41 peptide for 20 min. CD8^+^ T cells isolated from P14 transgenic mouse were either labeled with CFSE for proliferation assay or kept unlabeled for activation assay, and co-cultured with pretreated RAW cells for 4–6 days. The population distributions of multiple surface markers of T cell activation and proliferation including CD25, CD44, CD69 were analyzed by flow cytometry. The activation marker histograms (top panel) in black line represent the T-cells pretreated with PHF8-KD RAW cells while the histograms in grey represent the T-cells pretreated with WT RAW cells. The numbers on the proliferation tables show the percentage of every division of proliferated cells among total CD8^+^ T cells (Bottom).

Our findings of the secreted proteins associated with the activation of adaptive immunity in the PHF8-dependent secretome (Fig. 4e) implicated that PHF8 positively links innate immunity and adaptive immunity. First, we next examined the LPS-induced mRNA expression of select genes encoding the corresponding T-class, PHF8-dependent secreted proteins based on their relevance to antigen processing/presentation and the activation of adaptive immunity. Among them, Lif is a known regulator of T-cell maturation^44^. Similarly, the cytokine Tumor necrosis factor superfamily member 9 (Tnfsf9) (CD137L) regulates innate and adaptive immunity through activation of CD4^+^ T cells^45^. The chemokines Ccl2, Ccl7, Ccl9, Ccl22, and Cxcl16 activate adaptive immunity by promoting the activation and migration of T cells^46^. Cfb is a complement factor that regulates adaptive immunity response^34^. ADAM17 and beta-2-microglobulin (B2m) regulate T cell proliferation and differentiation^30^,^47^ while CD74 regulates cell survival in adaptive immunity^48^. Triggering receptor expressed on myeloid cells 2 (TREM2) is a T-cell regulator^49^ while Itga4 helps traffic leukocytes to the site of inflammation^39^,^50^. Moreover, knockout of Interfeuron inducible protein 30 (Ifi30) in mice causes decreased CD8^+^ T cell proliferation^51^. As the antigen processing/presentation massagers, cathepsins regulates the formation of CD4^+^ T lymphocytes^29^. As shown in Fig. 5a, all of these genes showed increased mRNA expression 4 hours following LPS stimulation in WT cells while the LPS-induced mRNA increases were significantly lower in PHF8-KD cells. Moreover, with the exception of Ccl2 and Ccl7, mRNA expressions of these genes decreased 8 hours after the LPS stimulation, consistent with the pattern of PHF8-dependent, T-class protein secretion. In agreement with previous findings that the mRNA expression of Ccl2 and Ccl7 persisted even with the prolonged LPS stimulation^52^,^53^, identification of Ccl2 and Ccl7 at the protein level in T-class PHF8-dependent secretome indicates the impact of post-translational events on the activation of adaptive immunity, and shows how quantitative proteomic studies of extracellular proteins can help us immunity regulation.

Further, to determine the immediate impact of PHF8 in activating adaptive immunity, we comparatively measured the T-cell activation and proliferation with incubation with either WT or PHF8-KD Raw cells that were respectively collected at different time points following an acute LPS stimulation. Through monitoring multiple markers of T-cell activation including CD25, CD44, and CD69, we observed that the co-existing acute-inflamed WT cells promoted efficient activation of CD8^+^ T cells while the incubation with PHF8-KD cells gave less activated T cells (Fig. 5b**, top**). Similarly, more proliferated P14 CD8^+^ T cells were found after 6 days of co-incubation while PHF8-KD cells lost the ability to promote the proliferation of T cells with or without LPS stimulation (Fig. 5b**, bottom**), all indicating the regulatory function of PHF8 in the activation of adaptive immunity.

### PHF8 is a G9a-antagonist that regulates gene-specific chromatin states in acute inflammation

Because KMT G9a is the writer of H3K9me1/2 in chronically inflamed macrophages, whereas KDM PHF8 is the H3K9me1/2 eraser in acutely inflamed cells, we compared the ET-specific G9a-dependent secretome^8^ with that of PHF8 during acute inflammation. Interestingly, identical proteins were secreted in an opposite manner. Thus LPS-induced secretion of the same set of proteins as that induced by the G9a inhibitor UNC0638 in ET^8^ or as in the acutely inflamed WT cells. However, these proteins secretions were decreased in PHF8-KD cells (Supplementary Table 5). These results indicate an inflammatory-phenotypic, differential secretion in PHF8- versus G9a-dependent manner under either acute- or ET-inflammatory condition.

The category enrichment analysis identified 41, 33, 25, 21, 17, 14, 13, and 11 secreted proteins, respectively, belonging to translation, immune/inflammatory/defense response, response to wounding, cell proliferation, positive regulation of immune system, and chemotaxis GOBP/GOMFs (Supplementary Fig. 4a, 4b). More importantly, the secretion of many cytokines, chemokines, complement factors, CD14, and CD74 antigens was found to vary between the acute- versus chronic-inflammatory phenotype, depending upon the inflammatory-phenotypic activity of either PHF8 or G9a. Specifically, immune signaling molecules, select cytokines/chemokines, and antigen processing/presentation factors that were identified as major components of the T-class PHF8-dependent secretome showed increased secretion in a strictly PHF8-dependent manner in the acutely inflamed cells, whereas their secretion was suppressed in the G9a-dependent ET macrophages.

Differential secretion was also observed for the proteins involved in translational processes such as tRNA aminoacylation, translation initiation, and translation elongation (Supplementary Fig. 4a, Supplementary Table 6); this observation coincided with the fact that the H3K9me1/2 eraser PHF8 acts as a transcriptional/translational activator to regulate ribosomal RNA transcription^54^. Moreover, the secretion of wound response proteins was found in the same GOBP class (Supplementary Table 6). Some of these proteins function in cell proliferation, indicating that H3K9me1/2-associated secreted proteins modulate multiple BPs related to macrophage cell fate decision. Further, secretion of many proteins associated with cell adhesion, cell migration, and movement-associated cytoskeleton was regulated antagonistically by the inflammatory-phenotypic PHF8 versus G9a activity. Similarly, GOCC enrichment analysis revealed inflammatory-phenotypic compartments such as the lysosome, Golgi-associated vesicles, and MHC complexes (Supplementary Fig. 4c); more importantly, all the PHF8-dependent MHC complex components were commonly identified in the G9a-suppressed secretome.

**Figure 6:**
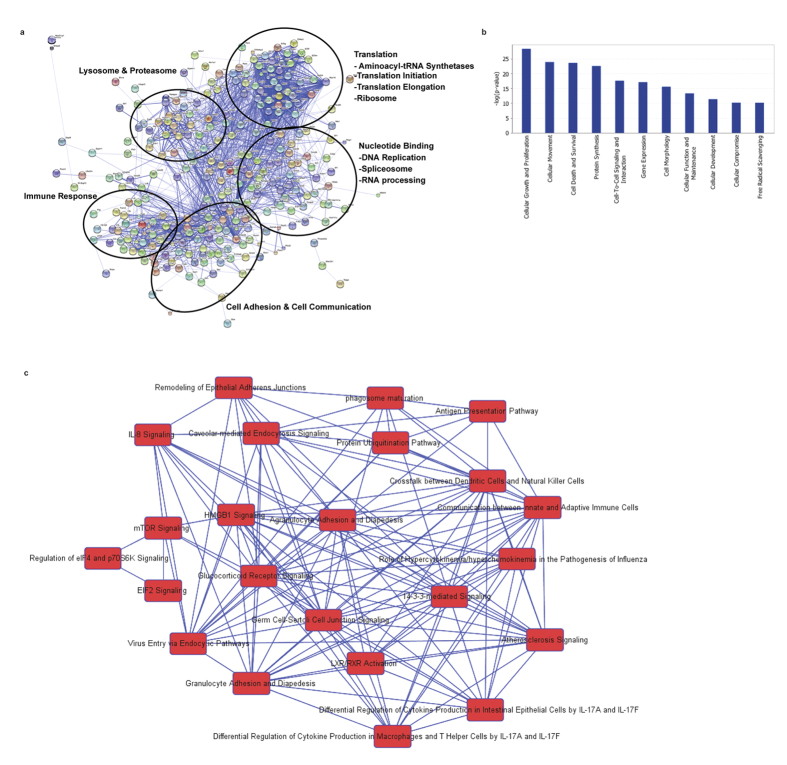
G9a and PHF8 regulate the secretion of similar sets of proteins in an opposite manner in chronically versus acutely inflamed macrophages. (**a**) Protein-protein interaction network of differentially regulated secretome by G9a and PHF8 antagonistically^8^ determined by STRING in high confidence (confidence score 0.7). (**b**) Top 10 canonical pathways regulated antagonistically by G9a and PHF8 according to the inflammatory phenotype determined by IPA network analysis. (**c**) Canonical pathways in cross talk for regulation of different innate immune responses.

To uncover the pathways antagonistically regulated by acutely active PHF8 versus chronically active G9a, we performed STRING PPI analysis on the dataset of the PHF8-dependent, G9a-antagonist secretome. This analysis revealed multiple, interconnected subnetworks linking the signaling of immune response, translational regulation, cell adhesion/communication, nucleotide binding, and lysosome/proteasome (Fig. 6a). Further, IPA canonical pathway analysis revealed cell growth, cell movement, cell death, and cell-cell interaction as macrophage response-dependent pathways regulated by G9a and PHF8 antagonistically (Fig. 6b). Moreover, we identified canonicalpathways that share common protein nodes via IPA, including antigen presentation, the communication between innate and adaptive immune cells, IL-8 signaling, differential regulation of cytokine production, adhesion, mTOR, and Eukaryotic initiation factor 2 (EIF2) signaling indicating that PHF8 and G9a regulate immune response antagonistically through affecting the interplay between these pathways (Fig. 6c).

These results indicated that, in an opposing manner, G9a and PHF8 regulate the secretion of the same set of LPS-inducible genes by determining the methylation level of H3K9 in their associated chromatin.

## Discussion

This report is the first to document the novel function of PHF8 in chromatin-associated inflammation control and the concurrent activation of adaptive immunity. Coincident with our recent discovery that, in the ET macrophages, the KMT G9a more actively coordinates the assembly of chromatin writer complexes in the silent chromatin enriched with the transcriptionally repressive histone H3K9me2 code^8^, we now further reveal that PHF8, the eraser of H3K9me1/2, is *the primary driver* for establishing the transcriptionally active state of the gene-specific chromatin in acutely inflamed cells. For the first time, our data obtained *at the post-translational level* indicated that acutely active PHF8 promotes the LPS-induced secretion of the GOBP/GOMP categories of proteins similar to the proteins that are suppressed by G9a under the chronic inflammatory condition, we conclude that PHF8 is the antagonist of G9a in regulating the gene-specific chromatin state under the acute inflammatory condition.

**Figure 7:**
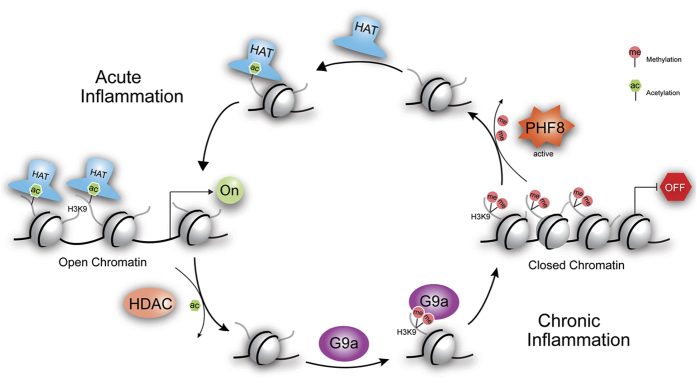
The postulated mechanism underlying the gene-specific chromatin plasticity corresponding to the changes of immune cellular response to LPS stimulation(s). During chronic inflammation, Kme writer G9a and G9a-associated complexes maintain the closed chromatin architecture via H3K9me1/2 enrichment. Under the acute inflammatory conditions the gene-specific-repressive function of G9a is antagonized by the Kme eraser PHF8. HAT: histone acetyl-transferase, HDAC: histone deacetylase, ac: acetylation, and me: methylation.

As shown in Fig. 7, we postulate the mechanism of plasticity of the inflammatory phenotype-specific chromatin modifications that closely correlates with the transcriptional regulation of select classes of genes; the KDM activity of PHF8 is modulated through reversible phosphorylation by differentially activated kinases or protein phosphatase(s) under different inflammatory conditions. Upon LPS-induced acute inflammation, PHF8 erases H3K9me1/2, leading to increased secretion of T-class proteins. In contrast, in ET macrophages, PHF8 KDM activity is suppressed, likely by PP2Ac-mediated dephosphorylation, resulting reduced secretion of the similar set of the proteins. Within the chromatin associated with similar classes of genes, both PHF8 and G9a are the regulators of the LPS-induced chromatin modifications but function *in an opposing manner* under either an acute- or chronic-inflammatory condition.

Interestingly, the same Ser843 (Ser880 in human, Supplementary Fig. 1d–f) of PHF8 that we found targeted by chronically active PP2Ac was reported to be phosphorylated by CDK2/cyclin E kinase, enhancing the KDM activity of PHF8 toward H3K9me2 and promoting rDNA transcription and S-phase progression of 293T, Hela, or U2OS cells^12^. Because of the transcriptionally repressive nature of chronically active PP2Ac that dephosphorylates Ser843 that is crucial for PHF8 KDM activity, we postulate that the KDM activity of PHF8 could be modulated in the phosphorylation-dependent way (not necessarily by CDK2/cyclin E kinase). The detailed mechanism underlying PHF8 activation is under investigation and will be reported elsewhere.

Notably, the mechanism of the gene-specific control of inflammation by TLR4-induced chromatin modification was discovered previously by microarray analysis of LPS-induced differential gene expression. Unknown was exactly how different classes of genes are regulated at the epigenetic level by specific chromatin modifiers. We have now identified the LPS-induced, PHF8-dependent, T-class secretome at the more physiologically relevant protein level, thus directly and systematically extending the function of PHF8 in a broad range of biological processes and pathways.

One of the major extracellular functions of TLR-mediated innate immunity is to instruct the activation of adaptive immunity through secretion of specific signaling or messenger molecules, such as selectins, chemokines, cytokines, and chemokine receptors^1^; specifically, selectins recruit leukocytes, chemokines activate leukocytes activating integrins, and the integrins regulate adhesion to the vascular endothelium^39^. By profiling the LPS-induced PHF8-dependent secretome, we have characterized novel extracellular functions controlled by PHF8 as a broad regulator of the innate immunity-dependent activation of adaptive immunity. This discovery agrees with a previous report suggesting a possible adaptive immunity function of PHF8 as a transcriptional activator of hairy and enhancer of split-1 (HES1), Deltex 1 (DTX1), IL7R, NOTCH3 for regulating T cell differentiation^55^. Specifically, our secretome data indicate that PHF8 is responsible for activation of adaptive immunity by regulating the secretion of multiple chemoattractants as well as multiple products of MHC genes that play crucial roles in antigen presentation to T cell^30^. Our combined results indicate that PHF8 is an epigenetic regulator of a broad range of secreted proteins that are crucial for leukocyte/T cell activation and proliferation.

In summary, our combined strategy is a systematic, efficient, and precise way to comprehensively characterize the global impact of PHF8 on multiple layers of epigenetic regulation. To extend our new findings about the pro-inflammatory nature of PHF8, we have conducted a thorough, in-depth proteomic and secretome investigation to explore novel regulatory functions of PHF8. At the core of the transcriptional regulation, PHF8 regulates multiple LPS-induced extracellular biological processes, including the activation of pro-inflammatory cytokines, antigen presentation, MHC expression, expression/secretion of adhesion molecules, and activation of adaptive immunity. System-wide, our pathway/network findings based on the LPS-inducible PHF8-dependent secretome illustrate that acutely active PHF8 regulates the products of the innate immune response that instruct the activation of adaptive immunity, and, therefore, PHF8 is the primary epigenetic regulator bridging innate immunity and adaptive immunity. Under a chronic inflammatory condition, PHF8 is deactivated and is substituted by chronically active G9a when the same sets of the genes are suppressed/silenced. Our studies of the interchangeable chromatin regulators under different inflammatory conditions may mechanistically derive biomarkers of immunopathology associated with the extremes of deregulated inflammation.

## Methods

### Reagents

Lipopolysaccharide (LPS), trypsin, protease inhibitor cocktails, and phosphatase inhibitor cocktails were purchased from Invitrogen, Promega, Sigma-Aldrich (St. Louis, MO), and Pierce, respectively. All culture media and fetal bovine serum (FBS) were obtained from GIBCO and dialyzed FBS was purchased from Invitrogen. All stable isotope-enriched amino acids, including ^12^C_6_-arginine, ^13^C_6_-arginine, and ^13^C_6_^15^N_4_-arginine, ^12^C_6_-lysine, ^13^C_6_-lysine and ^13^C_6_^15^N_2_- lysine, were obtained from Cambridge Isotope and Sigma-Aldrich. All chemicals were sequence- or HPLC-grade unless specifically indicated. Antibodies were purchased from Santa Cruz Biotechnology (Lmnb1 and p65), Abcam(γ-tubulin, histone H3, H3K9me2, H3K9me1, and H3K27me2), Millipore (PP2Ac clone 1D6), Cell Signaling (p-IκBa S32, IκBa, p-p65 NFκB S536, and p65) and Bethyl Labs (PHF8). Bacterial clones for shRNA against PHF8 or GFP were purchased from Sigma-Aldrich. P14 transgenic mouse, in which the CD8^+^ T cell encodes a T cell receptor that is specific for a peptide (P14, GP33-41) from the lymphocytic choriomeningitis virus (LCMV) presented by the MHC class I molecule H2-Db, is kindly provided by Jason Whitmire. CD8 microbeads and MACS separation columns were purchased from Miltenyi Biotec. GP33-41 peptide was obtained from Anaspec. Mitomycin C was from Sigma-Aldrich.

### Transfection and Stable Knockdown Cell Lines

The lentiviral plasmids pLKO.1 expressing shRNA-PP2Ac (targeting sequences CCAGATACAAATTACCTGTT and CGACGAGTGTTTAAGGAAATA), and shRNA-PHF8 (targeting sequences CGACCCTGATAATAAGACCAA for human and GCAAGATGAAACTCGGTGATT for mouse) were purchased from Sigma. A pLKO.1 empty vector (EV) with shRNA-GFP was used as the wild-type control (shCON). To produce virus, pLKO.1-shRNA plasmids were co-transfected into 293T cells with ViraPowerMix (Invitrogen) by jetPRIME^TM^ *in vitro* transfection reagent (Polyplus). Pseudo-virus was collected 48 h post-transfection and used to transduce RAW 264.7 cells by spinoculation. After 48 h, 8 μg/mL puromycin was added to select puromycin-resistant clones. Stable clones were maintained in medium containing 4 μg/mL puromycin. The knockdown efficiency was monitored with immunoblotting and qPCR.

### Quantitative Phosphoproteome Analysis Using AACT

The RAW 264.7 cells stably expressing shRNA for pLKO.1 empty vector were cultured in “L” medium and remained unstimulated (WT-N). This control cell line was also cultured in double-tagged “M” medium and was stimulated with 0.1 mg/mL LPS for 24 h then challenged with 1.0 mg/mL LPS for 15 min (WT-TL). RAW cells expressing shRNA for PP2Ac in double-tagged “H” medium were also stimulated with 0.1 mg/mL LPS for 24 h followed by a second challenge with 1.0 mg/mL LPS for 15 min (PP2AKD-TL). Cells were harvested and lysed in lysis buffer (8 M urea, 50 mM Tris pH 8.0, 75 mM NaCl, 1 mM MgCl_2_, 500 units Benzonase, and protease-phosphatase inhibitor cocktails). 5 mg of each lysate was mixed and reduced with DTT followed by alkylation with iodoacetamide (IAA). Proteins were digested first with endoproteinase Lys-C (Wako USA). The solution was diluted 4-fold with 25 mM Tris pH 8.0, 1 mM CaCl_2_ and further digested with trypsin (Promega). The digestion was stopped by TFA (0.4% final). Desalting was achieved on a Sep-Pak Light C18 cartridge (Waters). Desalted peptides were freeze-dried, resuspended in 30% acetonitrile (ACN)/0.1% TFA, and loaded on a 1 mL Resource 15S (GE Healthcare) column for strong cation exchange chromatography (SCX) with a linear gradient from 5 mM to 100 mM KCl in 30% ACN, 5 mM KH_2_PO_4_, 0.1% TFA. Negatively charged peptides were eluted with high salt buffer (350 mM KCl in 30% ACN, 5 mM KH_2_PO_4_, 0.1% TFA). The phospho-peptides were enriched directly in SCX fractions^9^. Briefly, 1–5 mg of 5 mm Titansphere beads (GL Sciences) suspended in 80% ACN/1% TFA were added to each fraction and incubated for 30 min at room temperature (RT). The beads were collected by centrifugation, washed three times with 150 mL 60% ACN/1% TFA, and transferred on to the top of a C8 disc (Empore) placed in a 200 μL pipette-tip. Bound phospho-peptides were eluted with 15% NH_4_OH/40% ACN, dried, and desalted on a StageTip containing a 4 × 1 mm C18 extraction disk (3M).

### Canonical Pathway Analysis

Biological processes and molecular functions of the proteins identified as potential PP2Ac targets were categorized by Ingenuity Pathway Analysis (IPA, QIAGEN Redwood City, http://www.qiagen.com/ingenuity). To focus on the phospho-peptides that are regulated downstream PP2Ac, the dataset was trimmed to include only the peptides that showed an increase in phosphorylation in PP2AcKD-TL compared to WT-TL. The canonical pathways were similarly ordered according to the ratio of phospho-peptides that showed an increase in PP2AcKD-TL compared to WT-TL.

### RNA Preparation and Real-Time PCR

Stable cell lines were seeded into 12-well cell culture dishes and treated with LPS for indicated times. Total RNA was isolated using illustra RNAspin Mini Kit (GE Healthcare Life Sciences). First-strand cDNA was synthesized by M-MLV reverse transcriptase (Promega) and diluted 5-fold for qPCR. Real-time PCR was performed using Maxima SYBR Green/ROX (Thermo Scientific). All measurements were normalized against GAPDH as the internal control using 2^−ΔΔCt^ method. The sequences of primers are included in Supplementary Table 7.

### Immunoblotting Analysis

Stable cell lines from each N, NL, TL condition were harvested and lysed with buffer containing 0.5% NP-40, 10 mM Tris pH 7.5, 150 mM NaCl, 0.4 mM EDTA, 2 mM Na_3_PO_4_, 1x phosphatase inhibitor cocktail, 1x protease inhibitor cocktail.

### Nuclear and Cytoplasmic Fractionation

The nuclear and cytoplasmic proteins were fractionated with a CelLytic NuCLEAR Extraction Kit (Sigma) according to the manufacturer’s instructions. Briefly, adherent cells were washed three times with PBS, scraped, and centrifuged for 5 min at 400 × *g*. The cell pellets were resuspended in hypotonic lysis buffer (10 mM Hepes pH 7.9, 1.5 mM MgCl_2_, and 10 mM KCl, 10 mM DTT, protease inhibitors, and phosphatase inhibitors), incubated on ice for 15 min to swell the cells, and lysed gently using IGEPAL CA-630 (NP40) (0.6% final). The lysates were vortexed and centrifuged immediately for 30 sec at 10,000 × *g*. The supernatants (cytoplasmic fraction) were transferred to new tubes. The nuclear pellet was lysed with buffer containing 50 mM Tris-HCl, pH 7.5; 150 mM NaCl, 0.5% Triton-X 100, 1X phosphatase inhibitor cocktail, 1X protease inhibitor cocktail followed by sonication at level 3 (5 sec on, 5 sec off; twice) for the removal of DNA from chromatin.

### Secretome Analysis

The secretome analysis method was performed as described^8^. Briefly, RAW 264.7 cells were cultured in regular DMEM medium with 10% FBS, transfected with shCON or shPHF8. 24 h prior to the LPS challenge, cells were washed with and cultured in serum-free DMEM containing 1 mM sodium pyruvate and 10 mM L-glutamine with no phenol red. Cells were either left unstimulated (0) or subjected to 1 mg/mL LPS challenge for indicated hours (2, 4, 8, 24). The secreted proteins were harvested and prepared as previously described^15^. Briefly, the secretome-containing culture medium was centrifuged at 400 × *g* for 5 min for removal of dead cell debris. The supernatant was collected with an 18-gauge needle, syringe-filtered with 0.2 μm 13 mm diameter PTFE filters (VWR International), transferred into fresh tubes, and kept at −80 °C until further processed. After removal of the extracellular media, attached cells were lysed with 1 X SDS-loading buffer to perform immunoblots to confirm the inflammation phenotype. Before MS analysis, we thawed the secretome-containing medium and diluted in 4 X lysis buffer (8M urea, 40 mM HEPES pH 7.9) to bring the final urea concentration to 2 M. The lysate was then sonicated at level 3 for 5 seconds, reduced with DTT (10 mM final) for 40 min at RT, and alkylated with IAA (50 mM final) for 40 min in the dark at RT. Alkylation was quenched with freshly prepared thiourea (100 mM final). CaCl_2_ was added (1 mM final) before trypsin digestion overnight at RT. The digestion was quenched with TFA (0.5% final). Peptides were dried and resuspended at 0.1% Formic acid (FA) for MS/MS. We used reversed phase LC-MS/MS using a Proxeon 1000 nano LC system coupled to an LTQ Orbitrap Velos mass spectrometer (Thermo Scientific, San Jose, CA). The peptides were trapped using a 3 cm long 100 □m i.d. C18 column at 5 μL/min liquid flow that was diverted from the analytical column via a vent valve while elution was performed by switching the valve to make the trap column in-line with a 15 cm long, 75 μm i.d., 3.5 μm, 300 Å particle C18 analytical column. The digested peptides were separated with a linear gradient of 2–35% buffer B over 240 min at a 300 nL/min flow rate using 0.1% FA (buffer A) and ACN with 0.1% FA (buffer B). Each secretome sample had two biological replicates, which were subjected to 3 single-shot independent LC-MS runs for global peptide analysis. Database search, peptide identification, and LFQ were performed as previously described^8^.

### P14 CD8^+^ T cell proliferation and activation assays

For T cell proliferation assay, CD8^+^ T cells from P14 transgenic mouse were first isolated with CD8a microbeads according to manufactory’s instruction. Isolated CD8^+^ T cells were re-suspended in 1 mL 1640 medium and either labeled with 1 mL of the 10 μM Carboxyfluorescein diacetate succinimidyl ester (CFSE) for 8 min at RT for proliferation assay or kept unlabeled for activation assay. Cells were washed with 10 mL 1640 medium containing 10% FBS. Meanwhile WT (shCON) and PHF8-KD (shPHF8) RAW cells were seeded in 6-well plates and treated with 1 μg/mL LPS for 0, 8, and 24 h followed by 50 μg/mL Mitomycin C treatment for 30 min at 37 °C. Cells were washed with 1 mL PBS twice and then labeled with 0.4 mL of the 30 μg/mL GP33-41 peptide in PBS for 30 min at 37 °C. The unattached peptides were washed with PBS. 0.1 × 10^6^ P14 T cells are co-cultured with 2 × 10^5^ WT or PHF8-KD RAW cells in 96-well plates in RPMI medium containing 50 U/mL mIL-2. The proliferation and activation of CD8^+^ T cells were assessed by flow-cytometry at day 6.

## Additional Information

**How to cite this article**: Erdoğan, Ö. *et al*. Proteomic dissection of LPS-inducible, PHF8-dependent secretome reveals novel roles of PHF8 in TLR4-induced acute inflammation and T cell proliferation. *Sci. Rep*. **6**, 24833; doi: 10.1038/srep24833 (2016).

## Supplementary Material

Supplementary Information

Supplementary Dataset 1

## Figures and Tables

**Table 1:** List of cytokines and chemokines identified in the LPS-inducible secretome in comparison with previous BMDM secretome studies^8^,^15^.

Cytokine/Chemokine	PHF8-dependentsecretion	Identified inMeissner *et al*.^15^	Identified inLiu *et al*.^8^
Aimp1	✓		✓
Ccl2	✓	✓	✓
Ccl22		✓	
Ccl3		✓	✓
Ccl4		✓	✓
Ccl5		✓	✓
Ccl7	✓	✓	
Ccl9	✓	✓	✓
Csf3		✓	
Cxcl10	✓	✓	✓
Cxcl16	✓	✓	
Cxcl2	✓	✓	✓
Ebi3	✓	✓	
Ifnb1		✓	
Il27	✓	✓	
Il6		✓	✓
Lif	✓		
Mif			
Osm			
Spp1		✓	
