# Supplementary material for: Proteomic dissection of LPS-inducible, PHF8-dependent secretome reveals novel roles of PHF8 in TLR4-induced acute inflammation and T cell proliferation
Source: Sci Rep. 2016 Apr 26;6:24833. doi: 10.1038/srep24833 (PMC4845005; doi:10.1038/srep24833)
Supplement: Supplementary Information [file srep24833-s1.pdf]

Proteomic dissection of LPS-inducible, PHF8-dependent secretome reveals novel roles of PHF8 in TLR4-induced acute inflammation and T cell proliferation

Özgün Erdoğan, Ling Xie, Li Wang, Bing Wu, Qing Kong, Yisong Wan, and Xian Chen

S1a

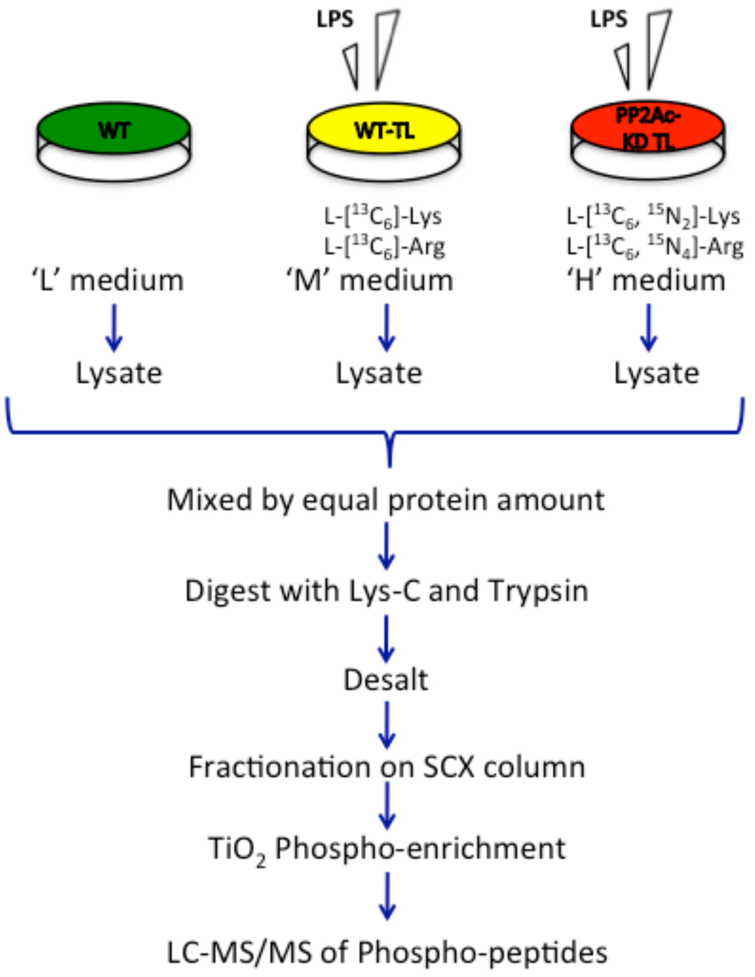

S1b

DNA Methylation and Transcriptional Repression

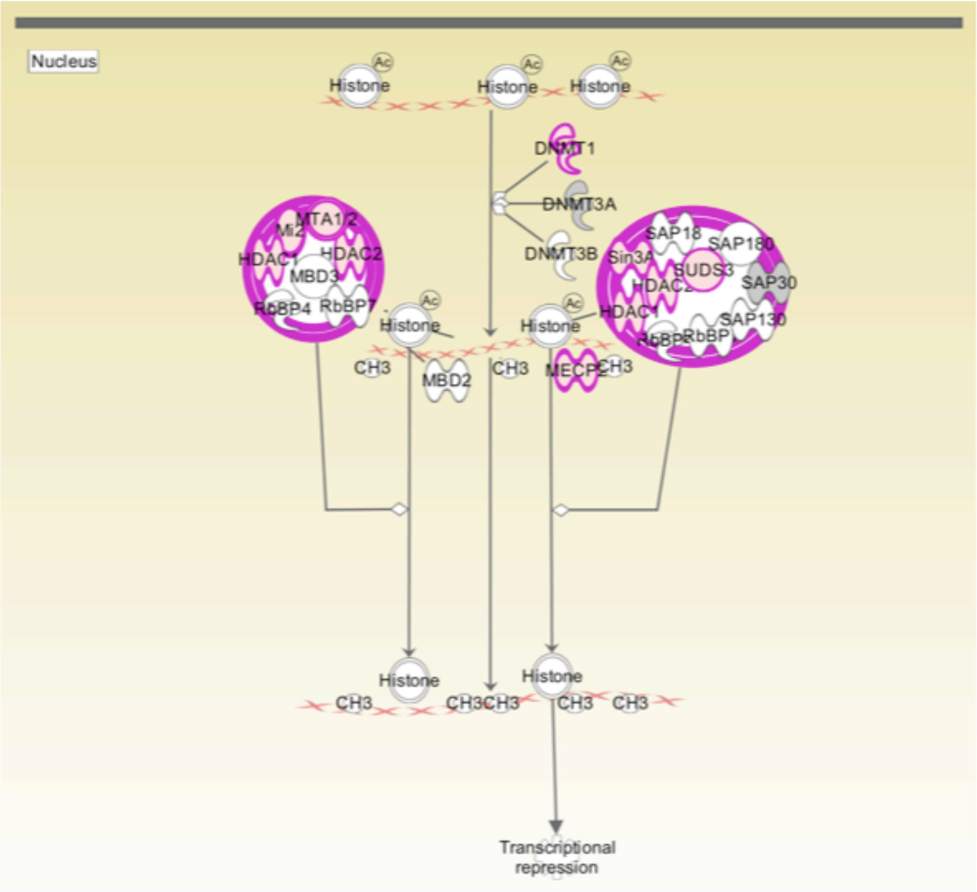

PI3K/AKT Signaling

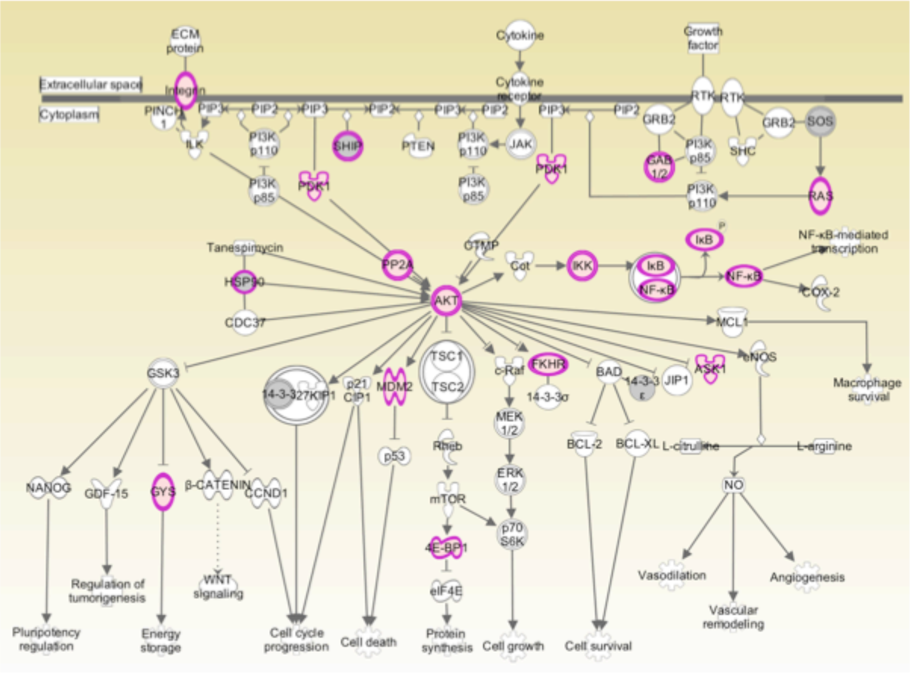

Rac Signaling

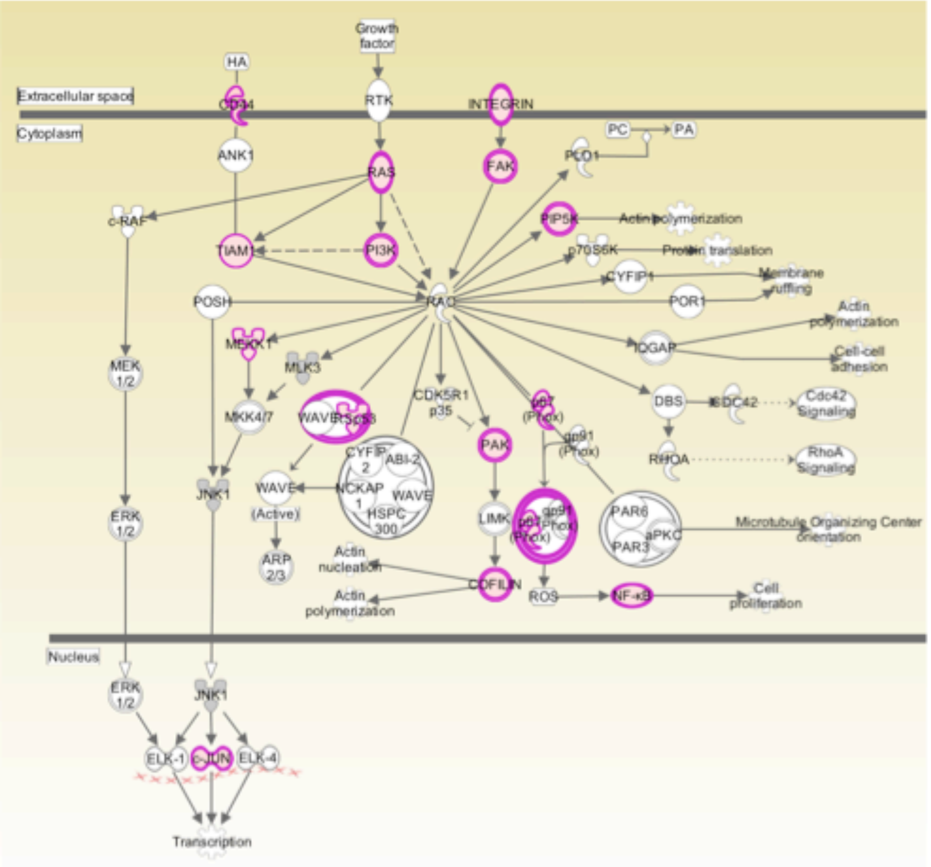

Telomerase Signaling

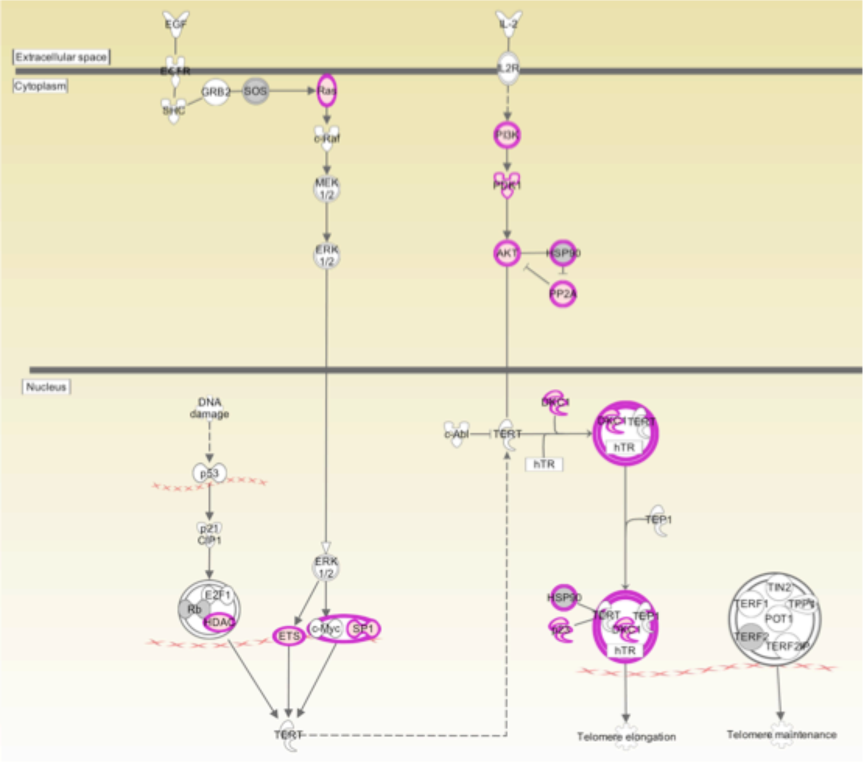

ERK/MAPK Pathway

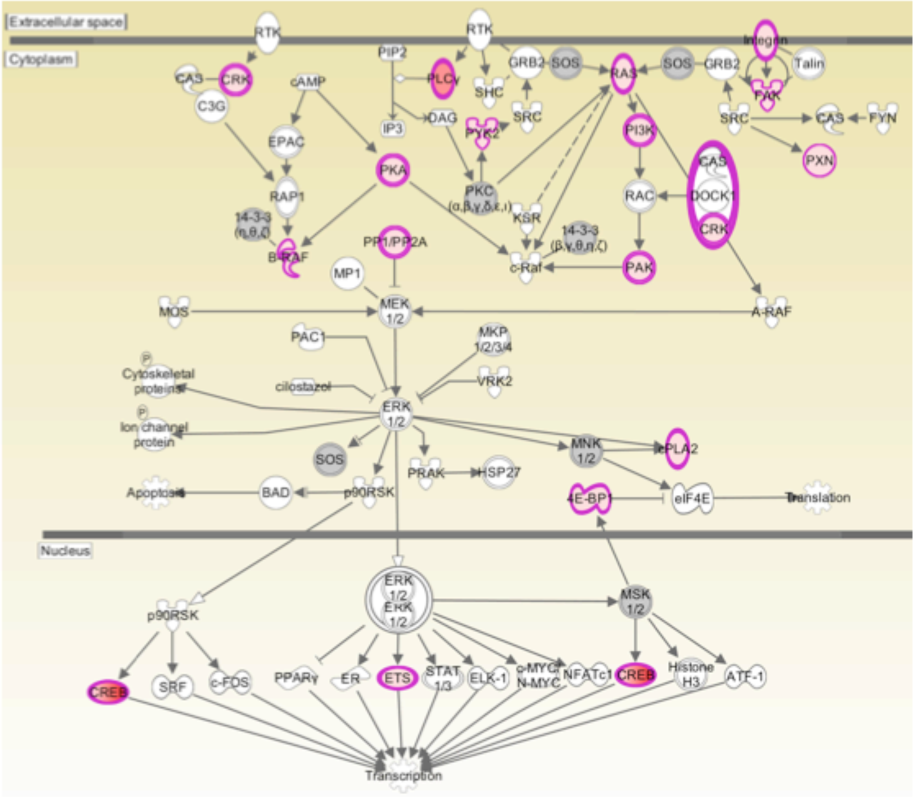

HGF Signaling

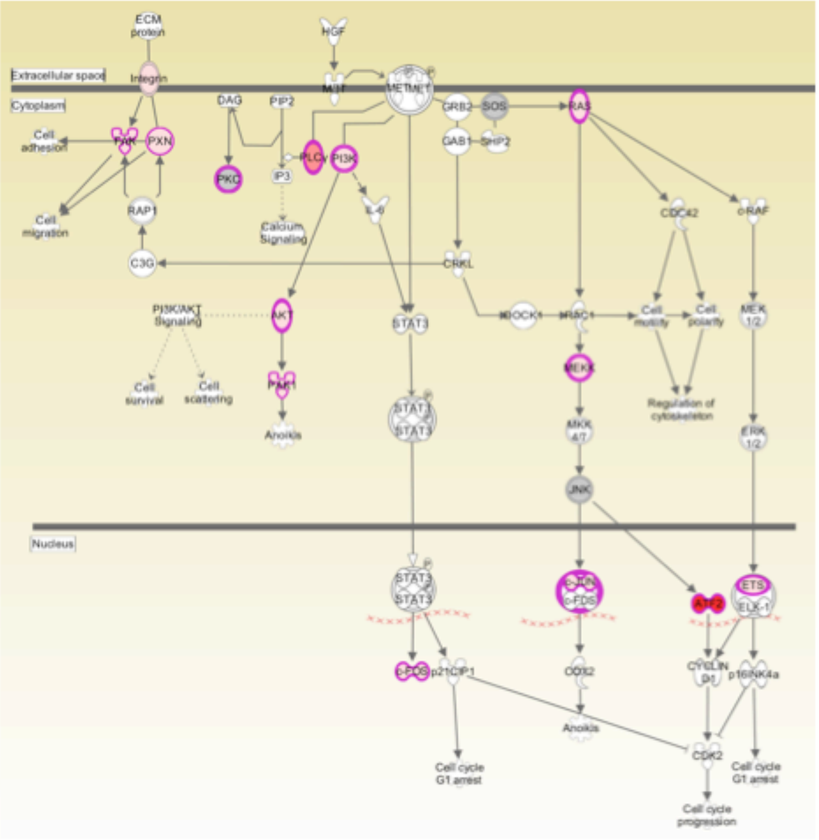

ATM Signaling

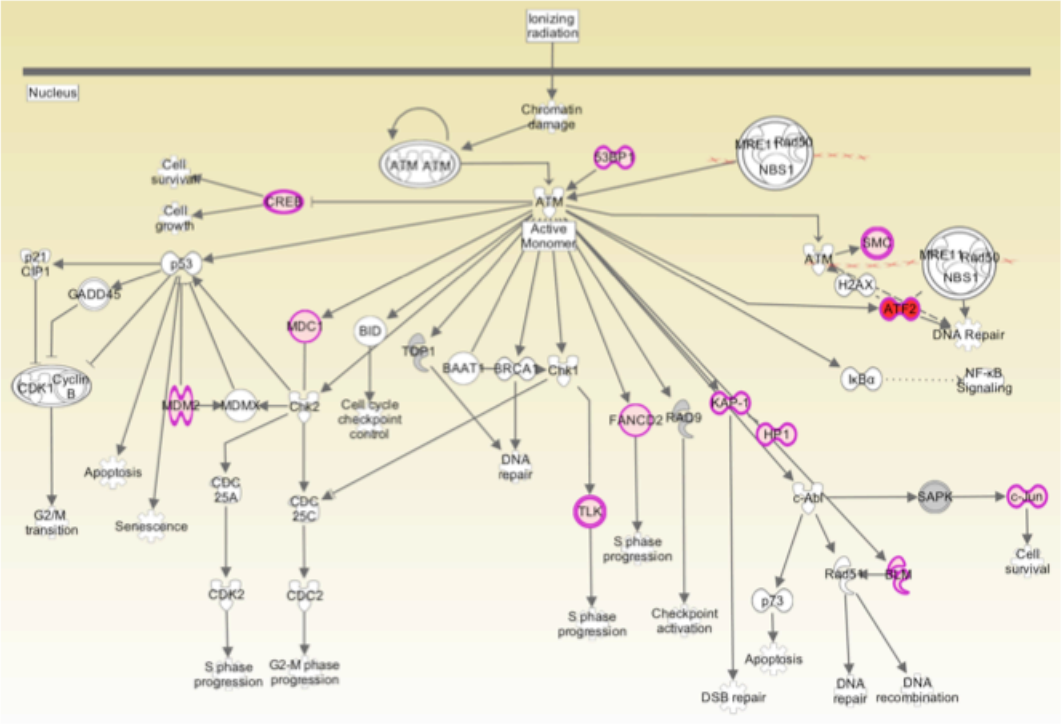

Legend

- Complex
- Enzyme
- Graphic node
- Group/Complex
- Transcription Regulator
- Other
- Function
- Relationship

Colors:

- Phosphorylation not affected by PP2Ac-KD
- Phosphorylation upregulated by PP2Ac-KD
- Potential PP2A targets

S1c

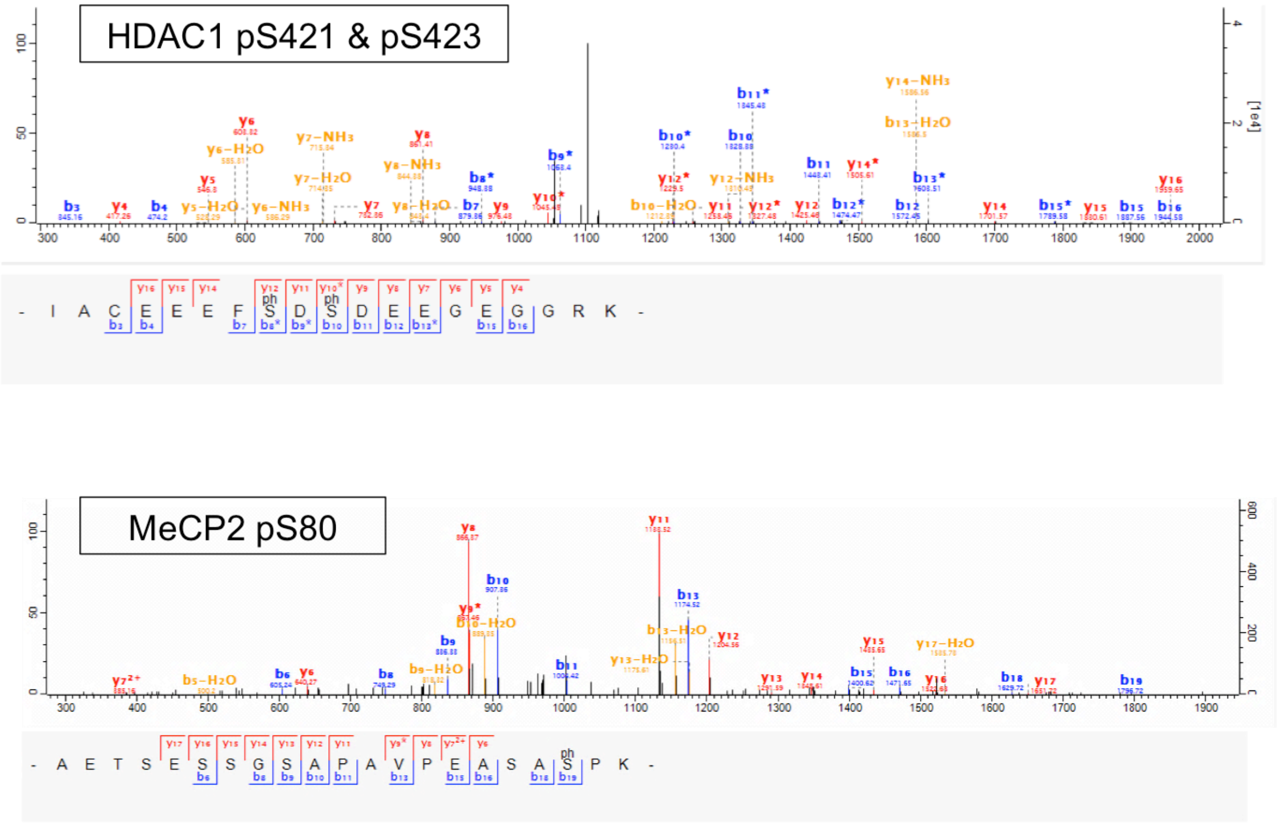
HDAC1 pS421 & pS423
MeCP2 pS80

S1d

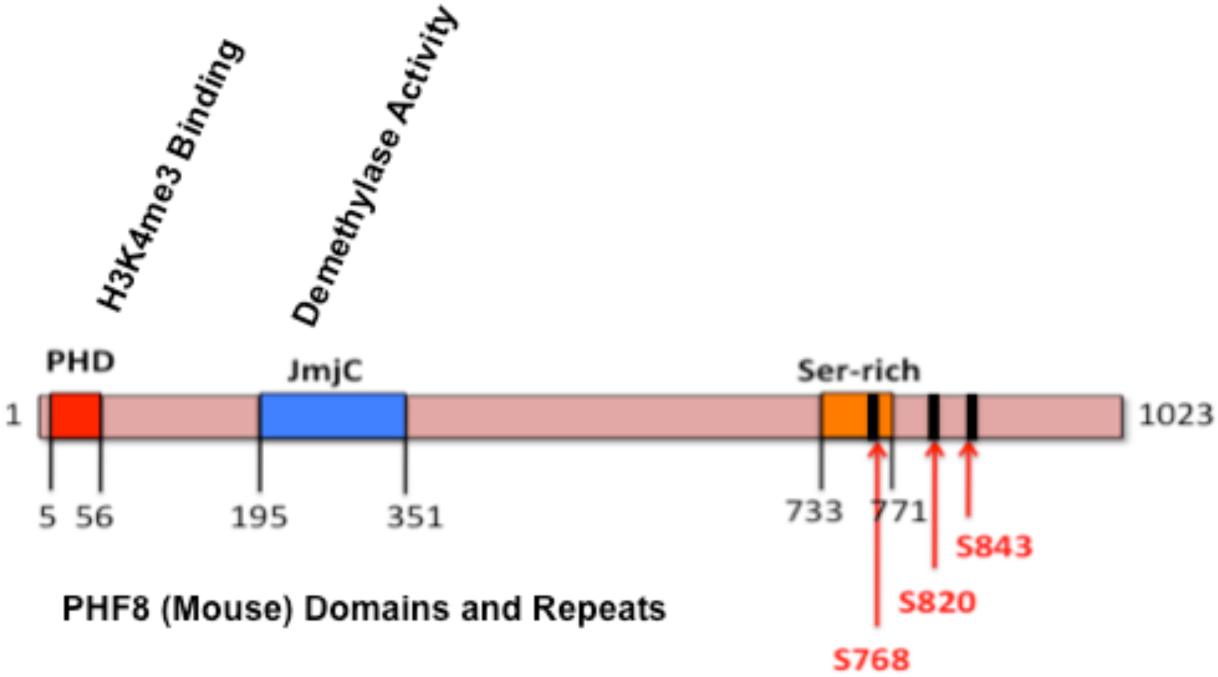
H3K4me3 Binding
Demethylase Activity
PHD
JmjC
Ser-rich
1
1023
5
56
195
351
733
771
S843
S820
S768
PHF8 (Mouse) Domains and Repeats

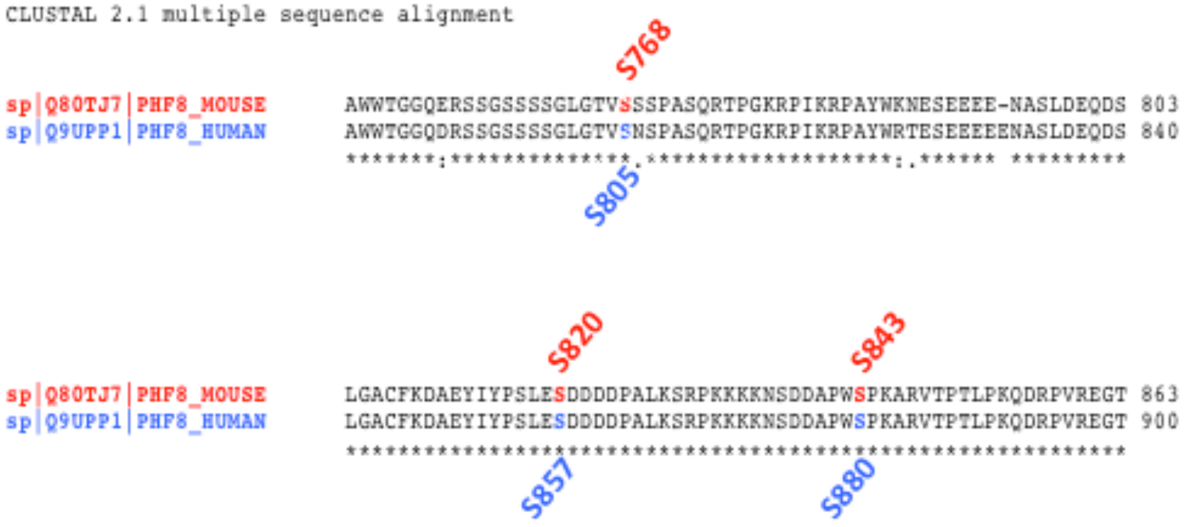

S1e

PHF8 pS768:
SSGSSSSGLGTVSpSSPASQR

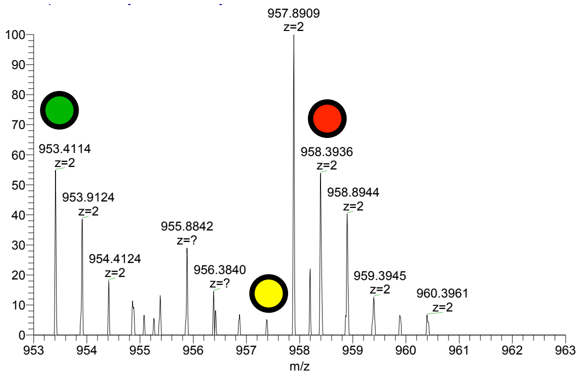

PHF8 pS820:
DAEYIYPpSLESDDDDPALK

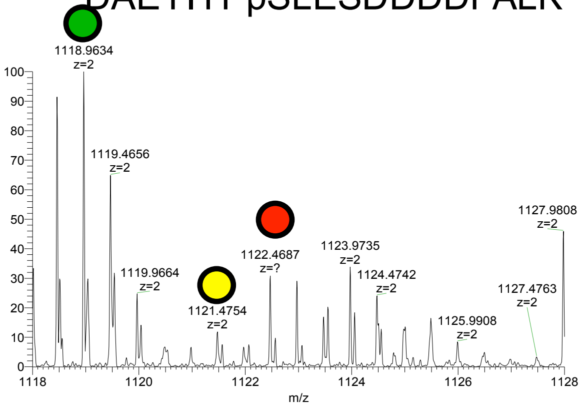

PHF8 pS843:
NSDDAPWpSPK

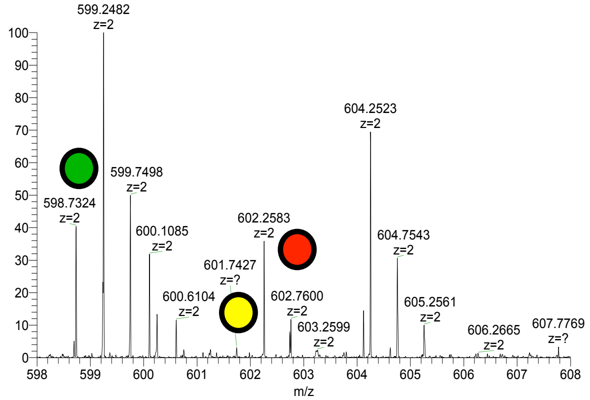

S1f

PHF8 pS768

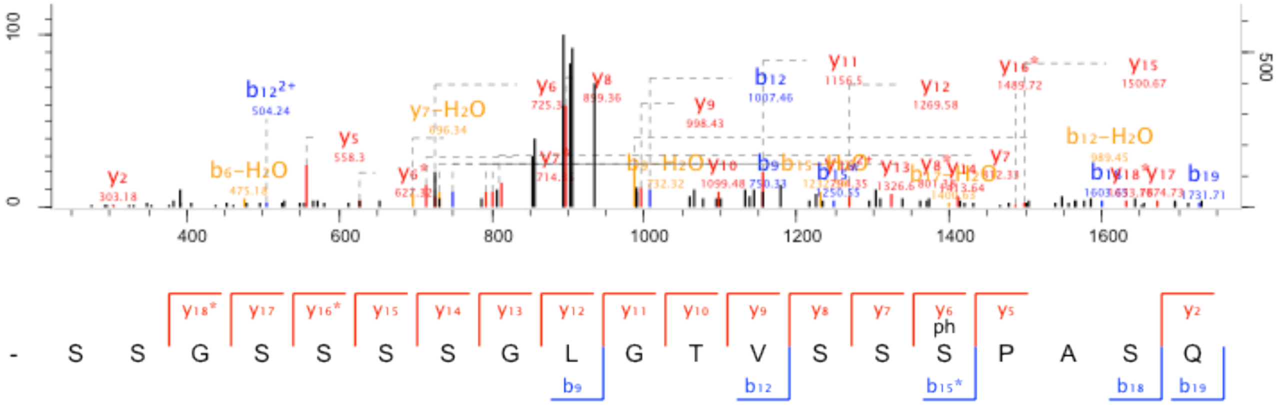

PHF8 pS820

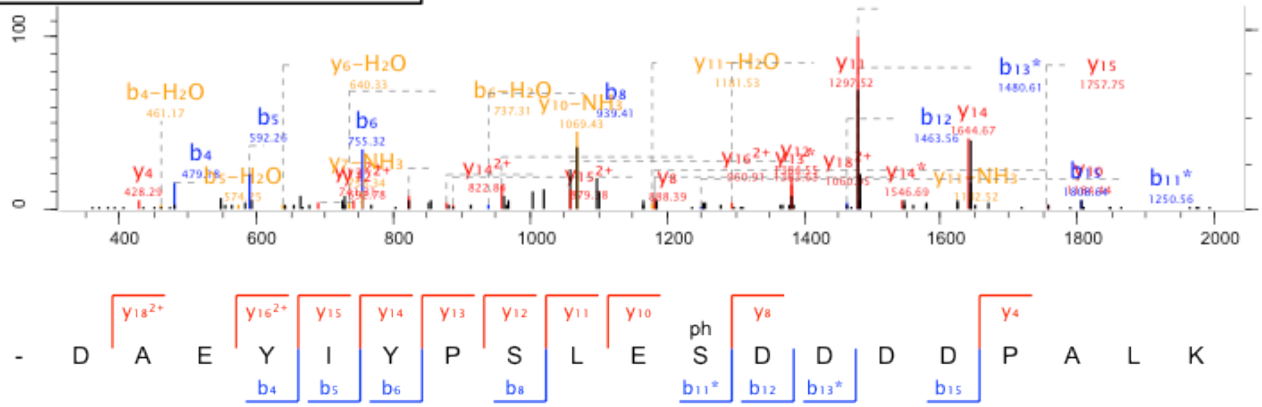

PHF8 pS843

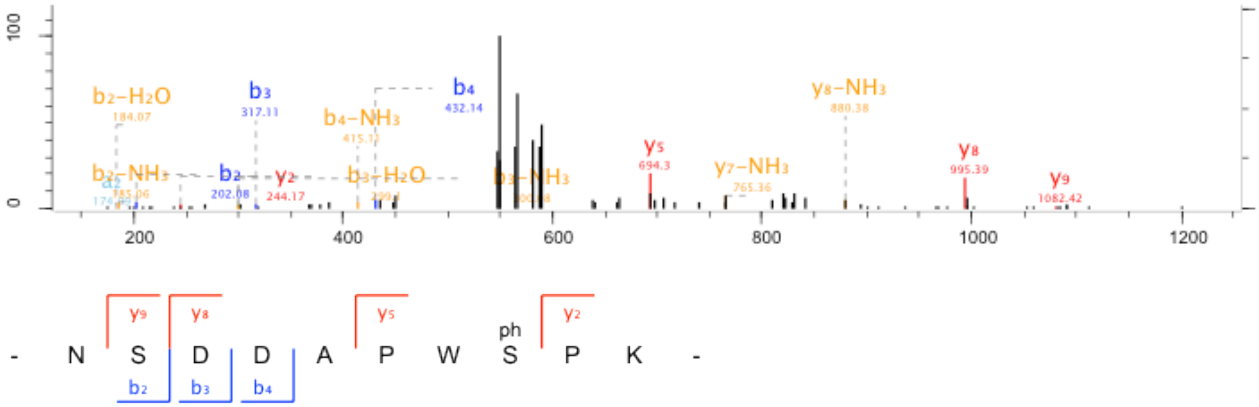

Supplementary Figure 1 Quantitative phosphoproteomics reveals pathways and proteins targeted by PP2Ac in ET. **(a)** Workflow of the phosphoproteomic analysis of PP2Ac-targeted proteins in RAW 264.7 cells via AACT labeling using WT and PP2Ac-KD RAW cells. WT (M, yellow) and PP2Ac-KD (H, red) Raw cells were treated with prolonged LPS (0.1 ug/ml) and challenged with a second stimulation with LPS (1ug/ml). These were labeled as 'WT-TL' and 'PP2Ac-KD TL', respectively. Non-stimulated WT cells (L, green) were used as cellular control and were labeled as 'WT'. Mixing equal amount of protein from each pool of cells, cell lysates were digested with Lys-C and trypsin consecutively. Following desalting and SCX fractionation, phospho-peptides were enriched with TiO2 beads for MS/MS analysis. **(b)** Network analysis via IPA showing PP2Ac-targeted canonical pathways in ET RAW 264.7 macrophages containing multiple PP2Ac-target proteins (red shade) along with known pathway components without phosphorylation level changes (grey shade). Magenta circles represent the PP2Ac-targeted proteins/complexes and shape legend is given at the bottom. **(c)** MS/MS spectra of the HDAC1 (top) and MeCP2 peptides (bottom) containing PP2Ac-target residues important for enzymatic activity including S421/S423 and S80, respectively, in ET mouse RAW 264.7 macrophages. **(d)** The domain representation of PHF8. PP2Ac-target phospho-sites of PHF8 are located in the serine-rich domain of PHF8 (top, black bars). Sequence alignment for mouse and human PHF8 between residues 847 and 863 (mouse) shows that the PP2Ac target phosphorylation sites are conserved among human and mouse (bottom). **(e)** MS spectra of the PHF8 peptides containing PP2Ac-target residues including S768, S820, and S843 in ET mouse RAW 264.7 macrophages. **(f)** MS/MS spectra for PHF8 phosphopeptides pS768, pS820, and pS843 that are PP2Ac targets in ET.

S2

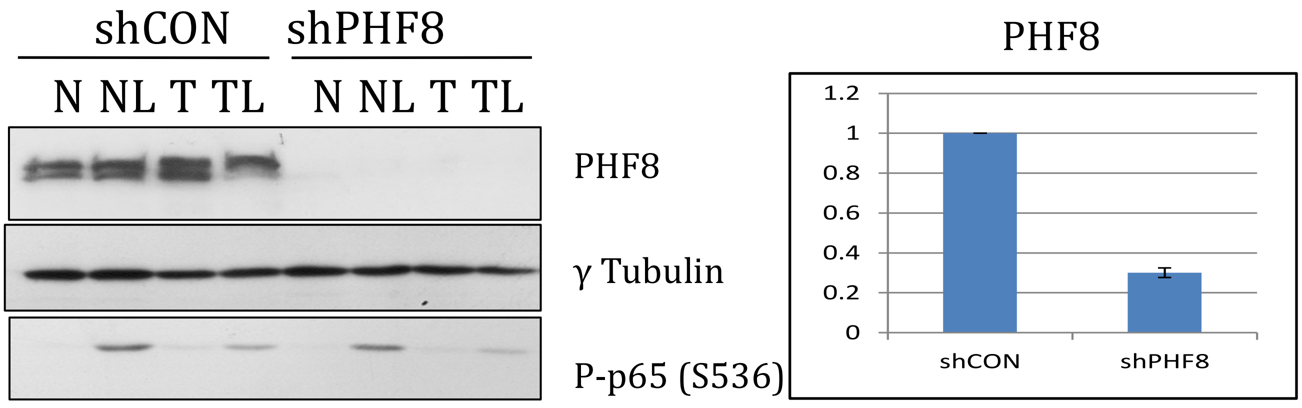

NL: 1.0 μg/mL LPS, 15 min
T: 0.1 μg/mL LPS, 24 hrs
TL: 0.1 μg/mL LPS, 24 hrs + 1.0 μg/mL LPS, 15 min

Supplementary Figure 2 Knockdown of PHF8 on mouse macrophage RAW cell line. Using shRNA against GFP and PHF8 in RAW cells, we made stable WT (shCON) and PHF8-KD (shPHF8) cell lines, respectively. Different inflammation conditions are shown as N (non-stimulated), NL (acute LPS stimulation), T (prolonged LPS stimulation), and TL (prolonged LPS stimulation with a second LPS challenge). We measured mRNA expression of PHF8 to confirm the knock-down efficiency as 70% using qPCR (right).

S3a

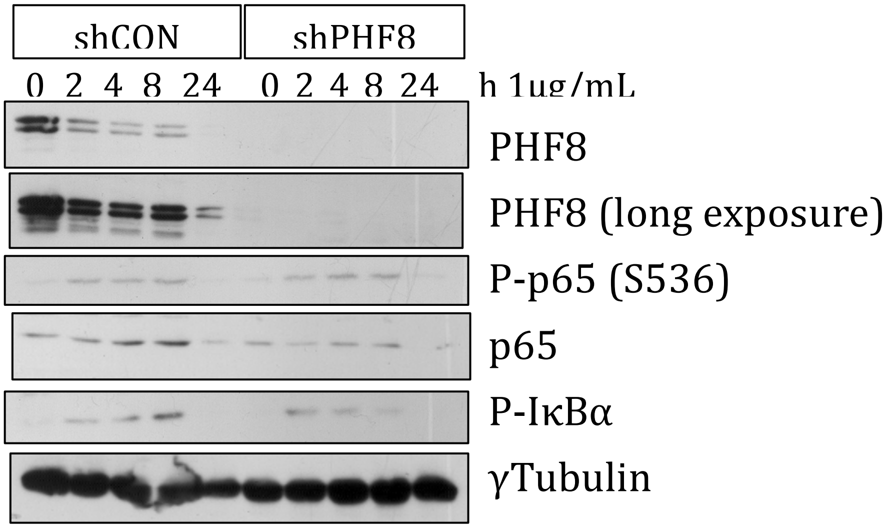

S3b

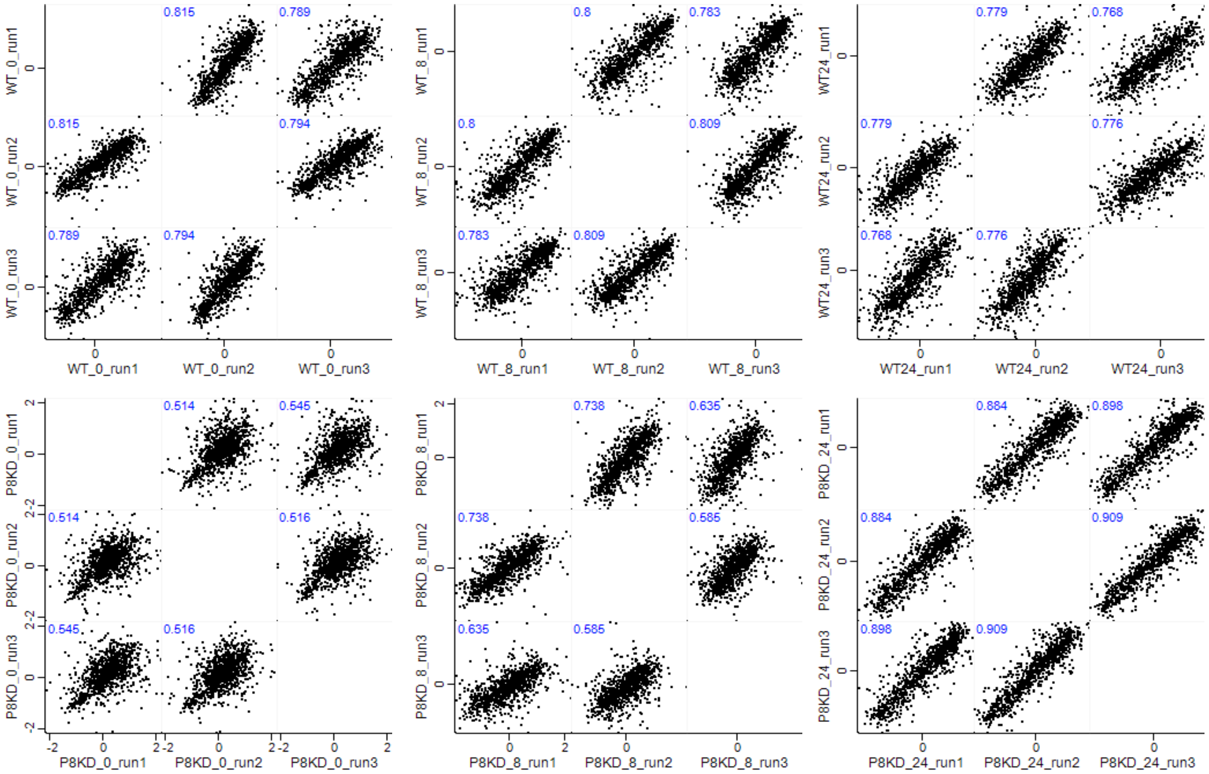

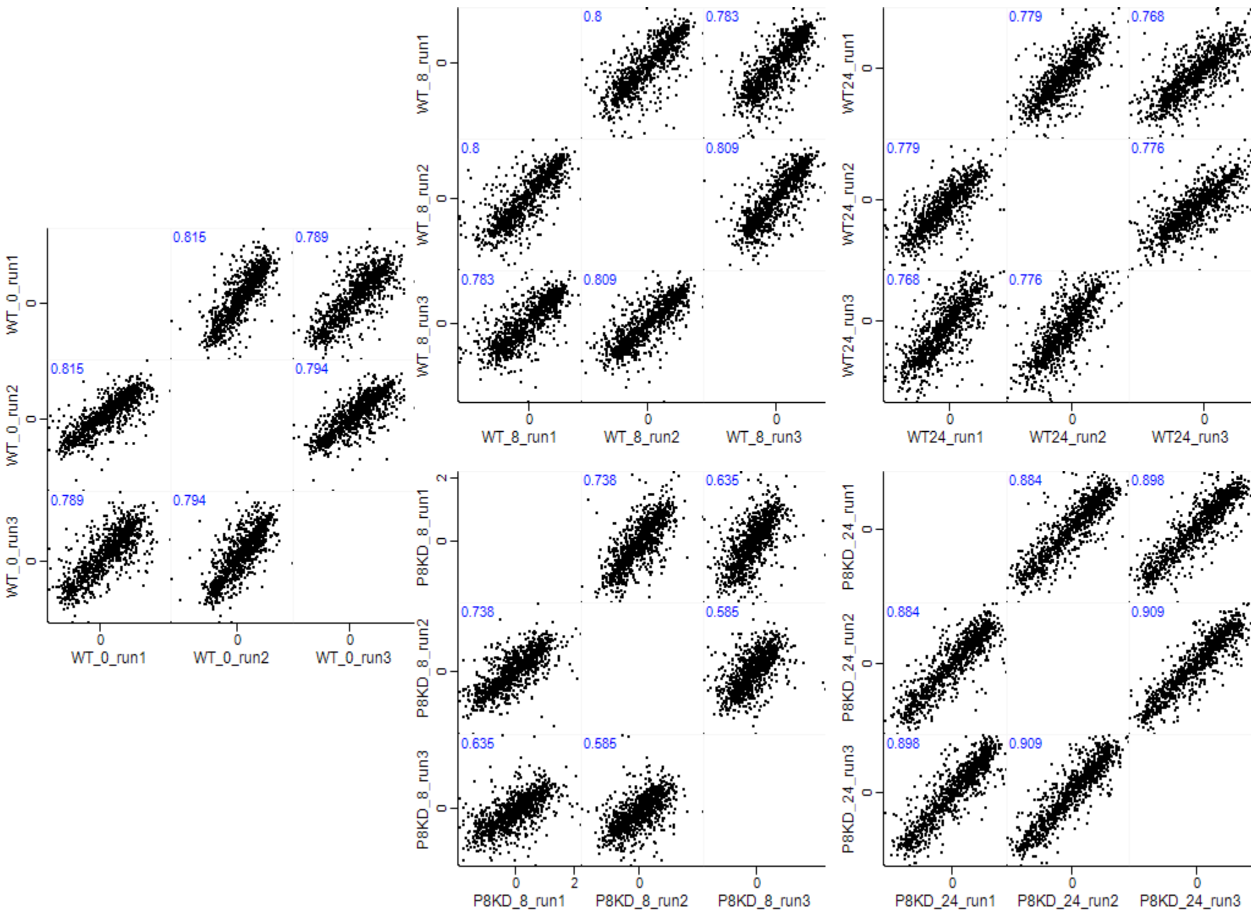
WT_0_run1
WT_0_run2
WT_0_run3
0.815
0.789
0.794
WT_8_run1
WT_8_run2
WT_8_run3
0.8
0.783
0.809
WT24_run1
WT24_run2
WT24_run3
0.779
0.768
0.776
P8KD_8_run1
P8KD_8_run2
P8KD_8_run3
0.738
0.635
0.585
P8KD_24_run1
P8KD_24_run2
P8KD_24_run3
0.884
0.898
0.909

S3c

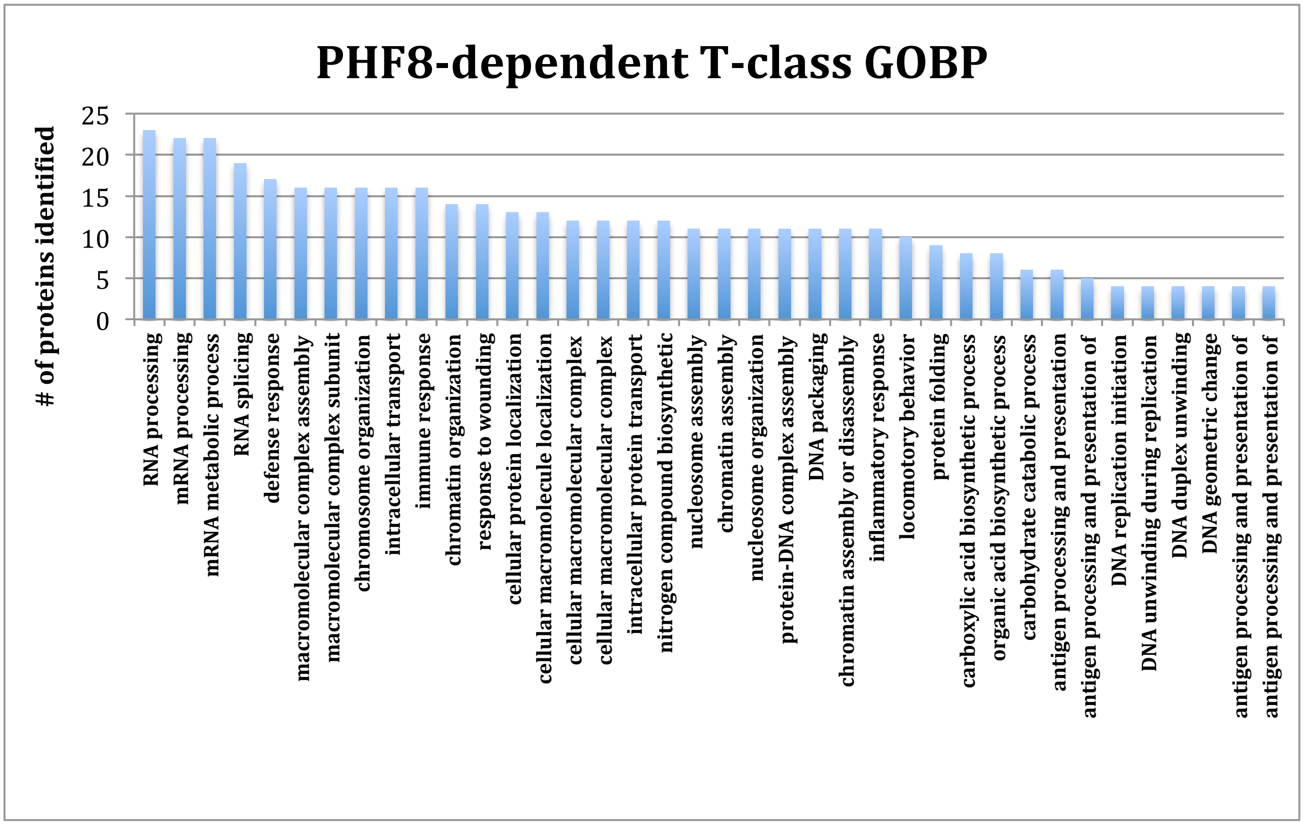
PHF8-dependent T-class GOBP
# of proteins identified
25
20
15
10
5
0
RNA processing
mRNA processing
mRNA metabolic process
RNA splicing
defense response
macromolecular complex assembly
macromolecular complex subunit
chromosome organization
intracellular transport
immune response
chromatin organization
response to wounding
cellular protein localization
cellular macromolecule localization
cellular macromolecular complex
cellular macromolecular complex
intracellular protein transport
nitrogen compound biosynthetic
nucleosome assembly
chromatin assembly
nucleosome organization
protein-DNA complex assembly
DNA packaging
chromatin assembly or disassembly
inflammatory response
locomotory behavior
protein folding
carboxylic acid biosynthetic process
organic acid biosynthetic process
carbohydrate catabolic process
antigen processing and presentation
antigen processing and presentation of
DNA replication initiation
DNA unwinding during replication
DNA duplex unwinding
DNA geometric change
antigen processing and presentation of
antigen processing and presentation of

3d

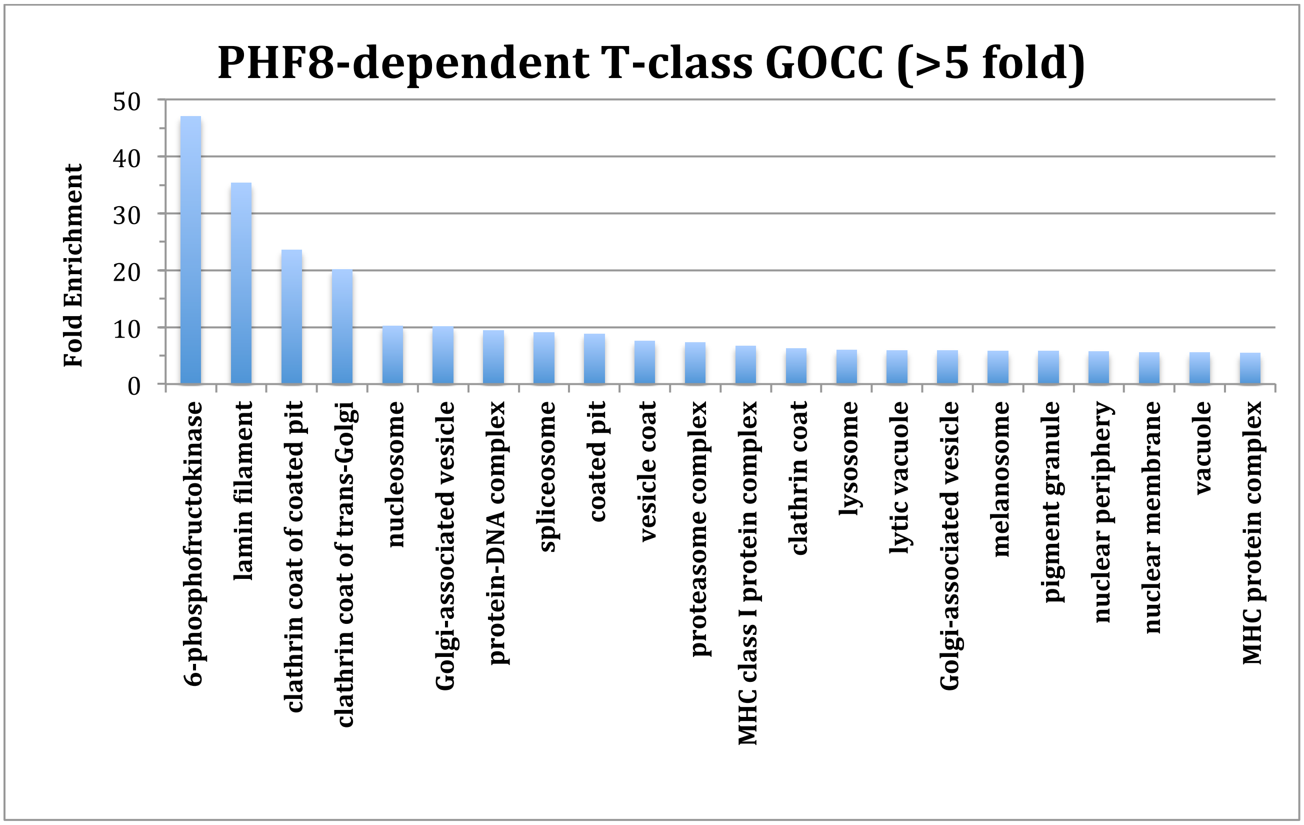
PHF8-dependent T-class GOCC (>5 fold)
Fold Enrichment
50
40
30
20
10
0
6-phosphofructokinase
lamin filament
clathrin coat of coated pit
clathrin coat of trans-Golgi
nucleosome
Golgi-associated vesicle
protein-DNA complex
spliceosome
coated pit
vesicle coat
proteasome complex
MHC class I protein complex
clathrin coat
lysosome
lytic vacuole
Golgi-associated vesicle
melanosome
pigment granule
nuclear periphery
nuclear membrane
vacuole
MHC protein complex

3e

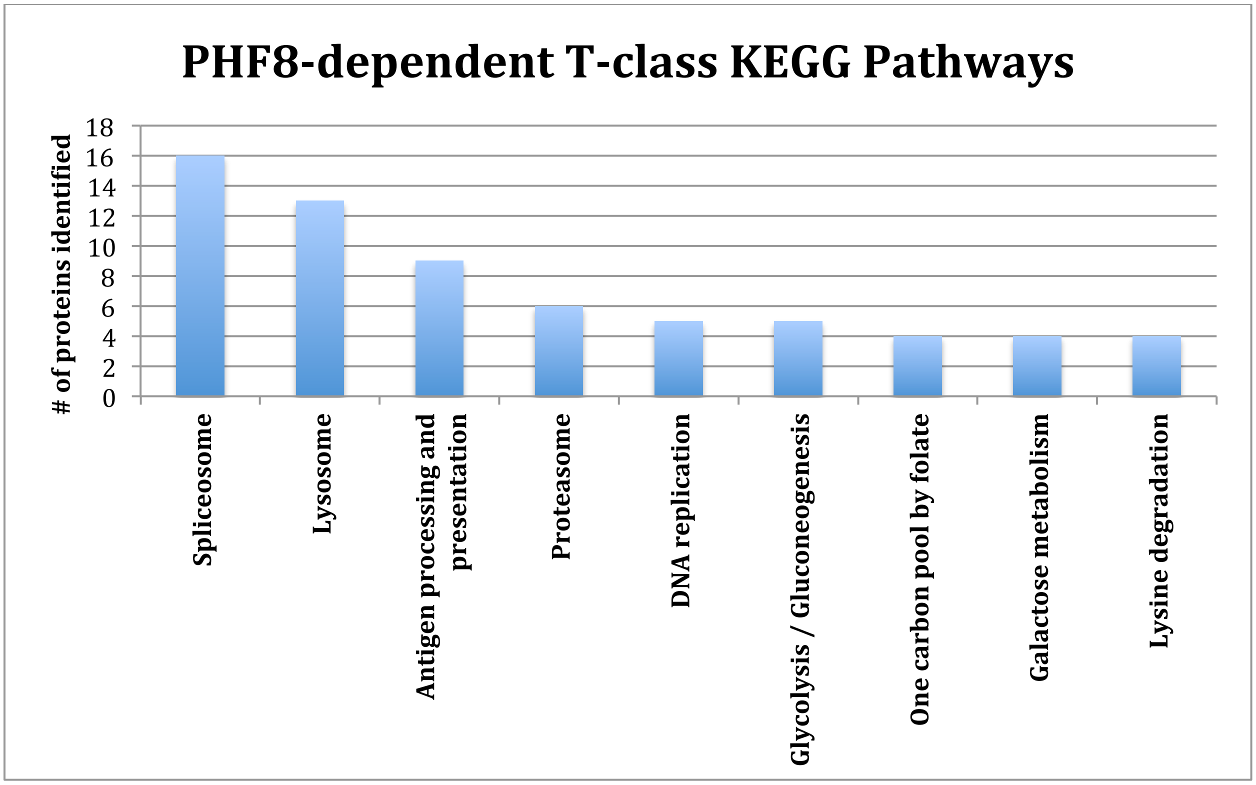

Supplementary Figure 3 Label-free quantitative proteomic analysis of secretome from WT and PHF8-KD cells reveals a novel role of PHF8 as a regulator of adaptive immunity. **(a)** Immunoblot showing the inflammation phenotype of the cells on the plates after the removal of secretome media. **(b)** Scatter plots showing Pearson correlation of the replicates of WT (top) and PHF8KD (bottom) secretome with time points indicated 0 (left), 8 (middle) and 24 h (right). **(c)** Enrichment of the GO Biological Processes (GOBP) of PHF8-dependent T-class secretome showing the number of proteins identified in each BP. **(d)** Enrichment of the GO Cellular Components (GOCC) of PHF8-dependent T-class secretome showing fold-enrichment. **(e)** Enrichment of the KEGG Pathways of PHF8-dependent T-class secretome showing the number of proteins identified in each pathway.

4a

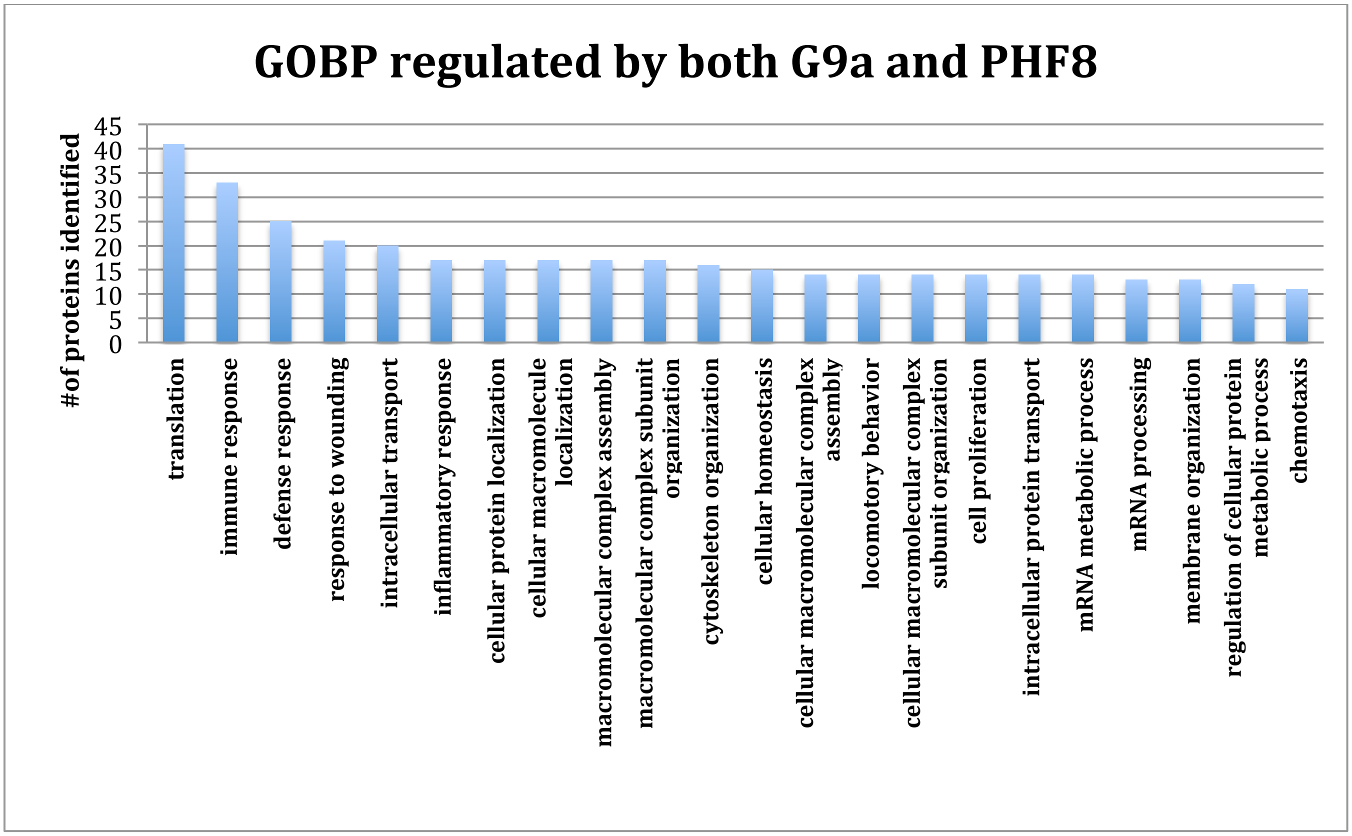
GOBP regulated by both G9a and PHF8
#of proteins identified
45
40
35
30
25
20
15
10
5
0
translation
immune response
defense response
response to wounding
intracellular transport
inflammatory response
cellular protein localization
cellular macromolecule localization
macromolecular complex assembly
macromolecular complex subunit organization
cytoskeleton organization
cellular homeostasis
cellular macromolecular complex assembly
locomotory behavior
cellular macromolecular complex subunit organization
cell proliferation
intracellular protein transport
mRNA metabolic process
mRNA processing
membrane organization
regulation of cellular protein metabolic process
chemotaxis

4b

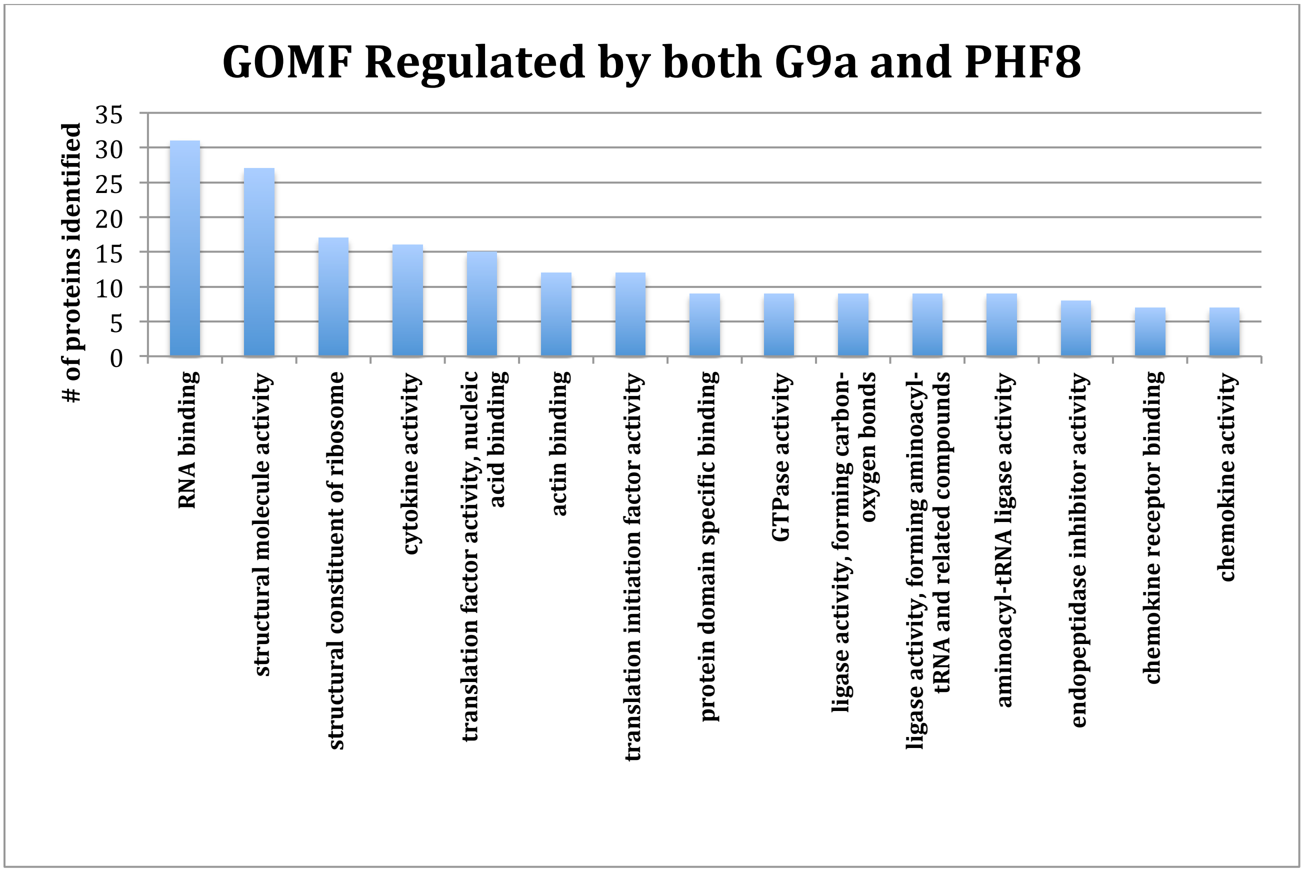
GOMF Regulated by both G9a and PHF8
# of proteins identified
35
30
25
20
15
10
5
0
RNA binding
structural molecule activity
structural constituent of ribosome
cytokine activity
translation factor activity, nucleic acid binding
actin binding
translation initiation factor activity
protein domain specific binding
GTPase activity
ligase activity, forming carbon-oxygen bonds
ligase activity, forming aminoacyl-tRNA and related compounds
aminoacyl-tRNA ligase activity
endopeptidase inhibitor activity
chemokine receptor binding
chemokine activity

4c

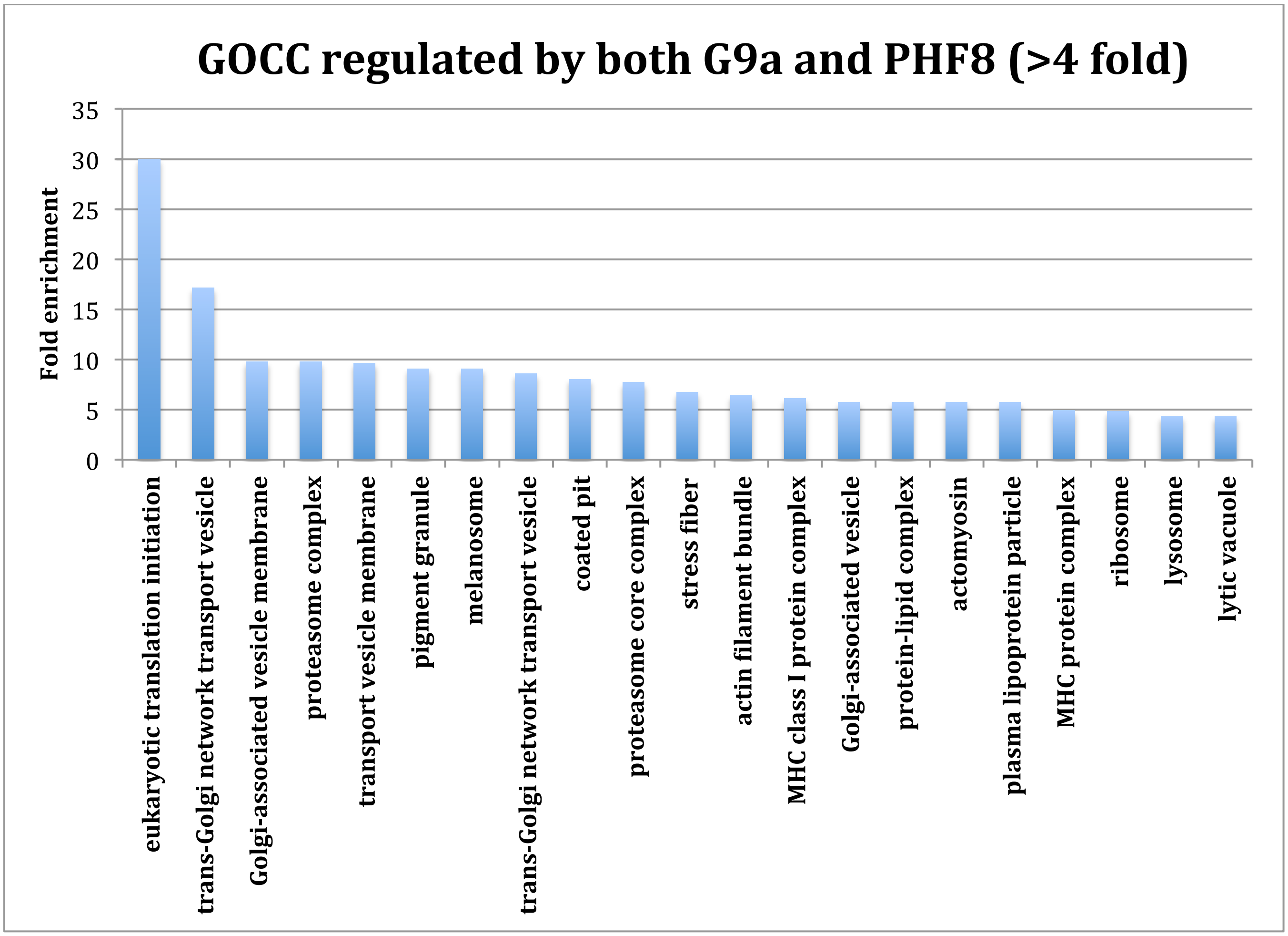

Supplementary Figure 4 G9a and PHF8 regulate a broad range of innate-immunity related protein secretion in an opposite manner to create different inflammatory phenotypes in macrophages. The enrichment of **(a)** GO Biological Processes (GOBP) **(b)** GO Molecular Functions (GOMF) **(c)** GO Cellular Components (GOCC) of the secretome regulated by G9a and PHF8 antagonistically. GOBP and GOMF show the number of proteins identified in each category while GOCC shows the fold-enrichment of the CC.

Cropped Immunoblots:
Fig. 1b

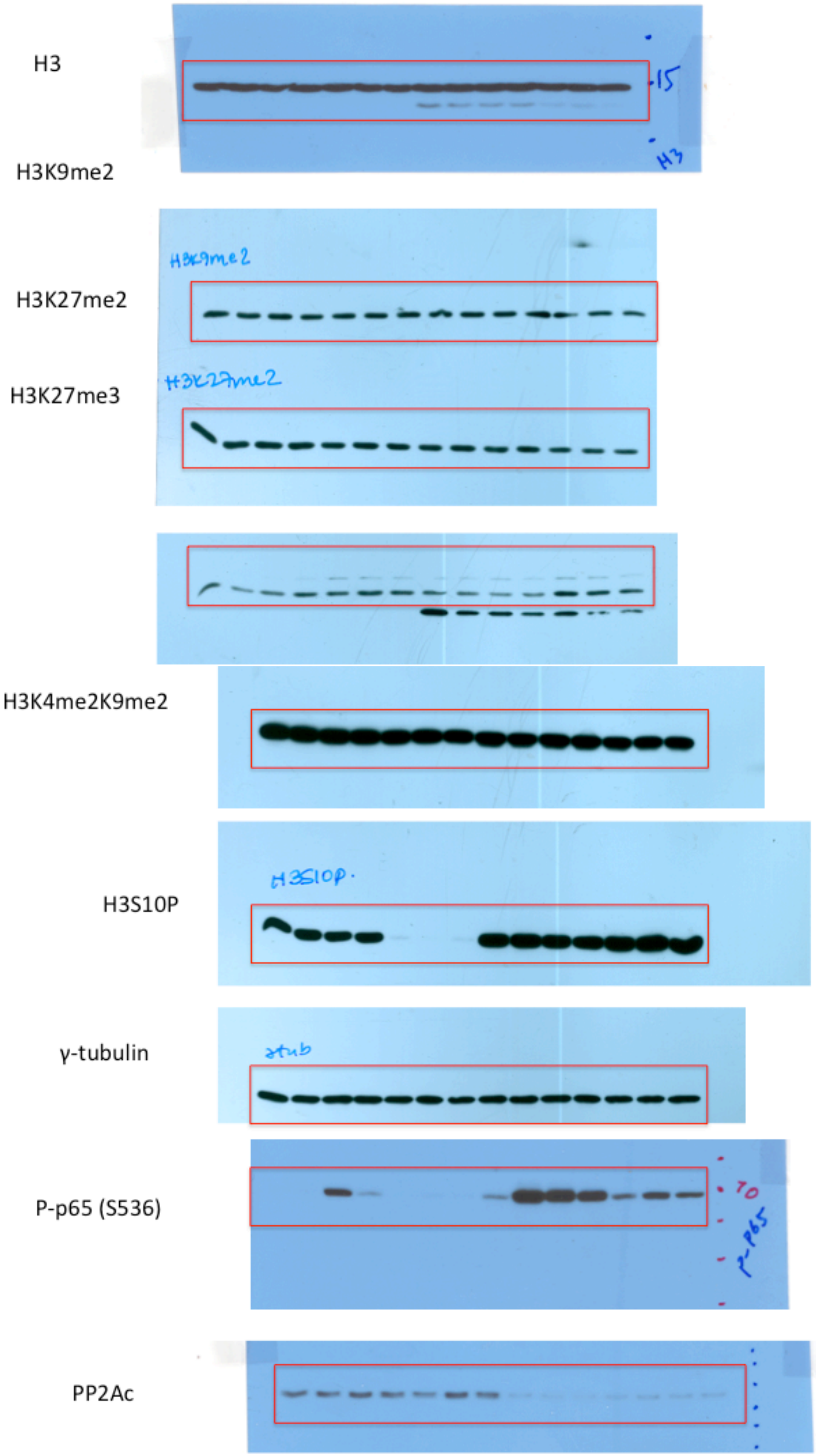

Fig. 2

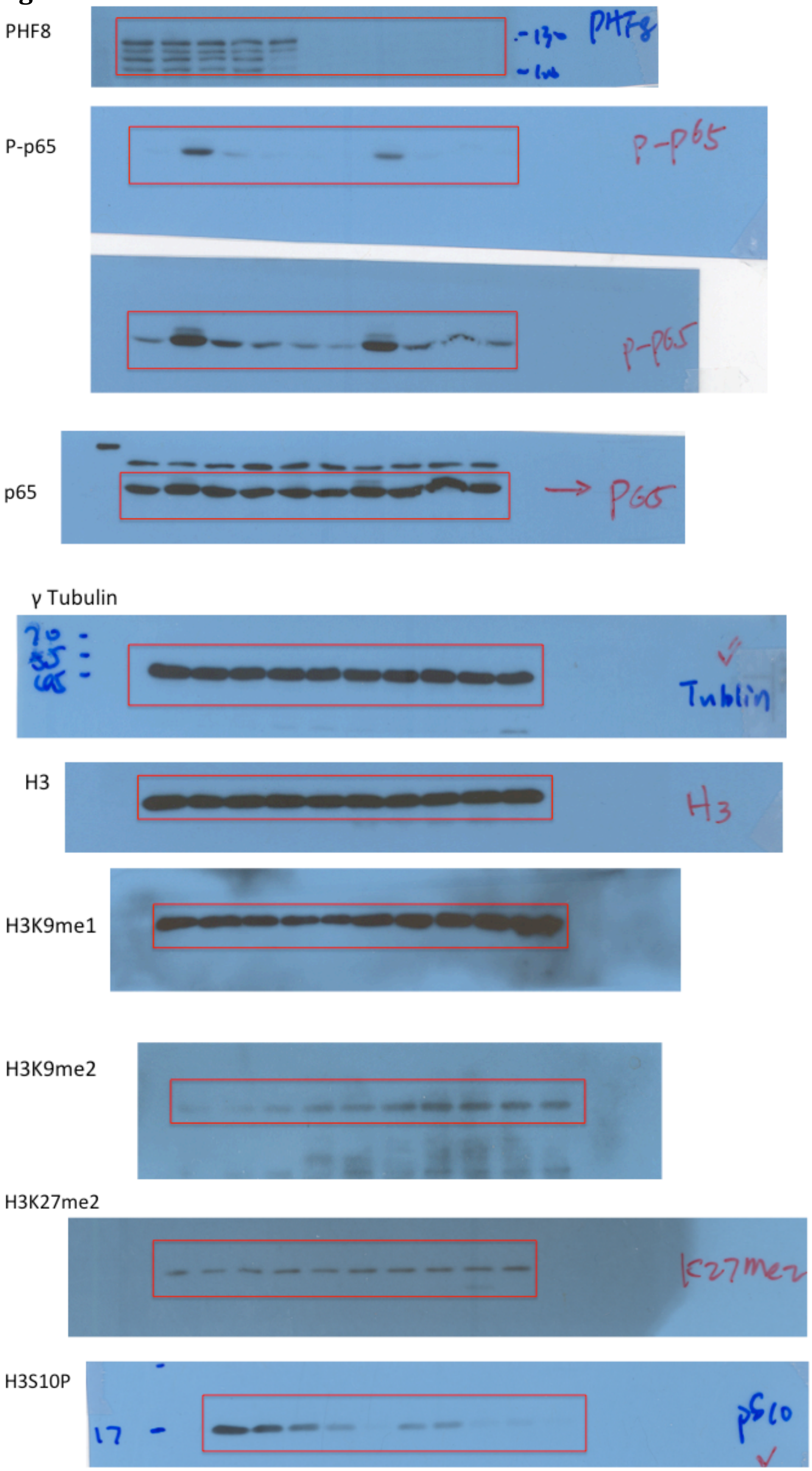

Fig. 3a

Cytosol:

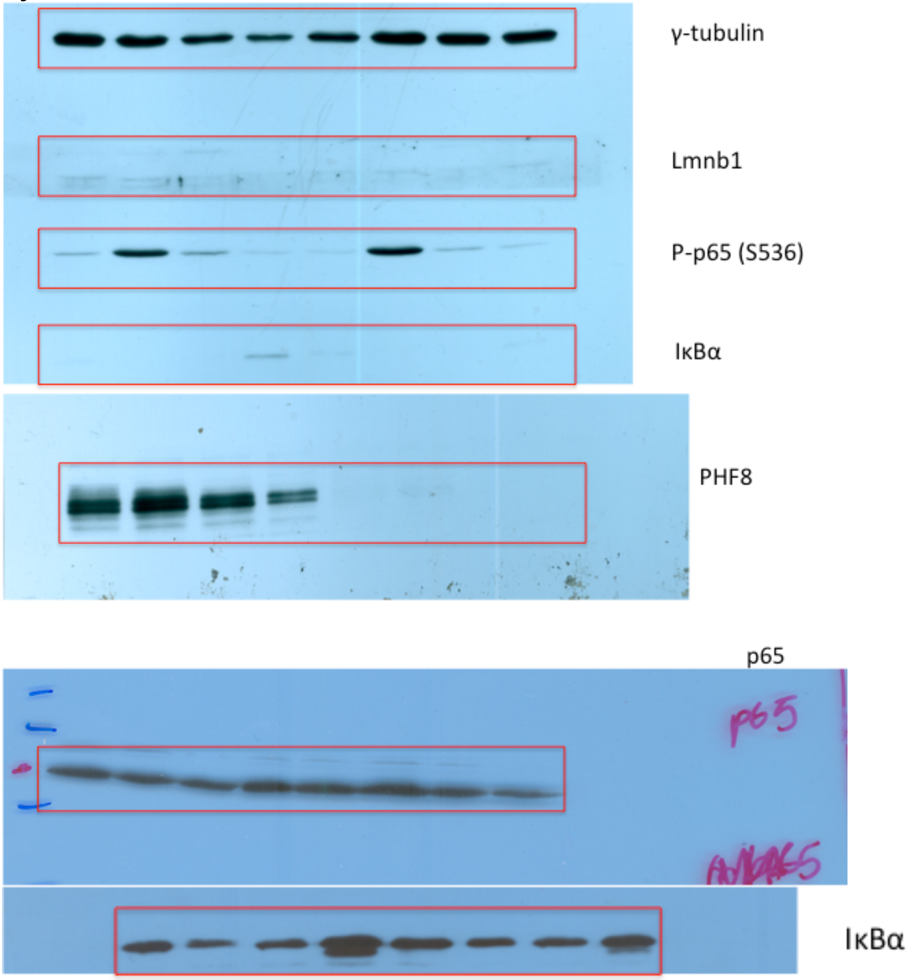

Nucleus:

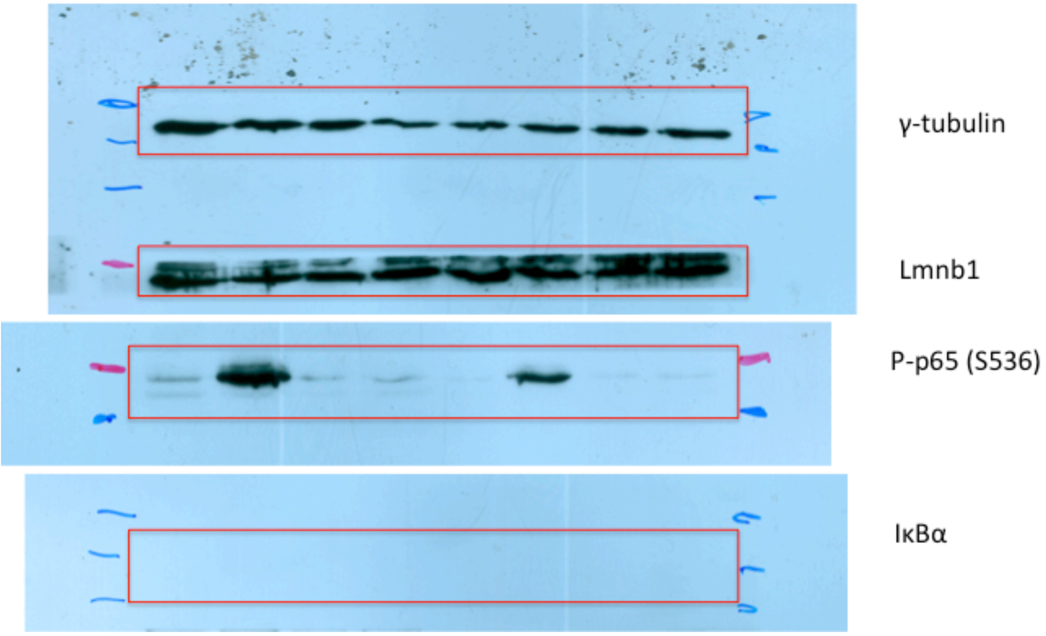

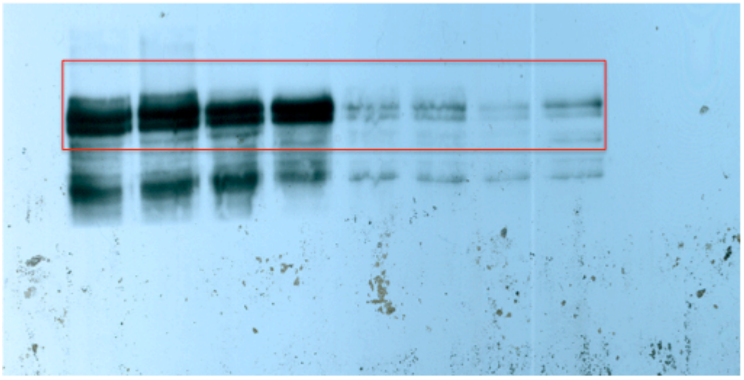

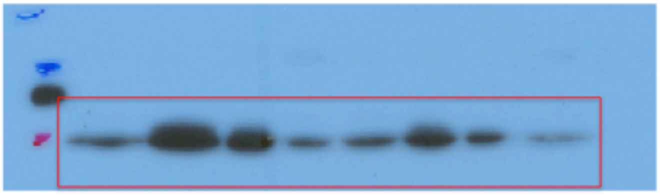

Fig. 3b Input

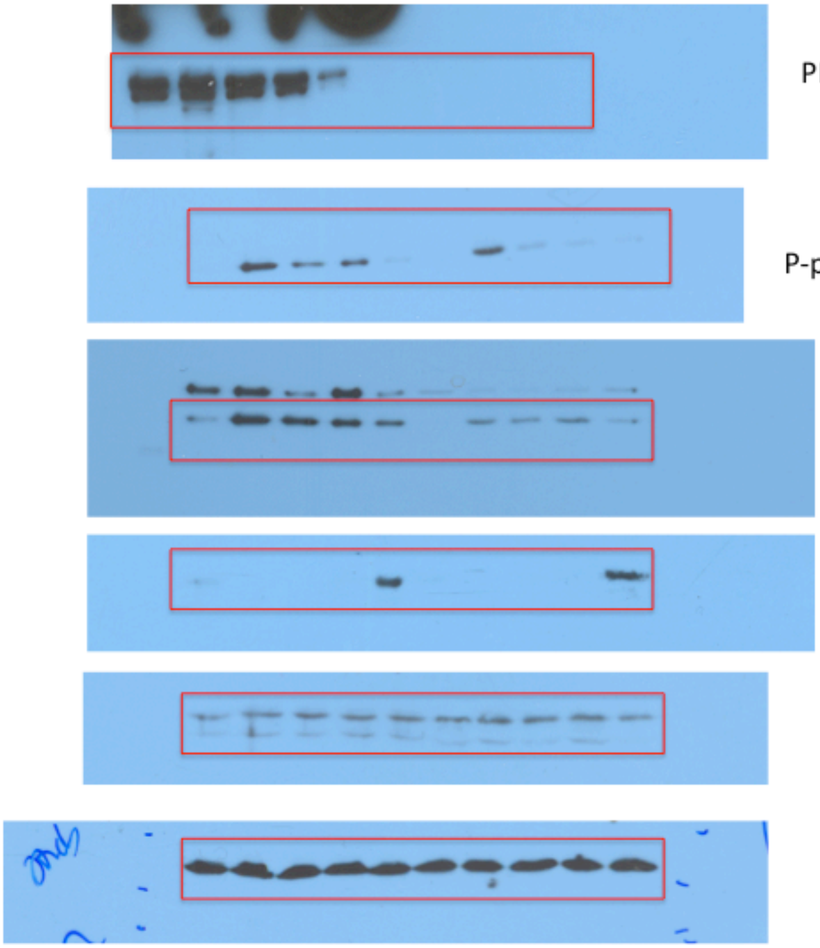

IP:PHF8

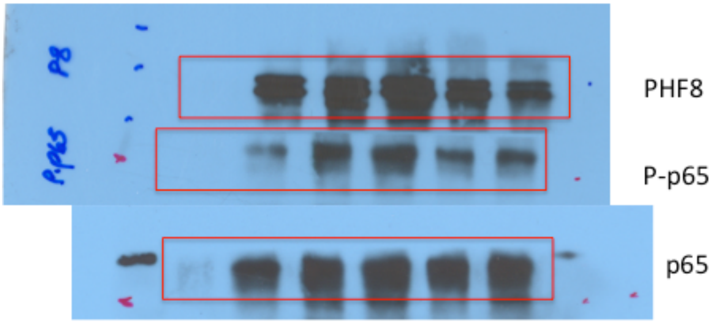

IP:P65 (WT Left, PHF8-KD Right)

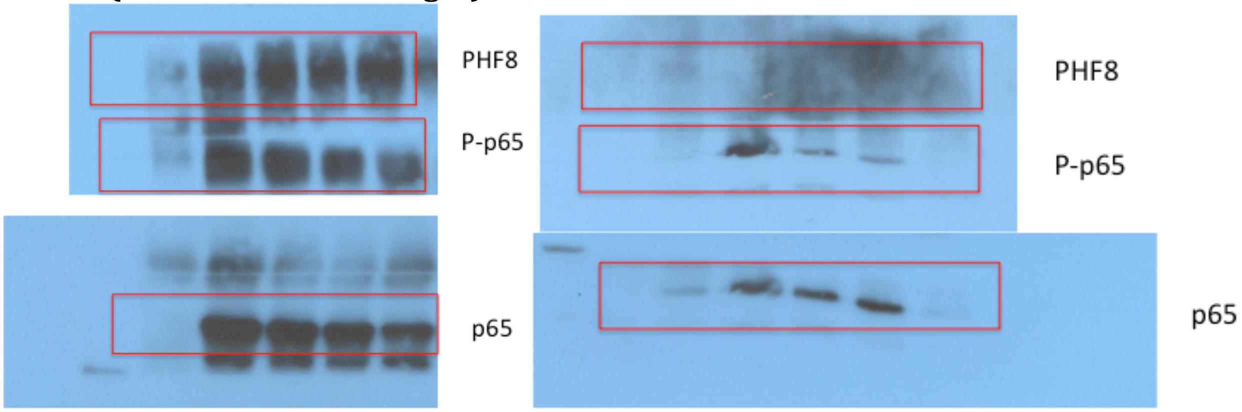

Fig. 3d

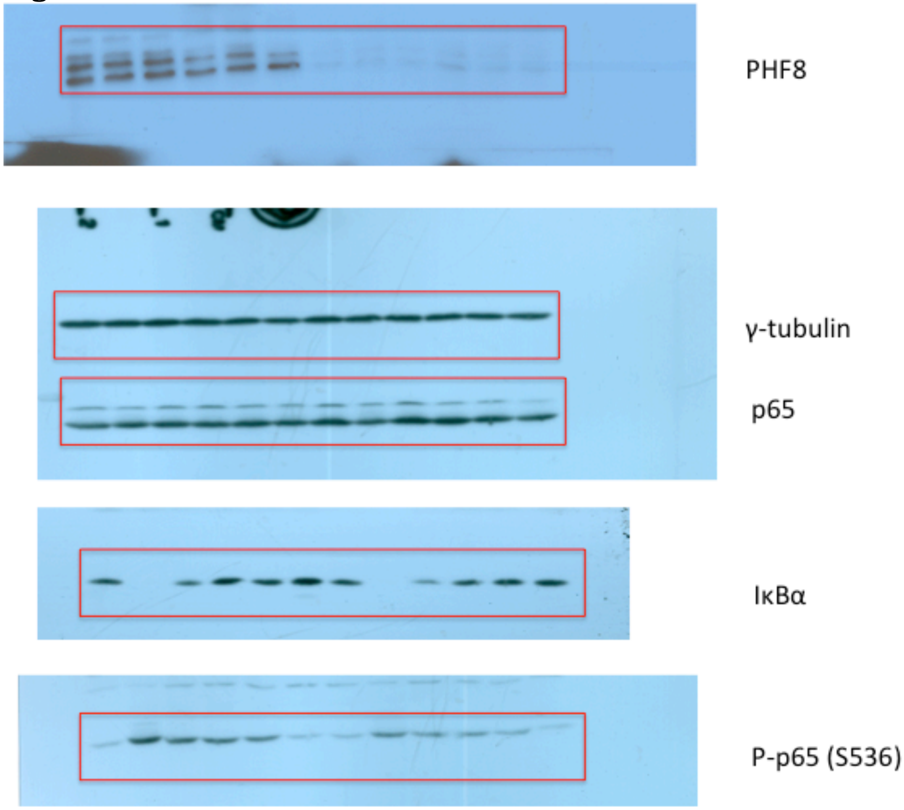

Supplementary Table 1 LPS-inducible PHF8-dependent 'T-class' secretome.

Uniprot ID	Gene name
Q921H8	Acaa1a
Q8CAY6	Acat2
Q91V92	Acly
Q99KI0	Aco2
Q9Z0F8	Adam17
E9Q359	Adam8
P28474	Adh5
P28650	Adssl1
E9Q616	Ahnak
P31230	Aimp1
Q9JII6	Akr1a1
P45376	Akr1b1
Q9EST5	Anp32b
E9Q5H2	Anp32e
Q60709	Aplp2
P12023	App
Q5XJY5	Arcn1
Q9CWJ9	Atic
Q3TKX1	Atp6ap1
Q9CYN9	Atp6ap2
P62814	Atp6v1b2
P01887	B2m
E9QAI5	Cad
P10148	Ccl2
Q03366	Ccl7
P51670	Ccl9
P15379	Cd44
P04441	Cd74
P04186	Cfb
P06909	Cfh
B1AWE0	Clta
Q68FD5	Cltc
Q8K4Q8	Colec12
P61924	Copz1
Q9WUM4	Coro1c
Q61147	Cp
O88668	Creg1
P09581	Csf1r
P21460	Cst3
Q8R242	Ctbs
Q9CWL8	Ctnnbl1
P10605	Ctsb
Q9R013	Ctsf
P06797	Ctsl1
O70370	Ctss
Q9WUU7	Ctsz
D3YW23	Cxcl10
I7HIQ2	Cxcl16
P10889	Cxcl2
Q91UZ6	D17H6S56E-5
Q62165	Dag1
Q922B2	Dars
Q62418	Dbnl
A2ADY9	Ddi2
Q91WV0	Dr1
Q9CQ43	Dut
O35228	Ebi3
Q61508	Ecm1
Q9QZD9	Eif3i
Q91VC3	Eif4a3
P19096	Fasn
Q920E5	Fdps
Q9R059	Fhl3
Q04646	Fxyd2
Q9R0N0	Galk1
P16858	Gapdh
Q64737	Gart
E9Q7H5	Gm8991
P05202	Got2
P28798	Grn
O09131	Gsto1
P01900	H2-D1
P01902	H2-K1
P01897	H2-L
Q8HWB2	H2-Q4
F8VQG4	H2-T24
Q3THW5	H2afv
P70288	Hdac2
P20060	Hexb
P43276	Hist1h1b
P15864	Hist1h1c
P43277	Hist1h1d
P43274	Hist1h1e
Q8CGP6	Hist1h2ah
F8WIX8	Hist1h2al
Q6ZWY9	Hist1h2bc
P84228	Hist1h3b
P62806	Hist1h4a
Q9CX86	Hnrnpa0
O88569	Hnrnpa2b1
Q9Z204	Hnrnpc
O35737	Hnrnph1
Q8R081	Hnrnpl
G3XA10	Hnrnpu
P07901	Hsp90aa1
Q8BM72	Hspa13
P38647	Hspa9
P63038	Hspd1
Q64433	Hspe1
Q8BU30	Iars
P13597	Icam1
Q9ESY9	Ifi30
O35664	Ifnar2
P47879	Igfbp4
Q8K3I6	Il27
Q45VK5	Ilf3
P24547	Impdh2
Q792F9	Itga4
O88188	Ly86
P08905	Lyz2
O09159	Man2b1
Q3THS6	Mat2a
Q8K310	Matr3
P97310	Mcm2
P49717	Mcm4
P97311	Mcm6
Q61881	Mcm7
P08249	Mdh2
Q8VE43	Metrnl
Q7TPV4	Mybbp1a
Q3V4D5	Naa10
Q6PGB6	Naa50
Q9QWR8	Naga
Q78ZA7	Nap1l4
P09405	Ncl
Q62433	Ndrg1
Q9D0T1	Nhp2l1
E9Q5C9	Nolc1
Q99K48	Nono
Q9Z0J0	Npc2
Q61937	Npm1
Q02819	Nucb1
P29758	Oat
Q9CZ30	Ola1
Q8R357	Olfm1
Q62422	Ostf1
Q921K2	Parp1
P31240	Pdgfb
P12382	Pfkl
Q8C605	Pfkp
Q8CHP8	Pgp
Q61753	Phgdh
P27612	Plaa
P35456	Plaur
E9QPE8	Plec
Q9R0E2	Plod1
Q9R0B9	Plod2
Q9R0E1	Plod3
P55065	Pltp
Q923G2	Polr2h
Q8CIH9	Ppat
Q9CR16	Ppid
Q9D0W5	Ppil1
Q61074	Ppm1g
O88531	Ppt1
F6SPQ1	Ppt2
Q7TMR0	Prcp
Q9JIF0	Prmt1
Q99KP6	Prpf19
G3UXL2	Prps1l3
E9PZ00	Psap
O55234	Psmb5
O89086	Rbm3
Q91VM5	Rbmxl1
Q8BK67	Rcc2
Q9JJH1	Rnase4
Q8VEE4	Rpa1
P62983	Rps27a
P60122	Ruvbl1
Q9WTM5	Ruvbl2
P14069	S100a6
D3YXK2	Safb
P32020	Scp2
O35988	Sdc4
Q61112	Sdf4
P17563	Selenbp1
Q62178	Sema4a
Q62179	Sema4b
O09126	Sema4d
E9Q4Q2	Sf1
Q8K4Z5	Sf3a1
G3UVU2	Sf3a2
Q9D554	Sf3a3
G5E866	Sf3b1
Q3UJB0	Sf3b2
Q921M3	Sf3b3
Q923D4	Sf3b5
Q8VIJ6	Sfpq
Q8BJU0	Sgta
Q9D7I0	Shisa5
Q9CZN7	Shmt2
Q6P6I8	Sirpa
Q6P5F6	Slc39a10
E9Q748	Slpi
Q62189	Snrpa
Q78ZM0	Snx3
P13609	Srgn
Q99MR6	Srrt
Q6PDM2	Srsf1
P84104	Srsf3
Q8BL97	Srsf7
Q8BH40	Stx7
G3X956	Supt16
O08784	Tcof1
P40142	Tkt
Q61029	Tmpo
P41274	Tnfsf9
Q8BFY9	Tnpo1
Q9ER38	Tor3a
O89023	Tpp1
Q7M739	Tpr
Q99NH8	Trem2
Q9Z0P5	Twf2
Q8CDN6	Txnl1
P26369	U2af2
Q9Z1F9	Uba2

Q9JIK5	Ddx21
Q9Z1N5	Ddx39b
Q61656	Ddx5
P00375	Dhfr
O35286	Dhx15
O70133	Dhx9
P63037	Dnaja1
Q91YW3	Dnajc3
Q6NZB0	Dnajc8
Q8C255	Dpep2

Q542I8	Itgb2
O89051	Itm2b
P52293	Kpna2
P70168	Kpnb1
Q61792	Lasp1
P35951	Ldlr
Q07797	Lgals3bp
P48678	Lmna
P14733	Lmnb1
P11152	Lpl

O88685	Psmc3
Q3TXS7	Psmd1
Q9D8W5	Psmd12
O35226	Psmd4
P26516	Psmd7
P29351	Ptpn6
Q9JKF6	Pvrl1
Q8BND5	Qsox1
Q5SW88	Rab1
P46061	Rangap1

Q80X50	Ubap2l
Q9JKB1	Uchl3
Q9JMA1	Usp14
D3YYD5	Vps29
Q9EQH3	Vps35
P61965	Wdr5
Q9CQV8	Ywhab
P10404	N/A

Supplementary Table 2 LPS-inducible PHF8-dependent 'NT-class' secretome

Uniprot ID	Gene name
Q8BGQ7	Aars
P63260	Actg1
Q8BK64	Ahsa1
Q61024	Asns
P50516	Atp6v1a
P62204	Calm1
Q04447	Ckb
Q9Z1Q5	Clic1
Q8CIE6	Copa
Q9JIF7	Copb1
O55029	Copb2
O89079	Cope
Q9QZE5	Copg1
O89053	Coro1a
Q9ERK4	Cse1l
P97821	Ctsc
Q9JHU4	Dync1h1
Q8CGC7	Eprs
P97855	G3bp1
Q9CQM9	Glrx3
P70349	Hint1
Q8JZK9	Hmgcs1
Q9Z2X1	Hnrnpf
P11499	Hsp90ab1
G5E8F1	Itgam
P09056	Lif
P17897	Lyz1
P31938	Map2k1
Q9CQT1	Mri1
Q922D8	Mthfd1
Q99J77	Nans
Q7TQI3	Otub1

Uniprot ID	Gene name
Q9QUR7	Pin1
P58389	Ppp2r4
P62192	Psmc1
P46471	Psmc2
P54775	Psmc4
P62196	Psmc5
P62334	Psmc6
Q8BG32	Psmd11
Q9WVJ2	Psmd13
Q8VDM4	Psmd2
P14685	Psmd3
Q99JI4	Psmd6
Q8BGJ5	Ptbp1
D3Z7C6	Ptges3
Q60972	Rbbp4
Q60973	Rbbp7
Q91VI7	Rnh1
P07091	S100a4
Q920A5	Scpep1
Q3TMX0	Sdcbp
Q9EPK6	Sil1
Q78PY7	Snd1
Q08943	Ssrp1
Q80YX1	Tnc
P06804	Tnf
Q62318	Trim28
E9PXX7	Txndc5
Q02053	Uba1
Q9WUP7	Uchl5
Q9Z1Z0	Uso1
Q3U4W8	Usp5
P20152	Vim

Supplementary Table 3 Secreted proteins found in common with previous LPS-induced BMDM secretome study from Mann group[1].

Secretome in common with Meissner et al.[1]					
Uniprot ID	Gene name	Uniprot ID	Gene name	Uniprot ID	Gene name
Q99KI0	Aco2	P09528	Fth1	Q9QWR8	Naga
O35598	Adam10	Q9CPX4	Ftl1	O88325	Naglu
Q9Z0F8	Adam17	P23188	Furin	O09043	Napsa
P54923	Adprh	P70699	Gaa	Q8VEJ4	Nle1

P10518	Alad	Q8BHN3	Ganab	Q11011	Npepps
E9Q4G8	Alcam	P17439	Gba	O35375	Nrp2
P05064	Aldoa	Q60648	Gm2a	Q02819	Nucb1
Q60709	Aplp2	Q8BFR4	Gns	P29758	Oat
P12023	App	Q99P91	Gpnmb	P09103	P4hb
Q9WV54	Asah1	P28798	Grn	P29341	Pabpc1
Q9CYN9	Atp6ap2	P01900	H2-D1	Q9WU78	Pdcd6ip
P01887	B2m	P01902	H2-K1	P31240	Pdgfb
Q09200	B4galnt1	Q8HWB2	H2-Q4	P08003	Pdia4
Q9JMK0	B4galt5	P06339	H2-T23	Q922R8	Pdia6
Q91XV3	Basp1	F8VQG4	H2-T24	P35456	Plaur
P01027	C3	P29416	Hexa	Q3TCN2	Plbd2
E9QAI5	Cad	P20060	Hexb	Q8BG07	Pld4
P14211	Calr	P97825	Hn1	Q9R0B9	Plod2
P10148	Ccl2	Q6PGH2	Hn1l	Q9R0E1	Plod3
O88430	Ccl22	P08113	Hsp90b1	B2RXS4	Plxnb2
P10855	Ccl3	Q8BM72	Hspa13	P24369	Ppib
P14097	Ccl4	P20029	Hspa5	O88531	Ppt1
P30882	Ccl5	P63038	Hspd1	F6SPQ1	Ppt2
Q03366	Ccl7	Q64433	Hspe1	Q7TMR0	Prcp
P51670	Ccl9	Q9JKR6	Hyou1	O08795	Prkcsh
P10810	Cd14	P13597	Icam1	Q64695	Procr
P15379	Cd44	Q9JHJ8	Icoslg	Q08761	Pros1
P04441	Cd74	Q9ESY9	Ifi30	E9PZ00	Psap
P06909	Cfh	O35664	Ifnar2	B0V2N1	Ptprs
P11680	Cfp	P01575	Ifnb1	Q9JKF6	Pvrl1
Q9D8B3	Chmp4b	Q3TBV5	Il1rn	O89086	Rbm3
Q8WTY4	Ciapin1	Q8K3I6	Il27	Q9CQ01	Rnaset2
Q68FD5	Cltc	P34902	Il2rg	Q5XJF6	Rpl10a
O89001	Cpd	O09046	Il4i1	P07091	S100a4
O88668	Creg1	P08505	Il6	P04918	Saa3
P07141	Csf1	Q64339	Isg15	Q920A5	Scpep1
P09920	Csf3	Q792F9	Itga4	O35988	Sdc4
P21460	Cst3	G5E8F1	Itgam	Q3TMX0	Sdcbp
Q8R242	Ctbs	Q542I8	Itgb2	Q62179	Sema4b
P16675	Ctsa	O89051	Itm2b	P22777	Serpine1
P10605	Ctsb	P11672	Lcn2	P10923	Spp1
P97821	Ctsc	P35951	Ldlr	P13609	Srgn
P18242	Ctsd	Q07797	Lgals3bp	P54227	Stmn1
O70370	Ctss	O89017	Lgmn	Q8R0B4	Tardbp
Q9WUU7	Ctsz	Q64281	Lilrb4	O88968	Tcn2
D3YW23	Cxcl10	Q9WVG5	Lipg	Q62351	Tfrc
I7HIQ2	Cxcl16	Q9DBH5	Lman2	Q8C1A5	Thop1
P10889	Cxcl2	P11152	Lpl	P12032	Timp1
Q91UZ6	D17H6S56E-5	Q91ZX7	Lrp1	P06804	Tnf
Q9CPT4	D17Wsu104e	P19973	Lsp1	P41274	Tnfsf9
Q91YW3	Dnajc3	O88188	Ly86	Q62393	Tpd52
O35228	Ebi3	O09159	Man2b1	O89023	Tpp1
Q61508	Ecm1	O54782	Man2b2	Q99NH8	Trem2
E9QN08	Eef1d	Q8K2I4	Manba	E9PXX7	Txndc5
P63242	Eif5a	P08249	Mdh2	Q6P5E4	Uggt1
Q8K482	Emilin2	Q8VE43	Metrnl	P20152	Vim
Q9EQH2	Erap1	Q9Z2L6	Minpp1	P10404	Env1
P57759	Erp29	P26041	Msn		
Q9D1Q6	Erp44	Q3V4D5	Naa10		

Supplementary Table 4 Secreted proteins found in common with previous LPS-induced BMDM secretome study from our group[2].

Secretome in common with Liu et al.[2]							
**Uniprot ID**	**Gene name**	**Uniprot ID**	**Gene name**	**Uniprot ID**	**Gene name**	**Uniprot ID**	**Gene name**
P42208	1-Sep	P10126	Eef1a1	P31938	Map2k1	P14115	Rpl27a
O55131	6-Sep	O70251	Eef1b	P63085	Mapk1	P27659	Rpl3
Q80UG5	8-Sep	Q9D8N0	Eef1g	Q61166	Mapre1	P62889	Rpl30
Q8C1B7	10-Sep	P58252	Eef2	Q3THS6	Mat2a	P62900	Rpl31
Q8BGQ7	Aars	Q8C845	Efhd2	Q8K310	Matr3	P62911	Rpl32
Q921H8	Acaa1a	Q9WVK4	Ehd1	P14152	Mdh1	Q9D1R9	Rpl34
Q8CAY6	Acat2	Q9EQP2	Ehd4	P08249	Mdh2	Q6ZWV7	Rpl35
Q91V92	Acly	P48024	Eif1	P34884	Mif	Q9JJI8	Rpl38
P28271	Aco1	Q8BJW6	Eif2a	P26041	Msn	Q9D8E6	Rpl4
Q99KI0	Aco2	Q6ZWX6	Eif2s1	Q9CQ65	Mtap	P47962	Rpl5
Q91V12	Acot7	Q99L45	Eif2s2	Q922D8	Mthfd1	P47911	Rpl6
P63260	Actg1	Q9Z0N1	Eif2s3x	P62774	Mtpn	P14148	Rpl7
P57780	Actn4	P23116	Eif3a	Q9JK81	Myg1	P47955	Rplp1
P61164	Actr1a	Q8JZQ9	Eif3b	Q8VDD5	Myh9	P99027	Rplp2
P61161	Actr2	Q8R1B4	Eif3c	Q60605	Myl6	P63325	Rps10
Q99JY9	Actr3	O70194	Eif3d	Q60817	Naca	P62281	Rps11
P28474	Adh5	P60229	Eif3e	Q9QWR8	Naga	Q6ZWZ6	Rps12
P54923	Adprh	Q9DCH4	Eif3f	O88325	Naglu	P62301	Rps13
P46664	Adss	Q9Z1D1	Eif3g	Q99KQ4	Nampt	P62264	Rps14
P28650	Adssl1	Q91WK2	Eif3h	Q99J77	Nans	P62843	Rps15
P50247	Ahcy	Q9QZD9	Eif3i	Q78ZA7	Nap1l4	P62245	Rps15a
E9Q616	Ahnak	Q3UGC7	Eif3j1	Q8BP47	Nars	P14131	Rps16
Q8BK64	Ahsa1	Q8QZY1	Eif3l	B1AU76	Nasp	P63276	Rps17
P31230	Aimp1	P60843	Eif4a1	P09405	Ncl	Q9CZX8	Rps19
Q9WTP6	Ak2	Q8BGD9	Eif4b	P29595	Nedd8	P25444	Rps2
Q9JII6	Akr1a1	P63073	Eif4e	Q9D0T1	Nhp2l1	P60867	Rps20
P45376	Akr1b1	Q9WUK2	Eif4h	Q9JHW2	Nit2	Q9CQR2	Rps21
P47738	Aldh2	P59325	Eif5	P15532	Nme1	P62267	Rps23
Q9JLJ2	Aldh9a1	P63242	Eif5a	Q99K48	Nono	P62849	Rps24
P05064	Aldoa	O55135	Eif6	Q9Z0J0	Npc2	P62852	Rps25
P05063	Aldoc	P70372	Elavl1	Q11011	Npepps	P62855	Rps26
O08583	Alyref	P17182	Eno1	A6PWC3	Nrd1	P62983	Rps27a
O35381	Anp32a	Q8CGC7	Eprs	Q9CZ44	Nsfl1c	P62908	Rps3
Q9EST5	Anp32b	P84089	Erh	Q02819	Nucb1	P62702	Rps4x
E9Q5H2	Anp32e	P57759	Erp29	Q3UKN6	Nucb2	P62754	Rps6
P07356	Anxa2	Q9D1Q6	Erp44	O35685	Nudc	P62242	Rps8
Q5SVG5	Ap1b1	Q9R0P3	Esd	P61971	Nutf2	Q6ZWN5	Rps9
P17426	Ap2a1	Q8BWY3	Etf1	Q9CZ30	Ola1	P14206	Rpsa
Q9DBG3	Ap2b1	Q5SUT0	Ewsr1	Q62422	Ostf1	A2AVJ7	Rrbp1
Q8R5A3	Apbb1ip	P26040	Ezr	Q7TQI3	Otub1	P60122	Ruvbl1
O35841	Api5	Q05816	Fabp5	P09103	P4hb	P50543	S100a11
P12023	App	Q8R1F1	Fam129b	P50580	Pa2g4	P07091	S100a4
P08030	Aprt	Q921M7	Fam49b	P29341	Pabpc1	P14069	S100a6
Q5XJY5	Arcn1	E9PWY9	Farsa	P63005	Pafah1b1	P04918	Saa3
P84078	Arf1	Q9WUA2	Farsb	Q61206	Pafah1b2	Q9R1T2	Sae1
Q99PT1	Arhgdia	P19096	Fasn	Q9DCL9	Paics	Q60710	Samhd1
Q61599	Arhgdib	Q920E5	Fdps	Q8CIN4	Pak2	Q9D1J3	Sarnp
Q9WV32	Arpc1b	Q91Z50	Fen1	Q99LX0	Park7	P26638	Sars
Q9CVB6	Arpc2	Q8K1B8	Fermt3	Q921K2	Parp1	P32020	Scp2
Q9JM76	Arpc3	P97807	Fh	P60335	Pcbp1	Q920A5	Scpep1

P59999	Arpc4	P26883	Fkbp1a	Q61990	Pcbp2	O35988	Sdc4
Q9CPW4	Arpc5	Q62446	Fkbp3	P23506	Pcmt1	Q3TMX0	Sdcbp
P50429	Arsb	B7FAU9	Flna	P17918	Pcna	Q61112	Sdf4
Q9WV54	Asah1	Q9CPX4	Ftl1	P56812	Pdcd5	Q9D662	Sec23b
Q9CWJ9	Atic	P97855	G3bp1	Q9WU78	Pdcd6ip	G3X972	Sec24c
O08997	Atox1	Q00612	G6pdx	P27773	Pdia3	P17563	Selenbp1
Q3TKX1	Atp6ap1	P70699	Gaa	P08003	Pdia4	Q8BH69	Sephs1
Q9CYN9	Atp6ap2	Q9R0N0	Galk1	Q8K183	Pdxk	Q60854	Serpinb6
P50516	Atp6v1a	Q8BHN3	Ganab	P70296	Pebp1	A2BE93	Set
P62814	Atp6v1b2	P16858	Gapdh	O70591	Pfdn2	Q8K4Z5	Sf3a1
P01887	B2m	Q9CZD3	Gars	Q9WU28	Pfdn5	G5E866	Sf3b1
Q91XV3	Basp1	P17439	Gba	P12382	Pfkl	Q921M3	Sf3b3
Q07813	Bax	Q61598	Gdi2	Q9DBJ1	Pgam1	Q8VIJ6	Sfpq
Q8R016	Blmh	Q9CPV4	Glod4	Q9DCD0	Pgd	Q8BJU0	Sgta
Q9CY64	Blvra	Q9QUH0	Glrx	P09411	Pgk1	Q9JJU8	Sh3bgrl
Q923D2	Blvrb	Q9CQM9	Glrx3	Q9CQ60	Pgls	Q91VW3	Sh3bgrl3
Q64152	Btf3	E9PZF0	Gm20390	Q9D0F9	Pgm1	Q78PY7	Snd1
P01027	C3	Q60648	Gm2a	Q61753	Phgdh	P62305	Snrpe
Q9CXW3	Cacybp	J3QP68	Gm4204	Q9DAK9	Phpt1	Q6NZD2	Snx1
P62204	Calm1	Q9CQI3	Gmfb	Q7M6Y3	Picalm	Q9CWK8	Snx2
P14211	Calr	P62880	Gnb2	P53810	Pitpna	Q78ZM0	Snx3
Q6ZQ38	Cand1	P68040	Gnb2l1	P52480	Pkm	Q9D8U8	Snx5
P40124	Cap1	O88958	Gnpda1	P52480	Pkm	Q6P8X1	Snx6
Q99LB4	Capg	Q8BFR4	Gns	Q3TCN2	Plbd2	Q91VH2	Snx9
Q60865	Caprin1	P06745	Gpi	Q8CIH5	Plcg2	P08228	Sod1
Q5RKN9	Capza1	Q99P91	Gpnmb	Q8BG07	Pld4	P10923	Spp1
P47754	Capza2	Q60631	Grb2	E9QPE8	Plec	P13609	Srgn
P47757	Capzb	P28798	Grn	Q9R0E2	Plod1	Q6PDM2	Srsf1
Q9DCC5	Cbx3	P13020	Gsn	P55065	Pltp	Q62093	Srsf2
P10148	Ccl2	Q8R050	Gspt1	Q543K9	Pnp	P84104	Srsf3
P10855	Ccl3	O09131	Gsto1	Q9D819	Ppa1	P32067	Ssb
P14097	Ccl4	Q8HWB2	H2-Q4	P17742	Ppia	F8WJK8	St13
P30882	Ccl5	Q3THW5	H2afv	P24369	Ppib	Q60864	Stip1
P51670	Ccl9	Q61035	Hars	Q9CR16	Ppid	Q9Z1Z2	Strap
P80314	Cct2	P49710	Hcls1	Q61074	Ppm1g	Q8BH40	Stx7
P80318	Cct3	P51859	Hdgf	P62137	Ppp1ca	Q64324	Stxbp2
P80315	Cct4	P29416	Hexa	Q3UM45	Ppp1r7	P11031	Sub1
P80316	Cct5	P20060	Hexb	P63330	Ppp2ca	Q9CX34	Sugt1
P80317	Cct6a	P70349	Hint1	Q76MZ3	Ppp2r1a	Q6A028	Swap70
P80313	Cct7	P43276	Hist1h1b	Q6P1F6	Ppp2r2a	Q7TMK9	Syncrip
P42932	Cct8	P43277	Hist1h1d	P58389	Ppp2r4	Q9WVA4	Tagln2
P10810	Cd14	P43274	Hist1h1e	O88531	Ppt1	Q93092	Taldo1
P04441	Cd74	Q8CGP6	Hist1h2ah	P35700	Prdx1	P48428	Tbca
Q61081	Cdc37	P84228	Hist1h3b	Q61171	Prdx2	P10711	Tcea1
P60766	Cdc42	P62806	Hist1h4a	P99029	Prdx5	P83940	Tceb1
P04186	Cfb	G3UVV4	Hk1	Q9QUR6	Prep	P62869	Tceb2
P18760	Cfl1	P63158	Hmgb1	Q9DBC7	Prkar1a	P11983	Tcp1
P11680	Cfp	P30681	Hmgb2	O08795	Prkcsh	Q9Z1A1	Tfg
Q9D8B3	Chmp4b	P14901	Hmox1	Q9Z2Y8	Prosc	Q9WVA2	Timm8a1
Q9D1P4	Chordc1	P97825	Hn1	Q99KP6	Prpf19	P40142	Tkt
Q04447	Ckb	Q6PGH2	Hn1l	E9PZ00	Psap	P26039	Tln1
Q9Z1Q5	Clic1	Q9CX86	Hnrnpa0	Q99K85	Psat1	P06804	Tnf
Q9QYB1	Clic4	P49312	Hnrnpa1	Q9Z2U1	Psma5	P25119	Tnfrsf1b
B1AWE0	Clta	O88569	Hnrnpa2b1	Q9QUM9	Psma6	Q62393	Tpd52
Q68FD5	Cltc	Q99020	Hnrnpab	Q9Z2U0	Psma7	Q3TUJ9	Tpd52l2
Q9DBP5	Cmpk1	Q9Z204	Hnrnpc	P62192	Psmc1	P17751	Tpi1

Q3U5Q7	Cmpk2	Q9Z2X1	Hnrnpf	P46471	Psmc2	P21107	Tpm3
Q9D1A2	Cndp2	G3XA10	Hnrnpu	P54775	Psmc4	Q6IRU2	Tpm4
Q8CIE6	Copa	P00493	Hprt1	P62196	Psmc5	O89023	Tpp1
Q9JIF7	Copb1	P07901	Hsp90aa1	P62334	Psmc6	Q64514	Tpp2
O55029	Copb2	P11499	Hsp90ab1	Q3TXS7	Psmd1	P63028	Tpt1
O89079	Cope	P08113	Hsp90b1	Q8BG32	Psmd11	Q99NH8	Trem2
Q9QZE5	Copg1	Q3U2G2	Hspa4	Q9D8W5	Psmd12	Q62318	Trim28
P61202	Cops2	P20029	Hspa5	Q9WVJ2	Psmd13	Q9DCG9	Trmt112
O88543	Cops3	P63017	Hspa8	O35593	Psmd14	P68373	Tuba1c
O88544	Cops4	P38647	Hspa9	Q8VDM4	Psmd2	P68372	Tubb4b
P61924	Copz1	P63038	Hspd1	P14685	Psmd3	P99024	Tubb5
O89053	Coro1a	Q64433	Hspe1	O35226	Psmd4	Q922F4	Tubb6
Q9WUM3	Coro1b	Q9JKR6	Hyou1	Q99JI4	Psmd6	Q9Z0P5	Twf2
Q9WUM4	Coro1c	Q8BU30	Iars	P26516	Psmd7	P10639	Txn
Q9CQI6	Cotl1	O88844	Idh1	G3UXZ5	Psme1	Q9CQM5	Txndc17
Q6NVF9	Cpsf6	Q9ESY9	Ifi30	G3X9V0	Psme2	E9PXX7	Txndc5
O88668	Creg1	P08505	Il6	Q8BGJ5	Ptbp1	Q8CDN6	Txnl1
P63254	Crip1	Q924B0	Impa1	P29351	Ptpn6	Q9JMH6	Txnrd1
P09581	Csf1r	Q8BKC5	Ipo5	Q3UEB3	Puf60	Q3TW96	Uap1l1
P21460	Cst3	E9QKZ2	Ipo9	D3YWR7	Qdpr	Q02053	Uba1
Q62426	Cstb	Q9JKF1	Iqgap1	P35278	Rab5c	Q9Z1F9	Uba2
P16675	Ctsa	Q64339	Isg15	P51150	Rab7a	P68037	Ube2l3
P10605	Ctsb	Q91V64	Isoc1	P63001	Rac1	P61089	Ube2n
P97821	Ctsc	Q9JHU9	Isyna1	Q05144	Rac2	Q9CZY3	Ube2v1
P18242	Ctsd	G5E8F1	Itgam	P54728	Rad23b	Q9D2M8	Ube2v2
P06797	Ctsl1	Q542I8	Itgb2	P62827	Ran	Q9JKB1	Uchl3
O70370	Ctss	O89051	Itm2b	P34022	Ranbp1	P61961	Ufm1
Q9WUU7	Ctsz	Q99MN1	Kars	Q9D0I9	Rars	Q6P5E4	Uggt1
D3YW23	Cxcl10	Q3U0V1	Khsrp	Q60972	Rbbp4	Q91ZJ5	Ugp2
P10889	Cxcl2	P70168	Kpnb1	O89086	Rbm3	Q9Z1Z0	Uso1
Q99LF4	D10Wsu52e	Q9CPY7	Lap3	Q9CWZ3	Rbm8a	Q9JMA1	Usp14
Q922B2	Dars	Q61792	Lasp1	Q91VM5	Rbmxl1	Q3U4W8	Usp5
P31786	Dbi	P11672	Lcn2	Q8BK67	Rcc2	Q9Z1Q9	Vars
Q62418	Dbnl	Q61233	Lcp1	P26043	Rdx	P70460	Vasp
D3YX34	Dctn1	P06151	Ldha	P82343	Renbp	Q62465	Vat1
A2ADY9	Ddi2	P16045	Lgals1	Q9QUI0	Rhoa	P61759	Vbp1
Q501J6	Ddx17	Q8C253	Lgals3	Q9CQ01	Rnaset2	Q64727	Vcl
Q9Z1N5	Ddx39b	Q07797	Lgals3bp	Q91VI7	Rnh1	Q01853	Vcp
Q61656	Ddx5	O89017	Lgmn	Q8VCT3	Rnpep	P20152	Vim
Q6Q899	Ddx58	P48678	Lmna	I7HLV2	Rpl10	Q9EQH3	Vps35
P54823	Ddx6	P14733	Lmnb1	Q5XJF6	Rpl10a	Q99KC8	Vwa5a
P00375	Dhfr	P11152	Lpl	P35979	Rpl12	P32921	Wars
O35286	Dhx15	Q91ZX7	Lrp1	P47963	Rpl13	P70315	Was
O70133	Dhx9	Q505F5	Lrrc47	P19253	Rpl13a	O88342	Wdr1
Q8K1M6	Dnm1l	P19973	Lsp1	Q9CR57	Rpl14	Q6P1B1	Xpnpep1
Q9Z2W0	Dnpep	P24527	Lta4h	P62717	Rpl18a	Q6P5F9	Xpo1
Q8C255	Dpep2	O88188	Ly86	A2A547	Rpl19	P62960	Ybx1
Q99KK7	Dpp3	Q9WTL7	Lypla2	Q9CQM8	Rpl21	P62259	Ywhae
O08553	Dpysl2	P08905	Lyz2	P67984	Rpl22	P61982	Ywhag
Q9R0P5	Dstn	O09159	Man2b1	P62830	Rpl23	P68510	Ywhah
Q9JHU4	Dync1h1	O54782	Man2b2	E9Q132	Rpl24	P68254	Ywhaq
Q61508	Ecm1	Q80ZP8	Manf	P61358	Rpl27	P63101	Ywhaz

Supplementary Table 5 Proteins whose secretion is regulated by G9a and PHF8 antagonistically

Uniprot ID	Gene Name	Uniprot ID	Gene Name	Uniprot ID	Gene Name	Uniprot ID	Gene Name
P68510	Ywhah	E9Q039	Npepps	P11152	Lpl	P61358	Rpl27
P61982	Ywhag	E9Q132	Rpl24	Q64281-2	Lilrb4	P41105	Rpl28
P63101	Ywhaz	E9Q4G8	Alcam	Q9DBH5	Lman2	P62900	Rpl31
P70445	Eif4ebp2	E9Q4Q2	Sf1	P48678	Lmna	Q9D1R9	Rpl34
A2AFJ1	Rbbp7	E9Q616	Ahnak	Q91ZX7	Lrp1	Q9JJI8	Rpl38
A2AFK7	Eif4a3	E9Q748	Slpi	P17897	Lyz1	P14148	Rpl7
P12023	App	E9Q7G0	Numa1	O09159	Man2b1	P62918	Rpl8
A6PWC3	Nrd1	E9Q7W0	Rbpj	P97310	Mcm2	Q9CQ01	Rnaset2
P05202	Got2	E9QN08	Eef1d	P08249	Mdh2	P47968	Rpia
Q91V92	Acly	E9QPE8	Plec	Q3THS6	Mat2a	P62281	Rps11
P28271	Aco1	Q61508	Ecm1	Q8VE43	Metrnl	P62245	Rps15a
Q99KI0	Aco2	P10126	Eef1a1	Q9JK81	Myg1	P14131	Rps16
P60710	Actb	Q9D8N0	Eef1g	Q60817	Naca	P62267	Rps23
Q8BK64	Ahsa1	Q9EQP2	Ehd4	Q9QWR8	Naga	P62852	Rps25
Q9JII6	Akr1a1	Q8R1B4	Eif3c	Q91WV0	Dr1	P62855	Rps26
Q9JLJ2	Aldh9a1	O70194	Eif3d	P11672	Lcn2	Q9WTM5	Ruvbl2
P45376	Akr1b1	Q9DCH4	Eif3f	Q8R1F1	Fam129b	P62305	Snrpe
P97822-2	Anp32e	Q9Z1D1	Eif3g	Q8VEJ4	Nle1	P07091	S100a4
Q9JIF0-2	Prmt1	Q9QZD9	Eif3i	Q99K48	Nono	P14069	S100a6
P07356	Anxa2	Q9DBZ5	Eif3k	Q9Z0J0	Npc2	P50543	S100a11
P59999	Arpc4	Q8QZY1	Eif3l	O35375-5	Nrp2	P50247	Ahcy
Q61024	Asns	Q99JX4	Eif3m	Q02819	Nucb1	P17563	Selenbp1
Q64191	Aga	P70372	Elavl1	O35685	Nudc	O35988	Sdc4
O08997	Atox1	P17182	Eno1	Q9JKX6	Nudt5	Q9D1M0	Sec13
B0QZX1	Il2rg	P10404	2	P10923	Spp1	Q62178	Sema4a
B1ATI9	Gas7	Q64695	Procr	Q7TQI3	Otub1	O09126	Sema4d
B1AWE0	Clta	Q9EQH2	Erap1	O09046	Il4i1	Q8C1B7-3	42258
P01887	B2m	Q8R050-2	Gspt1	P22777	Serpine1	Q80UG5-2	42256
Q09200	B4galnt1	F6RPJ9	Ide	Q9CY58	Serbp1	Q99LS3	Psph
Q9JMK0	B4galt5	F7CDT0	Ube2m	Q8CIN4	Pak2	Q99K85	Psat1
B7FAU9	Flna	F8VQJ3	Lamc1	P60335	Pcbp1	Q9D554	Sf3a3
Q91V12-2	Acot7	Q9R059	Fhl3	Q9WU78	Pdcd6ip	Q8VIJ6	Sfpq
O54962	Banf1	Q62446	Fkbp3	P23506	Pcmt1	Q91VW3	Sh3bgrl3
Q07813	Bax	Q80X90	Flnb	Q8BG07-2	Pld4	Q9D7I0	Shisa5
Q9CY64	Blvra	Q920E5	Fdps	Q9R0E2	Plod1	Q9EPK6	Sil1
Q8BGS2-2	Bola2	P23188	Furin	Q9R0B9	Plod2	Q3UKJ7	Smu1
Q9Z0S1	Bpnt1	P16858	Gapdh	Q9R0E1	Plod3	Q62189	Snrpa
Q922D8	Mthfd1	G3UW34	Rpl9-ps6	Q99K51	Pls3	O88307	Sorl1
Q6ZQ38	Cand1	G3UXL2	Prps1l3	P55065	Pltp	Q60854	Serpinb6
Q60865	Caprin1	G3UZG5	Klhdc4	B2RXS4	Plxnb2	Q64674	Srm
P47757-2	Capzb	G3X956	Supt16	P97470	Ppp4c	Q64337	Sqstm1
P10605	Ctsb	G3X9T8	Cp	P30412	Ppic	P13609	Srgn
P97821	Ctsc	G5E866	Sf3b1	Q9CR16	Ppid	Q99MR6-	Srrt

P18242	Ctsd
Q9R013	Ctsf
P06797	Ctsl
Q9WUU7	Ctsz
P10148	Ccl2
O88430	Ccl22
P10855	Ccl3
P14097	Ccl4
P30882	Ccl5
P51670	Ccl9
P10810	Cd14
P15379-2	Cd44
P40240	Cd9
Q4VAA2	Cdv3
P04186	Cfb
P06909	Cfh
Q64433	Hspe1
Q68FD5	Cltc
Q9Z1Q5	Clic1
Q3UMW8	Cln5
P53996-2	Cnbp
P01027	C3
Q8CIE6	Copa
O89053	Coro1a
Q9WUM4	Coro1c
P07141	Csf1
P09920	Csf3
O35864	Cops5
Q9CWL8	Ctnnbl1
P10889	Cxcl2
Q9CXW3	Cacybp
Q7TMB8	Cyfip1
Q62426	Cstb
P21460	Cst3
D3YW09	Sf3a2
D3YW23	Cxcl10
D3YX34	Dctn1
D3YYD5	Vps29
D3Z1D6	Gm10335
Q61656	Ddx5
O35286	Dhx15
Q8R242-2	Ctbs
P62627	Dynlrb1

G5E8F1	N/A
Q8BHN3	Ganab
Q99PT1	Arhgdia
P17439	Gba
Q9CPV4	Glod4
O08795	Prkcsh
P28798	Grn
Q3THK7	Gmps
P10922	H1f0
P15864	Hist1h1c
P43274	Hist1h1e
Q9QZQ8-2	H2afy
P84228	Hist1h3b
H3BLJ9	Esd
H7BWX9	Sumo2
P01899	H2-D1
P01900	H2-D1
P06339	H2-T23
P01902	H2-K1
P01897	H2-L
P04441	Cd74
P70349	Hint1
P30681	Hmgb2
P97825	Hn1
Q61699-2	Hsph1
P11499	Hsp90ab1
Q8BM72	Hspa13
P63017	Hspa8
I7HIQ2	Cxcl16
P13597-2	Icam1
Q9JHJ8	Icoslg
P58044	Idi1
Q8BMJ3	Eif1ax
P60843	Eif4a1
Q8BGD9	Eif4b
P63073	Eif4e
Q9WUK2-2	Eif4h
P01575	Ifnb1
P08505	Il6
P24547	Impdh2
Q9D819	Ppa1
Q9JKF1	Iqgap1
O89051	Itm2b

Q61074	Ppm1g
P35700	Prdx1
Q61171	Prdx2
O08807	Prdx4
P11680	Cfp
Q08761	Pros1
P62334	Psmc6
Q9QUM9	Psma6
O55234	Psmb5
Q60692	Psmb6
Q8BG32	Psmd11
Q9D8W5	Psmd12
Q9WVJ2	Psmd13
Q3TXS7	Psmd1
Q8VDM4	Psmd2
O35226	Psmd4
B0V2N1-6	Ptprs
Q9JKF6	Pvrl1
P70698	Ctps1
Q148R4	Spink5
Q3TBV5	Il1rn
Q3TMX0	
Q3TUE1	Fubp1
Q3TUJ9	
Q542I8	Itgb2
Q60709	Aplp2
Q6P6I8	Sirpa
Q7M739	Tpr
Q8C253	Lgals3
Q8C605	Pfkp
Q8C845	Efhd2
Q8HWB2	H2-Q4
Q9CZN7	Shmt2
Q9DCC5	Cbx3
P63001	Rac1
Q05144	Rac2
P46061	Rangap1
P62827	Ran
P34022	Ranbp1
Q60972	Rbbp4
O89086	Rbm3
Q8VE37	Rcc1
Q8BK67	Rcc2

3	
Q6PDM2	Srsf1
Q62093	Srsf2
Q60864	Stip1
Q9CX34	Sugt1
Q9ER72	Cars
Q922B2	Dars
Q8CGC7	Eprs
Q9WUA2	Farsb
Q8BU30	Iars
Q99MN1	Kars
Q9D0I9	Rars
Q91WQ3	Yars
Q93092	Taldo1
P05213	Tuba1b
P68373	Tuba1c
P99024	Tubb5
Q922F4	Tubb6
P48428	Tbca
P10711	Tcea1
P11031	Sub1
P42932	Cct8
P63028	Tpt1
Q9R0Q7	Ptges3
Q80YX1-2	Tnc
Q62351	Tfrc
P04202	Tgfb1
Q62318	Trim28
P62075	Timm13
P26039	Tln1
P06804	Tnf
P41274	Tnfsf9
Q8BFY9-2	Tnpo1
P25119	Tnfrsf1b
Q9ER38	Tor3a
Q64514-2	Tpp2
Q9DCG9	Trmt112
Q99NH8	Trem2
Q62348	Tsn
P61087	Ube2k
Q9JMA1	Usp14
Q80X50-2	Ubap2l
Q8R317	Ubqln1
Q9Z1Z0-4	Uso1

Q91YW3	Dnajc3	J3QNB3	Adam17	Q9CYN9	Atp6ap2	P50516	Atp6v1a
Q6NZB0	Dnajc8	Q6WVG3	Kctd12	Q91VI7	Rnh1	P20152	Vim
Q8C255	Dpep2	L7N202	Gm16477	P47963	Rpl13	Q9EQH3	Vps35
Q8VDW0	Ddx39a	Q61792	Lasp1	P62717	Rpl18a	P61965	Wdr5
Q9Z1N5	Ddx39b	P35951	Ldlr	P67984	Rpl22	Q9ERK4	Cse1l
E9PVX6	Mki67	P16045	Lgals1	P62830	Rpl23	Q9JKB3-2	Ybx3
E9PX73	Ly9	P09056	Lif	P61255	Rpl26		

Supplementary Table 6 GOBPs regulated differentially by G9a and PHF8 in inflammation response

**Immune Response**		
ID	Gene Name	Gene symbol
P10810	CD14 antigen	Cd14
P04441	CD74 antigen (invariant polypeptide of major histocompatibility complex, class II antigen-associated)	Cd74
Q8HWB2	MHC class I like protein GS10	H2-Q4
P01887	beta-2 microglobulin	B2m
P10148	chemokine (C-C motif) ligand 2	Ccl2
O88430	chemokine (C-C motif) ligand 22	Ccl22
P10855	chemokine (C-C motif) ligand 3	Ccl3
P14097	chemokine (C-C motif) ligand 4	Ccl4
P30882	chemokine (C-C motif) ligand 5	Ccl5
P51670	chemokine (C-C motif) ligand 9	Ccl9
P10889	chemokine (C-X-C motif) ligand 2	Cxcl2
P09920	colony stimulating factor 3 (granulocyte)	Csf3
P01027	complement component 3; similar to complement component C3 prepropeptide, last	C3
P06909	complement component factor h; similar to complement component factor H	Cfh
P04186	complement factor B	Cfb
P11680	complement factor properdin	Cfp
Q9EQH2	endoplasmic reticulum aminopeptidase 1	Erap1
P01899, P01900, P01897	histocompatibility 2, D region; histocompatibility 2, D region locus 1	H2-D1
P01902	histocompatibility 2, K1, K region; similar to H-2K(d) antigen	H2-K1
P06339	histocompatibility 2, T region locus 23; similar to RT1 class Ib, locus H2-Q-like, grc region	H2-T23
Q9JHJ8	icos ligand	Icoslg
Q3TBV5	interleukin 1 receptor antagonist	Il1rn
P08505	interleukin 6	Il6
P09056	leukemia inhibitory factor	Lif
P35700	peroxiredoxin 1; predicted gene 7204	Prdx1
Q61171	peroxiredoxin 2	Prdx2
Q64695	protein C receptor, endothelial	Procr
Q64337	sequestosome 1	Sqstm1
Q7TQI3	similar to OTU domain, ubiquitin aldehyde binding 1; OTU domain, ubiquitin aldehyde binding 1	Otub1
P04202	transforming growth factor, beta 1	Tgfb1
P06804	tumor necrosis factor	Tnf
P41274	tumor necrosis factor (ligand) superfamily, member 9	Tnfsf9
P25119	tumor necrosis factor receptor superfamily, member 1b	Tnfrsf1b
**Translation**		
ID	Gene Name	Gene symbol

Q9D0I9	arginyl-tRNA synthetase	Rars
Q922B2	aspartyl-tRNA synthetase	Dars
Q9ER72	cysteinyl-tRNA synthetase	Cars
Q8BMJ3	eukaryotic translation initiation factor 1A, Y-linked	Eif1ax
Q8R1B4	eukaryotic translation initiation factor 3, subunit C; similar to Eukaryotic translation initiation factor 3, subunit 8	Eif3c
O70194	eukaryotic translation initiation factor 3, subunit D	Eif3d
Q9DCH4	eukaryotic translation initiation factor 3, subunit F	Eif3f
Q9Z1D1	eukaryotic translation initiation factor 3, subunit G	Eif3g
Q9QZD9	eukaryotic translation initiation factor 3, subunit I	Eif3i
Q9DBZ5	eukaryotic translation initiation factor 3, subunit K	Eif3k
Q8QZY1	eukaryotic translation initiation factor 3, subunit L	Eif3l
Q99JX4	eukaryotic translation initiation factor 3, subunit M	Eif3m
P60843	eukaryotic translation initiation factor 4A1	Eif4a1
Q8BGD9	eukaryotic translation initiation factor 4B	Eif4b
P63073	hypothetical LOC630527; eukaryotic translation initiation factor 4E; similar to eukaryotic translation initiation factor 4E	Eif4e
Q8BU30	isoleucine-tRNA synthetase	Iars
Q9WUA2	phenylalanyl-tRNA synthetase, beta subunit	Farsb
P62900	predicted gene 10191; predicted gene 7689; predicted gene 9401; similar to ribosomal protein L31; hypothetical protein LOC675768; predicted gene 13004; predicted gene 9228; predicted gene 10072; predicted gene 5437; predicted gene 9154; ribosomal protein L31; predicted gene 6670; predicted gene 8759	Rpl31
P62245	predicted gene 13048; predicted gene 11968; predicted gene 13253; predicted gene 14166; predicted gene 10196; similar to ribosomal protein S15a; predicted gene 5623; ribosomal protein S15A; similar to hCG1994130; predicted gene 7263	Rps15a
P41105	predicted gene 13226; predicted gene 15435; ribosomal protein L28; predicted gene 12938	Rpl28
P62717	predicted gene 15427; predicted gene 7602; predicted gene 7043; ribosomal protein L18A	Rpl18a
O89086	predicted gene 15453; RNA binding motif protein 3	Rbm3
Q9D8N0	predicted gene 4462; similar to eukaryotic translation elongation factor 1 gamma; predicted gene 9276; predicted gene 5525; eukaryotic translation elongation factor 1 gamma; similar to Elongation factor 1-gamma (EF-1-gamma) (eEF-1B gamma); predicted gene 4366	Eef1g
P14148	predicted gene 4754; ribosomal protein L7; predicted gene 12174; LOC100049084; predicted gene 6543; predicted gene 7059; predicted gene 4734	Rpl7
P10126	predicted gene 5869; predicted gene 7161; predicted gene 7105; predicted gene 5822; similar to eukaryotic translation elongation factor 1 alpha 1; predicted gene 6192; predicted gene 6392; predicted gene 6767; predicted gene 6170; predicted gene 6548; predicted gene 6789; eukaryotic translation elongation factor 1 alpha 1	Eef1a1
P61358	predicted gene 6599; predicted gene 6199; predicted gene 6341; predicted gene 6301; predicted gene 11518; similar to ribosomal protein L27; ribosomal protein L27; predicted gene 7053; predicted gene 11552; predicted gene 15730	Rpl27
P62855	predicted gene 7392; predicted gene 6654; ribosomal protein S26; predicted gene 10070; similar to ribosomal protein S26	Rps26
P62267	predicted gene 8624; predicted gene 9701; predicted gene 8467; predicted gene 15450; ribosomal protein S23; similar to yeast ribosomal protein S28 homologue; predicted gene 5148; similar to ribosomal protein S23; predicted gene 10054	Rps23
P14131	predicted gene 8731; predicted gene 7504; similar to Rps16 protein; ribosomal protein S16	Rps16
P61255	ribosomal protein L26; predicted gene 15772; predicted gene 9197; predicted gene 13268; predicted gene 11826; predicted gene 8253; predicted gene 7606	Rpl26
Q9D1R9	ribosomal protein L34; predicted gene 10154; similar to ribosomal protein L34; predicted gene 7800; predicted gene 4705	Rpl34
P62918	ribosomal protein L8; similar to 60S ribosomal protein L8	Rpl8
P62281	ribosomal protein S11; predicted gene 6202; predicted gene 6394; predicted gene	Rps11

	11741; similar to ribosomal protein S11	
P67984	similar to 60S ribosomal protein L22 (Heparin binding protein HBp15); ribosomal protein L22 pseudogene; predicted gene 6784; ribosomal protein L22	Rpl22
Q8CGC7	similar to Bifunctional aminoacyl-tRNA synthetase; glutamyl-prolyl-tRNA synthetase	Eprs
Q99MN1	similar to lysyl-tRNA synthetase; lysyl-tRNA synthetase	Kars
Q7M739	similar to nuclear pore complex-associated intranuclear coiled-coil protein TPR; translocated promoter region	Tpr
P47963	similar to ribosomal protein L13; predicted gene 10071; predicted gene 9026; predicted gene 12918; predicted gene 7290; predicted gene 5075; predicted gene 15710; similar to 60S ribosomal protein L13; predicted gene 7159; ribosomal protein L13	Rpl13
P62830	similar to ribosomal protein L23; similar to HL23 ribosomal protein; ribosomal protein L23; predicted gene 9794	Rpl23
Q9JJI8	similar to ribosomal protein L38; predicted gene 13020; ribosomal protein L38; predicted gene 4991; karyopherin (importin) alpha 2; predicted gene 9028; predicted gene 8129; predicted gene 7123; predicted gene 5832; predicted gene 10184; predicted gene 7379; predicted gene 10259	Rpl38
Q91WQ3	tyrosyl-tRNA synthetase	Yars
**Response to Wounding**		
ID	Gene Name	Gene symbol
Q07813	BCL2-associated X protein	Bax
P10810	CD14 antigen	Cd14
P10605	cathepsin B	Ctsb
P10148	chemokine (C-C motif) ligand 2	Ccl2
O88430	chemokine (C-C motif) ligand 22	Ccl22
P10855	chemokine (C-C motif) ligand 3	Ccl3
P14097	chemokine (C-C motif) ligand 4	Ccl4
P30882	chemokine (C-C motif) ligand 5	Ccl5
P10889	chemokine (C-X-C motif) ligand 2	Cxcl2
P01027	complement component 3; similar to complement component C3 prepropeptide, last	C3
P06909	complement component factor h; similar to complement component factor H	Cfh
P04186	complement factor B	Cfb
P11680	complement factor properdin	Cfp
Q542I8	integrin beta 2	Itgb2
P08505	interleukin 6	Il6
Q61171	peroxiredoxin 2	Prdx2
Q64695	protein C receptor, endothelial	Procr
Q08761	protein S (alpha)	Pros1
P04202	transforming growth factor, beta 1	Tgfb1
P06804	tumor necrosis factor	Tnf
P25119	tumor necrosis factor receptor superfamily, member 1b	Tnfrsf1b
**Cytoskeleton**		
ID	Gene Name	Gene symbol
Q61792	LIM and SH3 protein 1	Lasp1
P34022	RAN binding protein 1	Ranbp1
P63001	RAS-related C3 botulinum substrate 1	Rac1
Q05144	RAS-related C3 botulinum substrate 2	Rac2
P59999	actin related protein 2/3 complex, subunit 4	Arpc4
O89053	coronin, actin binding protein 1A	Coro1a
Q9WUM4	coronin, actin binding protein 1C; predicted gene 5790	Coro1c
B7FAU9	filamin, alpha	Flna
Q9R059	four and a half LIM domains 3	Fhl3
Q99K51	plastin 3 (T-isoform)	Pls3
P05213	predicted gene 3756; tubulin, alpha 1B; predicted gene 5620; similar to alpha-tubulin isotype M-alpha-2; predicted gene 14150; predicted gene 3226	Tuba1b

P97470	protein phosphatase 4, catalytic subunit	Ppp4c
Q6P6I8	signal-regulatory protein alpha	Sirpa
P26039	talin 1	Tln1
P99024	tubulin, beta 5	Tubb5
P68510	tyrosine 3-monooxygenase/tryptophan 5-monooxygenase activation protein, eta polypeptide	Ywhah
**Cell proliferation**		
ID	Gene Name	Gene symbol
Q07813	BCL2-associated X protein	Bax
P04441	CD74 antigen (invariant polypeptide of major histocompatibility complex, class II antigen-associated)	Cd74
P63001	RAS-related C3 botulinum substrate 1	Rac1
Q9ERK4	chromosome segregation 1-like (S. cerevisiae)	Cse1l
P04186	complement factor B	Cfb
Q542I8	integrin beta 2	Itgb2
P08505	interleukin 6	Il6
P35700	peroxiredoxin 1; predicted gene 7204	Prdx1
Q61171	peroxiredoxin 2	Prdx2
P24547	predicted gene 15210; inosine 5'-phosphate dehydrogenase 2	Impdh2
Q9R0Q7	predicted gene 9769; prostaglandin E synthase 3 (cytosolic); similar to Sid3177p; predicted gene 11893	Ptges3
P04202	transforming growth factor, beta 1	Tgfb1
P06804	tumor necrosis factor	Tnf
P25119	tumor necrosis factor receptor superfamily, member 1b	Tnfrsf1b
**mRNA Processing**		
ID	Gene Name	Gene symbol
Q8VDW0	DEAD (Asp-Glu-Ala-Asp) box polypeptide 39	Ddx39a
Q61656	DEAD (Asp-Glu-Ala-Asp) box polypeptide 5; predicted gene 12183	Ddx5
O35286	DEAH (Asp-Glu-Ala-His) box polypeptide 15	Dhx15
Q9Z1N5	HLA-B-associated transcript 1A	Ddx39b
P12023	amyloid beta (A4) precursor protein	App
Q99K48	non-POU-domain-containing, octamer binding protein; predicted gene 8806	Nono
P60335	poly(rC) binding protein 1	Pcbp1
Q62189	predicted gene 8655; predicted gene 5145; predicted gene 8587; small nuclear ribonucleoprotein polypeptide A; predicted gene 5161	Snrpa
P62305	small nuclear ribonucleoprotein E; predicted gene 6487	Snrpe
Q9D554	splicing factor 3a, subunit 3	Sf3a3
Q8VIJ6	splicing factor proline/glutamine rich (polypyrimidine tract binding protein associated); similar to PTB-associated splicing factor	Sfpq
Q6PDM2	splicing factor, arginine/serine-rich 1 (ASF/SF2); similar to splicing factor, arginine/serine-rich 1 (splicing factor 2, alternate splicing factor)	Srsf1
Q62093	splicing factor, arginine/serine-rich 2 (SC-35)	Srsf2
**Chemotaxis**		
ID	Gene Name	Gene symbol
P63001	RAS-related C3 botulinum substrate 1	Rac1
Q05144	RAS-related C3 botulinum substrate 2	Rac2
P10148	chemokine (C-C motif) ligand 2	Ccl2
O88430	chemokine (C-C motif) ligand 22	Ccl22
P10855	chemokine (C-C motif) ligand 3	Ccl3
P14097	chemokine (C-C motif) ligand 4	Ccl4
P30882	chemokine (C-C motif) ligand 5	Ccl5
P51670	chemokine (C-C motif) ligand 9	Ccl9
P10889	chemokine (C-X-C motif) ligand 2	Cxcl2
O89053	coronin, actin binding protein 1A	Coro1a
Q542I8	integrin beta 2	Itgb2
**RNA Splicing**		

ID	Gene Name	Gene symbol
Q8VDW0	DEAD (Asp-Glu-Ala-Asp) box polypeptide 39	Ddx39a
Q61656	DEAD (Asp-Glu-Ala-Asp) box polypeptide 5; predicted gene 12183	Ddx5
O35286	DEAH (Asp-Glu-Ala-His) box polypeptide 15	Dhx15
Q9Z1N5	HLA-B-associated transcript 1A	Ddx39b
Q99K48	non-POU-domain-containing, octamer binding protein; predicted gene 8806	Nono
Q62189	predicted gene 8655; predicted gene 5145; predicted gene 8587; small nuclear ribonucleoprotein polypeptide A; predicted gene 5161	Snrpa
P62305	small nuclear ribonucleoprotein E; predicted gene 6487	Snrpe
Q9D554	splicing factor 3a, subunit 3	Sf3a3
Q8VIJ6	splicing factor proline/glutamine rich (polypyrimidine tract binding protein associated); similar to PTB-associated splicing factor	Sfpq
Q6PDM2	splicing factor, arginine/serine-rich 1 (ASF/SF2); similar to splicing factor, arginine/serine-rich 1 (splicing factor 2, alternate splicing factor)	Srsf1
Q62093	splicing factor, arginine/serine-rich 2 (SC-35)	Srsf2
**RNA Aminoacylation**		
ID	Gene Name	Gene symbol
Q9D0I9	arginyl-tRNA synthetase	Rars
Q922B2	aspartyl-tRNA synthetase	Dars
Q9ER72	cysteinyl-tRNA synthetase	Cars
Q8BU30	isoleucine-tRNA synthetase	Iars
Q9WUA2	phenylalanyl-tRNA synthetase, beta subunit	Farsb
Q8CGC7	similar to Bifunctional aminoacyl-tRNA synthetase; glutamyl-prolyl-tRNA synthetase	Eprs
Q99MN1	similar to lysyl-tRNA synthetase; lysyl-tRNA synthetase	Kars
Q7M739	similar to nuclear pore complex-associated intranuclear coiled-coil protein TPR; translocated promoter region	Tpr
Q91WQ3	tyrosyl-tRNA synthetase	Yars
**Lymphocyte Activation**		
ID	Gene Name	Gene symbol
Q07813	BCL2-associated X protein	Bax
P04441	CD74 antigen (invariant polypeptide of major histocompatibility complex, class II antigen-associated)	Cd74
Q9JHJ8	icos ligand	Icoslg
Q542I8	integrin beta 2	Itgb2
Q61171	peroxiredoxin 2	Prdx2
P24547	predicted gene 15210; inosine 5'-phosphate dehydrogenase 2	Impdh2
P67984	similar to 60S ribosomal protein L22 (Heparin binding protein HBp15); ribosomal protein L22 pseudogene; predicted gene 6784; ribosomal protein L22	Rpl22
P04202	transforming growth factor, beta 1	Tgfb1

Supplementary Table 7 Primer Sequences for qPCR

Gene (mouse)	Primer Sequence	
	**Left (5' to 3')**	**Right (5' to 3')**
**IL1a**	GCAACGGGAAGATTCTGAAG	TGACAAACTTCTGCCTGACG
**IL1b**	GCCCATCCTCTGTGACTCAT	AGGCCACAGGTATTTTGTCG
**IFNb**	CCATCCAAGAGATGCTCCAG	GTGGAGAGCAGTTGAGGACA
**IL6**	CCGGAGAGGAGACTTCACAG	CAGAATTGCCATTGCACAAC
**IL10**	GGTTGCCAAGCCTTATCGGA	ACCTGCTCCACTGCCTTGCT
**GAPDH**	AACTTTGGCATTGTGGAAGG	ACACATTGGGGGTAGGAACA
**TNFa**	CCCCAAAGGGATGAGAAGTT	GTGGGTGAGGAGCACGTAGT

**PHF8**	AACACAACAAATGCTAATCT	AGAAGTTCCCTCCGAATGCT
**ADAM17**	AAGTGCAAGGCTGGGAAATG	CACACGGGCCAGAAAGGTT
**B2M**	CCGAACATACTGAACTGC	AGAAAGACCAGTCCTTGC
**CCL2**	AGGTCCCTGTCATGCTTCTG	TCTGGACCCATTCCTTCTTG
**CCL7**	AATGCATCCACATGCTGCTA	CTTTGGAGTTGGGGTTTTCA
**CCL9**	GGTCTGTCTGCCTCTTTTGC	GGGCTACACAGAGAAACCCT
**CCL22**	GCTCTCGTCCTTCTTGCTGT	GCAGGATTTTGAGGTCCAGA
**CD74**	ACGGCAAATGAAGTCAGAACA	AAGACTACTAATGGGTCAGAAATGG
**CFB**	CTCGAACCTGCAGATCCAC	TCAAAGTCCTGCGGTCGT
**CTSB**	GCCCGACCATTGGACAGAT	GCCCCAAATGCCCAACA
**CTSL1**	GACCGGGACAACCACTGTG	CCCATCAATTCACGACAGGAT
**CTSS**	AAGCGGTGTCTATGACGACCC	GAGTCCCATAGCCAACCACAA
**CTSZ**	CCTGTCCGGGAGGGAGAA	TGGTTGATAACGGCCTGGTC
**CXCL16**	TCCTTTTCTTGTTGGCGCTG	CAGCGACACTGCCCTGGT
**IFI30**	GTCAGCTGTACCAGGGAACG	GTCTGGGCTTTGTGGGACAT
**ITGA4**	TGCACCTCTTGTCTGTCTTGTT	GTGGCCTTAGCTCCTCCTCT
**LIF**	AATGCCACCTGTGCCATACG	CAACTTGGTCTTCTCTGTCCCG
**TNFSF9**	GCAAGCAAAGCCTCAGGTAG	TCCAGGAACGGTCCACTAAC
**TREM2**	AGAGTGTGGTGACGGGTTCC	TATGACGCCTTGAAGCACTG

REFERENCES:

1. Meissner, F., Scheltema, R.A., Mollenkopf, H.J. & Mann, M. Direct proteomic quantification of the secretome of activated immune cells. *Science* **340**, 475-8 (2013).
2. Liu, C. et al. A chromatin activity-based chemoproteomic approach reveals a transcriptional repressome for gene-specific silencing. *Nat Commun* **5**, 5733 (2014).
